# Total Synthesis of (+)-Malbrancheamide B Employing a Bioinspired Strategy

**DOI:** 10.1021/acs.orglett.6c00246

**Published:** 2026-02-27

**Authors:** M. Aurelia Bosi, Radek Pohl, Ullrich Jahn

**Affiliations:** Institute of Organic Chemistry and Biochemistry of the Czech Academy of Sciences, 16000 Prague 6, Czech Republic

## Abstract

Prenylated indole alkaloids are a class of secondary metabolites isolated from various terrestrial and marine fungi. The common complex structure, featuring a diazabicyclo[2.2.2]­octane scaffold fused to a tetrahydrocarbazole unit, and the numerous biological activities make these compounds attractive synthetic targets. In the present work an asymmetric total synthesis of (+)-malbrancheamide B is reported. Key steps are bioinspired diketopiperazine (DKP) assembly, a stereoselective oxidative radical cyclization, which gives access to the three-dimensional bridged diketopiperazine precursor and an intramolecular Friedel–Crafts cyclization providing the hexacyclic skeleton of malbrancheamide B.

## Graphical abstract

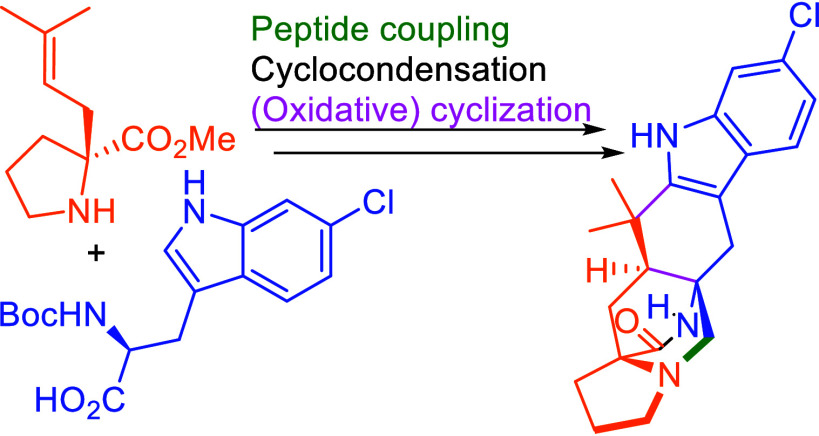

Bridged (di)­ketopiperazine alkaloids,^1,2^ such as malbrancheamide B (**1**),^3^ stephacidin A (**2**),^4^ paraherquamide A (**3**)^5^ or asperparaline C (**4**),^6^ are a part of the broad class of terpenylated indole alkaloids predominately isolated from a variety of terrestrial and marine fungi (Figure 1A).^1,2^ These natural products exhibit a diketopiperazine scaffold and a prenyl bridge connecting its 3- and 6-positions, thus forming a unique diazabicyclo[2.2.2]­octane system, which is linked in fused or spiro mode to indole, oxoindole, or indoxyl rings.^1,2^ Other prominent members are the brevianamides,^7^ marcfortines,^8^ notoamides,^9^ or taichunamides^10^ (not shown). In the malbrancheamides, marcfortines, asperparalines, and paraherquamides one of the two amide carbonyl groups is reduced to a methylene. The alkaloids display a broad range of biological activities, being anthelmintic, insecticidal, cytotoxic, or antibacterial,^1,2^ whereas malbrancheamide B is a calmodulin inhibitor and vasorelaxant.^3c,3d^

**Figure 1:**
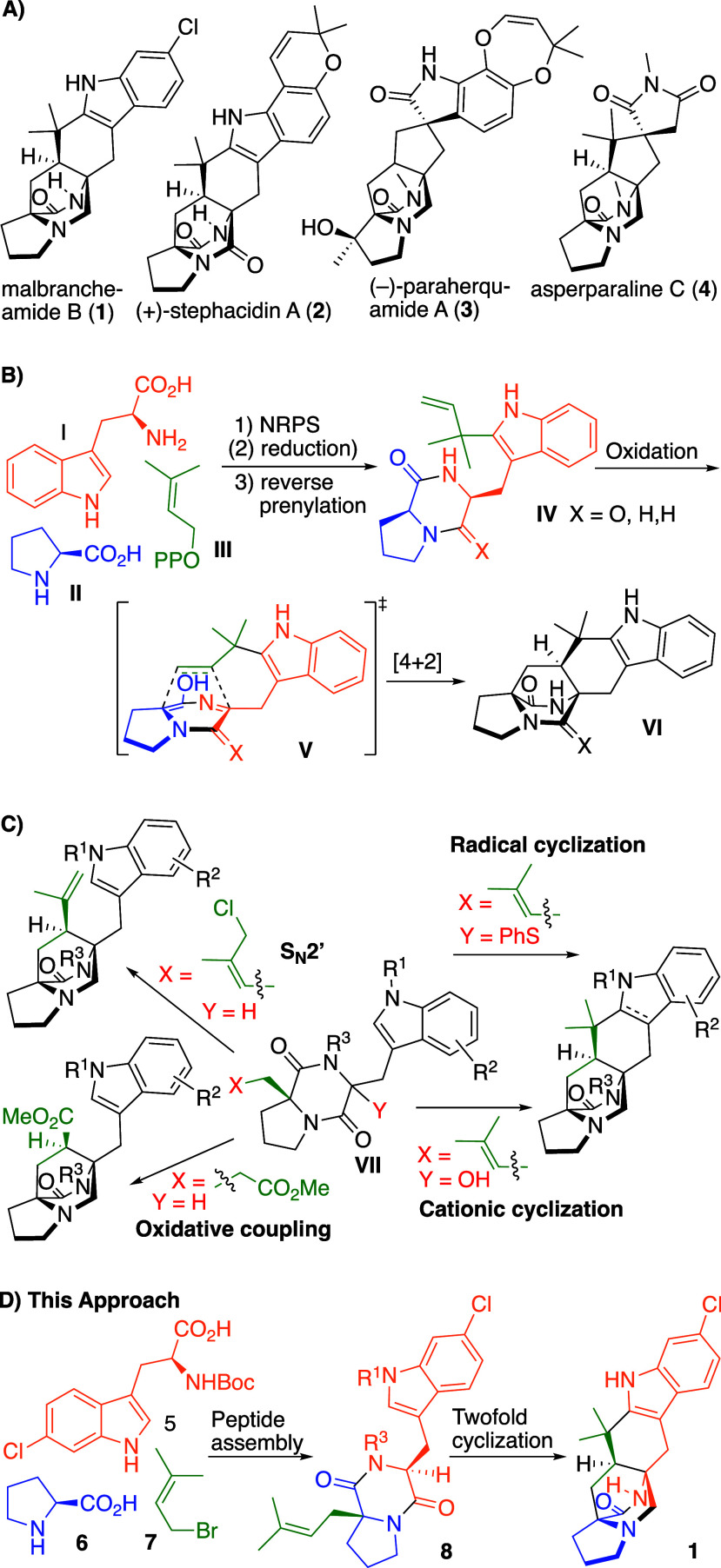
Diketopiperazine alkaloids containing the di­aza­bi­cyclo­[2.2.2]­octane core. A) Prominent members. B) Proposed biosynthesis. C) Known general synthetic approaches to bridged diketopiperazine alkaloids. D) Envisaged direct approach avoiding prefunctionalization.

Several studies have focused on the biosynthesis of prenylated indole alkaloids (Figure 1B). A general pathway involving nonribosomal dipeptide synthesis (NRPS) from tryptophan **I** and proline **II**, reverse prenylation with **III** in 2-position of the tryptophan unit, and an oxidative intramolecular Diels–Alder reaction of DKP **IV** to construct the bridged core structure **VI** was substantiated^1,11–15^ and is supported by *ab initio* calculations.^16^ This occurs for most natural products with *syn* diastereoselectivity of the hydrogen atom and the bridging secondary amide, although a few alkaloids have *anti* configuration.^1,13^

Their complex structures and numerous biological activities make these natural products attractive targets in total synthesis.^2,13,15,17^ Five general strategies have been applied for total syntheses of bridged diketopiperazine alkaloids **VI**. The Williams,^13,15,17^ Scheerer^18^ and recently the Banwell^19^ groups developed biomimetic intramolecular Diels–Alder cycloadditions (cf. Figure 1B). This approach leads, however, mostly to racemic alkaloids and *syn*/*anti* mixtures since the stereochemical information in the DKP precursor **IV** is destroyed during generation of azadiene **V**, unless residing stereocenters exert a directing effect.^17,18b^ All other approaches are intermediate-based and relied on prefunctionalized precursors **VII** (Figure 1C).^2,17a,17b^ Williams and colleagues used chloroprenyl precursors in a stereoselective S_N_2′ cyclization to construct the bridge for the synthesis of several bridged DKP alkaloids.^17a,17b^ An oxidative ester enolate-indole coupling was used by Baran et al.^20^ in the total synthesis of **2**. Simpkins and co-workers employed in contrast bridge-head functionalized diketopiperazines and applied them in radical cascade cyclizations to hexacycles with an indoline unit, which was transformed to stephacidin A (**2**),^21^ or cationic cascade cyclizations providing brevianamide B and *ent*-malbrancheamide B.^22^

**Scheme 1:**
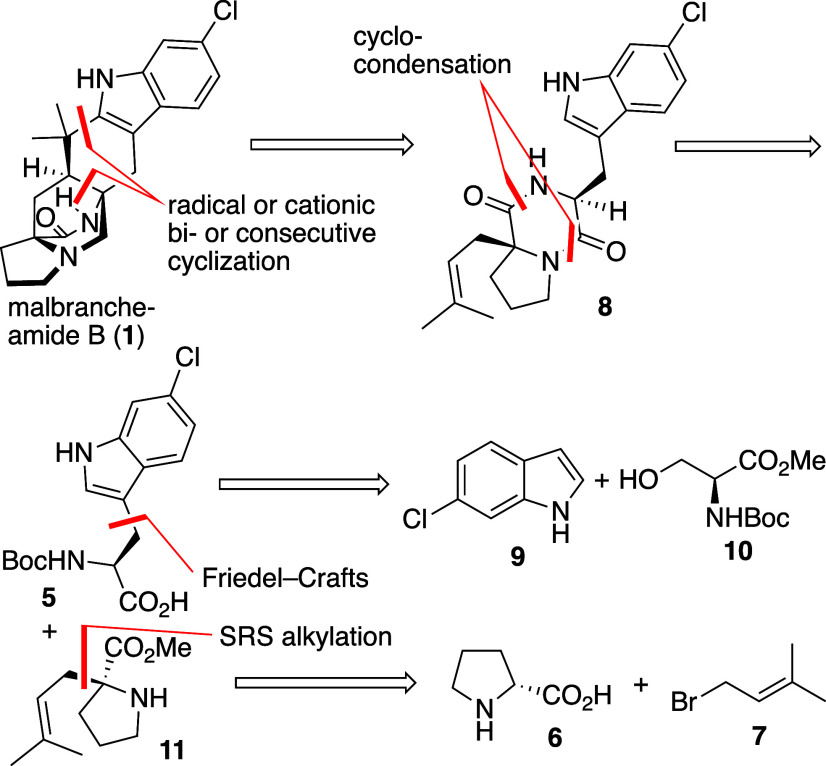
Bioinspired Retrosynthesis of 1

Based on our experience in the synthesis of diketopiperazines,^23,24^ we hypothesized that a bioinspired direct approach for the total synthesis of malbrancheamide B (**1**) may be accomplished. This is enabled by a strategy relying on peptide assembly using chlorotryptophan **5**, proline **6** and prenyl bromide **7** to achieve a direct access to prenylated diketopiperazine precursor **8** (Figure 1D), which was envisaged to provide the natural product **1** by an oxidative bicyclization or consecutive cyclization reactions. Here we report the large potential of this approach, which results in a short total synthesis of the natural product.

Reducing this bioinspired approach to practice requires the following retrosynthesis (Scheme 1). The removal of the oxo group to accomplish the total synthesis of malbrancheamide B (**1**) is proposed to take place as the last step. Further disconnection at the quaternary center next to the indole unit and at the diketopiperazine bridge by a radical bicyclization or consecutive radical and cationic cyclizations calls for diketopiperazine **8**. A retro-cyclocondensation leads to the corresponding amino acid derivatives **5** and **11**. Chlorotryptophan **5** can be traced to chloroindole **9** and serine ester **10**; this mimics the biosynthesis of tryptophan. 2-Prenylproline ester **11** will be approached from d-proline **6** and prenyl bromide **7** using Seebach’s “self-regeneration of stereocenters (SRS)” methodology.^25^

The synthesis commenced with commercial N-Boc-l-serine methyl ester **10**, which was transformed into sulfamidate **12** by treatment with thionyl chloride in the presence of pyridine, followed by oxidation with sodium periodate and a catalytic amount of ruthenium­(III) chloride in analogy to Zervosen et al. (Scheme 2).^26^ Chloroindole **9** was metalated at nitrogen according to the method developed by Piersanti and co-workers^27^ and the resulting indolylcopper intermediate was treated with **12** leading to chlorotryptophan methyl ester **13**. The moderate yield is in line with those observed by Piersanti et al.;^27^ however, this approach is the most straightforward and general to obtain substituted tryptophans. Other pursued approaches starting from tryptophan methyl ester are longer and not higher yielding (see the Supporting Information (SI)). Saponification of the methyl ester with lithium hydroxide provided the N-Boc amino acid **5**. The second amino acid building block, α-prenyl proline methyl ester **11**, was prepared from d-proline using Seebach’s SRS strategy in good yield,^25,28^ The subsequent peptide coupling was conducted using *N*-ethoxycarbonyl-2-ethoxy-1,2-dihydroquinoline (EEDQ) as coupling agent in the absence of base, to prevent epimerization at the tryptophan unit, affording dipeptide **14** as a single diastereomer in good yield.

**Scheme 2:**
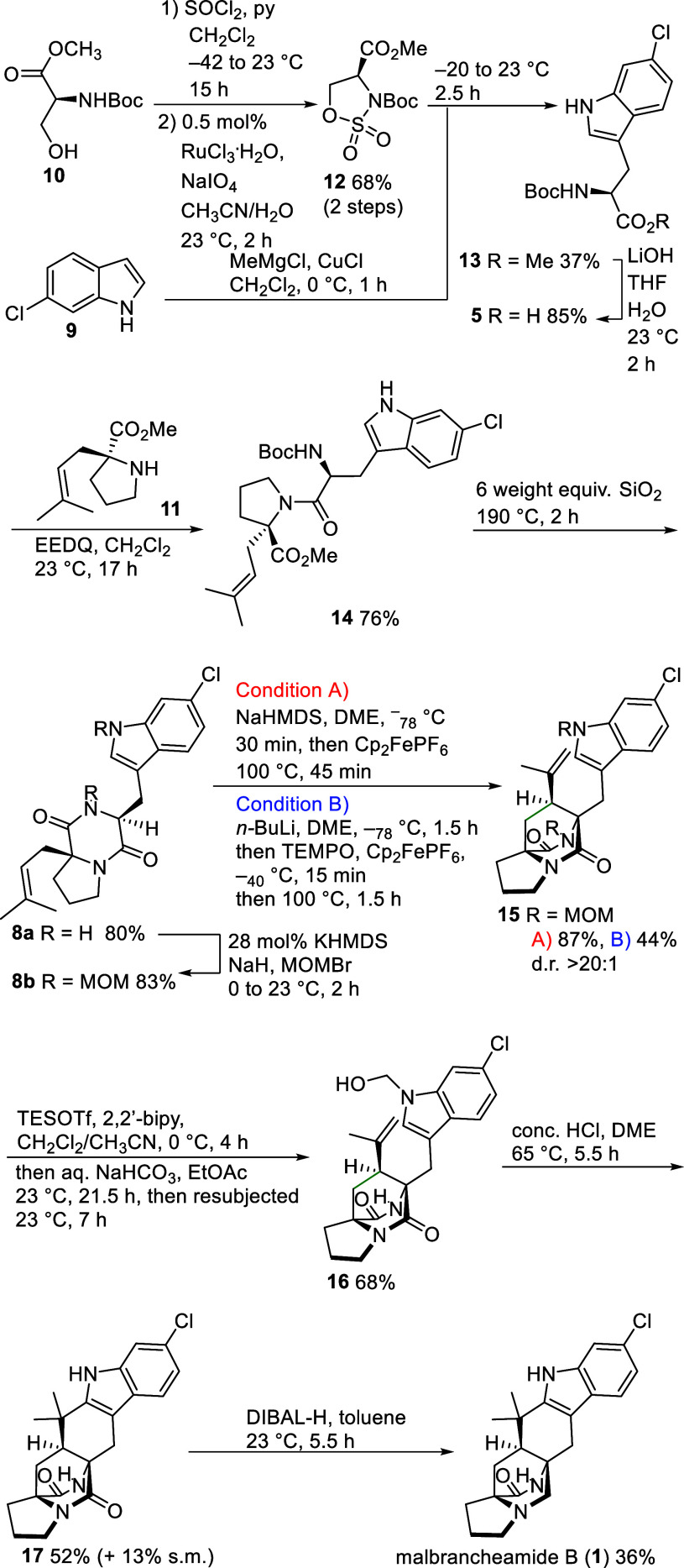
Total Synthesis of Malbrancheramide B (1)

The thermal cyclization of dipeptide **14** at high temperature on silica gel under strictly inert conditions provided diketopiperazine **8a** in very good yield as a single diastereomer. This method is practical since absorbing the dipeptide at silica gel prevents substrate diffusion, side reactions and allows a very simple purification.^23^ Protection of both the amide and the indole nitrogen atoms in **8a** was necessary at this point (vide infra), the methoxymethyl (MOM) group was chosen because of its stability under basic conditions required in the next step. In the event, twofold MOM protection of DKP **8a** was not trivial; standard conditions with NaH^24^ gave only 27% yield (not shown). However, introducing a substoichiometric amount of a soluble base such as potassium bis­(trimethylsilyl)­amide (KHMDS) in tetrahydrofuran (THF) facilitated the protection significantly, providing a very good yield of **8b**. Acyl or carbamate protecting groups were not applicable since a Chan–Lam-type rearrangement leading to ring contraction is known to take place when deprotonating acyl-protected DKPs at the α-position.^29^ The application of a protected precursor for the cyclization is mandatory, since otherwise other pathways prevail (vide infra, Scheme 4).

The crucial oxidative bridge-forming cyclization was performed by two methods. Reasoning that the DKP enolate precursor may be thermally rather robust, under condition A) a ferrocenium ion-mediated oxidative cyclization was leveraged to form the diazabicyclo[2.2.2]­octane ring system. Diketopiperazine **8b** was deprotonated at low temperature and subsequently heated to 100 °C and ferrocenium hexafluorophosphate was added to trigger the cyclization. This proved indeed highly practical and provided bridged DKP **15** essentially as a single *syn-*diastereomer. In contrast a 2,2,6,6-tetramethylpiperidin-1-oxyl (TEMPO)-intercepted cyclization (condition B)), which was inspired by a previous total synthesis of the bridged DKP unit in asperparaline C (**4**),^24^ was much less efficient. Single-electron oxidation of the lithium enolate of **8b** at low temperature in the presence of TEMPO results in the formation of an extremely labile adduct, which decays on slow warming. To facilitate the cyclization, the reaction mixture was transferred to a preheated bath triggering the radical cyclization and hydrogen atom transfer. The isopropenyl-substituted bridged DKP **15** was isolated in moderate 44% yield, but also as a single *syn-*diastereomer.

The mechanism and diastereoselectivity of the oxidative cyclizations of **8b** can be explained as follows (Scheme 3). Deprotonation of diketopiperazine **8b** generates the corresponding enolate **8bNa**. Subsequent single-electron oxidation leads to selective radical generation at the α-position of the tryptophan unit. The subsequent 6-exo cyclization proceeds essentially exclusively via *syn-*transition state *syn*-**TS** because the orientation of the cyclizing prenyl unit toward the less demanding carbonyl group is strongly preferred over the alternative arrangement in *anti*-**TS** where interactions with the methylene groups of the MOM and tryptophan units may hamper reaching the cyclization transition state. The cyclized tertiary alkyl radical **18** is subsequently oxidized to the corresponding carbocation **19**.

**Scheme 3:**
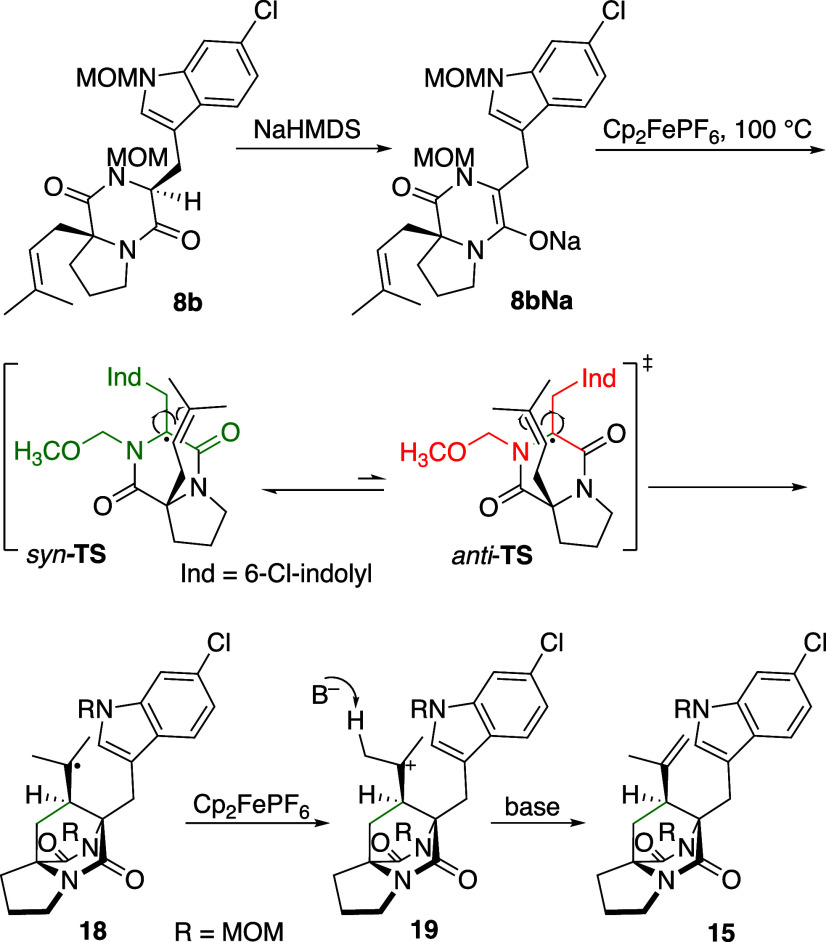
Mechanistic Course and Diastereoselectivity of the Oxidative Radical Cyclization of 8

At this stage an intramolecular Friedel–Crafts-type cyclization was envisaged to take place providing the complete malbrancheamide skeleton. However, the basic conditions of the reaction favored deprotonation to isopropenyl diazabicyclo[2.2.2]­octandione **15**. Under the previously established cyclization conditions^24^ TEMPO couples with the initial DKP radical at low temperature. Heating the labile adduct also triggers the cyclization via *syn-* **TS**; however, this proved to be less effective (not shown).

To complete the total synthesis, compound **15** was initially subjected to aqueous HCl to initiate the crucial cationic cyclization and concomitant MOM deprotection (Scheme 2). However, this provided an unexpected outcome (vide infra, Scheme 4). Therefore, initial MOM deprotection was mandatory, which was not trivial. After much experimentation (see the SI), reaction with triethylsilyl triflate using 2,2′-bipyridine as a formaldehyde scavenger proved optimal.^30^ The resulting mixture was subjected to basic conditions leading to slow, but complete lactam deprotection after resubjection of the reaction mixture to aqueous NaHCO_3_ solution, but the hydroxymethyl group at the indole unit was resistant to removal. Bridged diketopiperazine **16** subsequently cyclized in the presence of hydrochloric acid giving oxomalbrancheamide B **17** in 52% yield; 13% of starting material (s.m.) was recovered under the conditions. Gratifyingly, the remaining hydroxymethyl group was also removed under the cyclization conditions. At this stage the total synthesis converged with Simpkins’s^22^ and Scheerer’s^18b^ total syntheses after only nine steps. A final diisobutylaluminum hydride (DIBAL-H) reduction according to the literature^18b^ provided malbrancheamide B (**1**) after 10 steps in 1.2% overall yield.

**Scheme 4:**
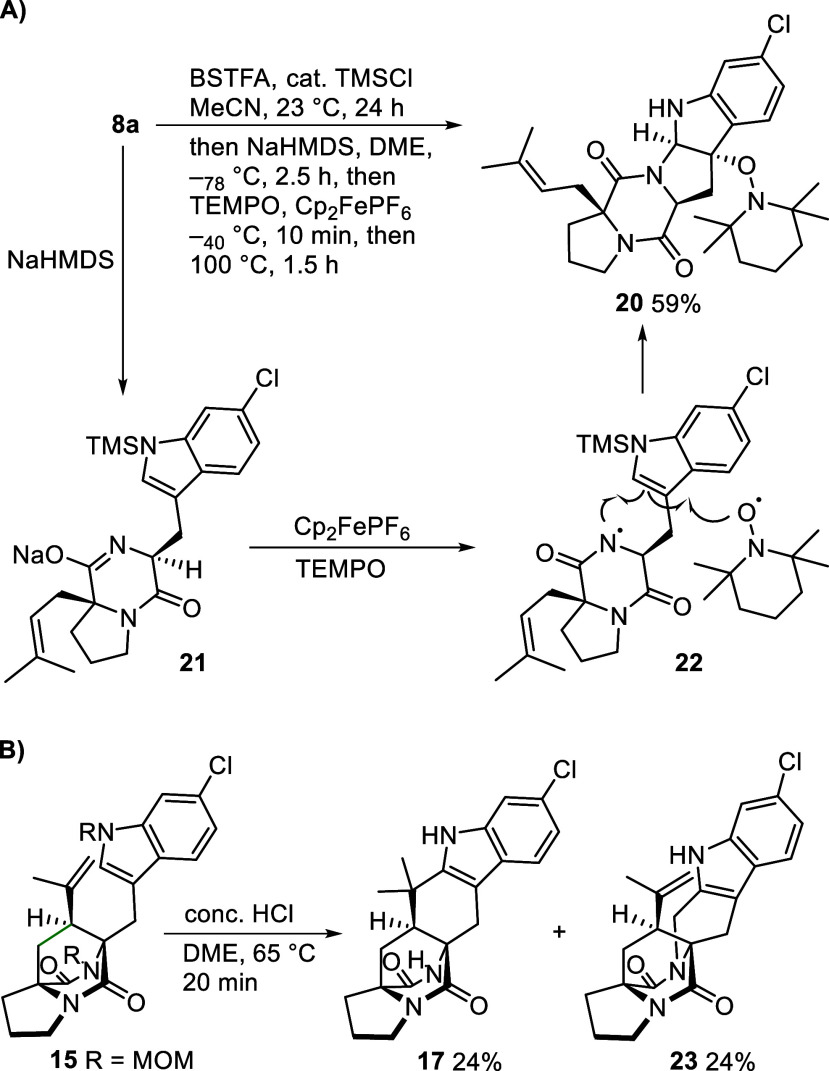
Divergent Reactivities of Diketopiperaazines 8a and 15: A) BSTFA Monoprotection/Cyclization to Pentacycle 20; B) Divergent Cyclization of Bis­(MOM)-Protected Bridged DKP 15

Several investigations support the pursued strategy. In parallel to the successful strategy an in situ-protection/deprotection methodology was tested (Scheme 4A).^31^ In short, diketopiperazine **8a** was treated with an excess bis­(trimethylsilyl) trifluoroacetimidate (BSTFA) in the presence of catalytic chlorotrimethylsilane. After evaporation, the resulting protected diketopiperazine intermediate was deprotonated with sodium bis­(trimethylsilyl)­amide (NaHMDS) and subjected to cyclization condition B) (cf. Scheme 2). However, pentacyclic product **20** was isolated in 59% yield instead of the expected bridge formation.

This suggests that the diketopiperazine NH in **8a** is not protected under the reaction conditions. Therefore, on deprotonation with NaHMDS amidate **21** was generated, which is oxidized to amidyl radical **22**. A rare radical 5-endo cyclization and coupling with TEMPO provides the product. Compound **20** is related to DKP-bound pyrroloindoline alkaloids, another attractive class of diverse biologically active alkaloids.^32^ The presence of the alkoxyamine group might allow further diversification toward the synthesis of these target molecules.

A direct Friedel–Crafts-type cyclization of bis­(MOM)-protected bridged diketopiperazine **15** was also entertained (Scheme 4B). However, an inseparable 1:1 mixture of the desired cyclization product **17** and indole aminomethylation product **23** was isolated. This shows that MOM protection has to precede cyclization to avoid the formation of a reactive N-acyliminium ion, which competes for the indole with the intramolecular Friedel–Crafts-type cyclization.

In conclusion, an asymmetric bioinspired total synthesis of **1** has been achieved over 10 steps in 1.2% overall yield. Key steps are a biomimetic dipeptide assembly, diketopiperazine formation, and a diastereoselective oxidative radical cyclization which enables the construction of the bridged diketopiperazine motif by reverting the polarity at the α-position of the amide and forming a new C–C bond. The full skeleton was assembled by an intramolecular Friedel–Crafts-type cyclization. This blueprint will serve to approach other relevant bridged indolodiketopiperazine alkaloids and their biological investigation.

## Supplementary Material



## Data Availability

The data underlying this study are available in the published article and its Supporting Information.

## Notes

Experimental procedures, characterization data and NMR spectra (PDF)
