# Supplementary material for: Total Synthesis of (+)-Malbrancheamide B Employing a Bioinspired Strategy
Source: Org Lett. 2026 Feb 27;28(10):3214–9. doi: 10.1021/acs.orglett.6c00246 (PMC12993921; doi:10.1021/acs.orglett.6c00246)

Supporting information

Total Synthesis of (+)-Malbrancheamide B Employing a Bioinspired Strategy

M. Aurelia Bosi, Radek Pohl, Ullrich Jahn*

Institute of Organic Chemistry and Biochemistry of the Czech Academy of Sciences, Flemingovo náměstí 2, 16000 Prague, Czech Republic.

Table of Contents

Initial approach to synthesize protected chlorotryptophan methyl ester **13** S3

Summary of attempted MOM-deprotection reactions S4

General information S6

Experimental procedures and characterization S8

References S29

Copies of ^{1}H and ^{13}C NMR spectra S31

1. Initial approach to synthesize protected chlorotryptophan methyl ester 13

The approach to chlorotryptophan was pursued by several pathways. In parallel to the most efficient pathway, an alternative commenced from tryptophan methyl ester hydrochloride **S1**, which was Boc-protected providing methyl ester **S2** (Scheme S1A). Introduction of the triisopropylsilyl group at the indole nitrogen atom and borylation at the 6-position were performed using known procedures that have been applied to the same substrate.[1,2] A minor amount of C-5 substituted tryptophan (**S4′**), inseparable from the desired regioisomer, was also obtained. Chlorination with $CuCl_2$ at high temperature[3] provided a mixture of **S5** and **S5′** but the yield remained moderate. Removal of the TIPS group would provide chlorotryptophan methyl ester **13** over five steps. In parallel another protocol published by Hartwig *et al.*,[4] a regioselective C-H silylation leading to **S6** as an alternative precursor was attempted**;** however, only starting methyl ester **S3** was recovered (Scheme S1B). In conclusion, the synthetic sequence that commences from 1H-6-chloroindole and N-Boc L-serine methyl ester was chosen since the synthesis is overall shorter and reliable.

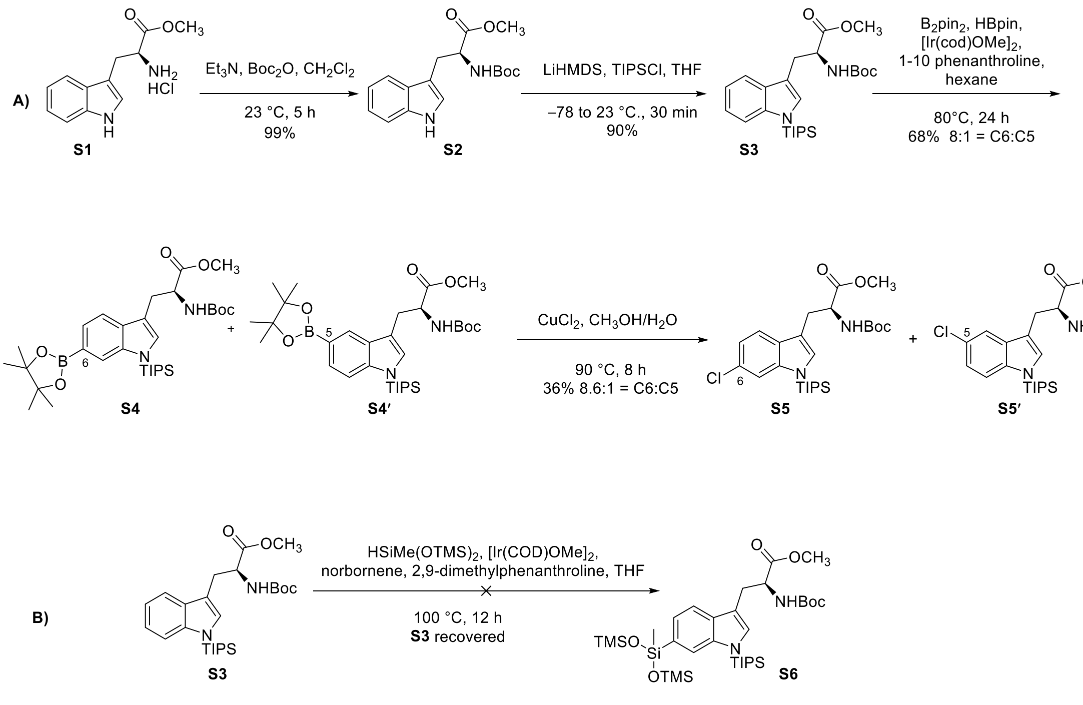

Scheme S1. A) Preparation of N-TIPS chlorotryptophan methyl ester **S5**. B) Attempted iridium-catalyzed silylation of methyl ester **S3**.

2. Summary of attempted MOM-deprotection reactions

The deprotection of the MOM groups from DKP **15** required much experimentation. The conditions and results are summarized in Table S1. When diketopiperazine **15** was treated with bromocatecholborane (Table S1, Entry 1),[5] decomposition was observed. Using Lewis acids such as BCl_3 and BBr_3 in the presence of *p*-xylene as a formaldehyde scavenger[6] led either to an inseparable mixture (Entry 2) or the starting material was recovered (Entries 3 and 4). When **15** was subjected to aq. 2 M HCl solution, in the presence or absence of sodium *p*-toluenesulfinate as a formaldehyde scavenger,[7] the starting material was recovered (Entries 5 and 6).

Table S1. Summary of unsuccessful conditions for MOM group removal from bridged DKP **15**.

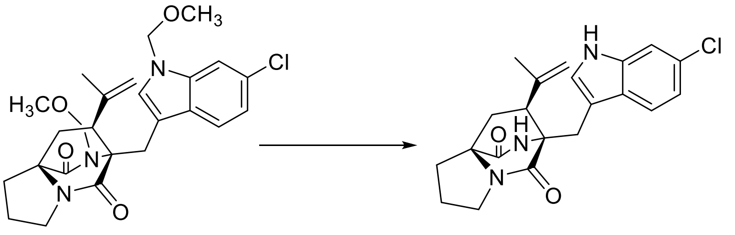

15 **S7**

Entry	Lewis/Brønsted acid (equiv.)	Additive (equiv.)	Solvent	Temperature (°C)	Reaction time (h)	Conversion to S7 (%)
1	Bromocatecholborane (2.6)	-	CH_2Cl_2	–20 to 0	1	decomposition
2	BCl_3 (0.5)	*p*-xylene (10)	CH_2Cl_2	–78	0.5	complex mixture
3	BBr_3 (1)	*p*-xylene (10)	CH_2Cl_2	–78	0.5	**15** recovered
4	BBr_3 (2)	*p*-xylene (10)	CH_2Cl_2	–78	0.5	**15** recovered
5	2 M HCl (4.76)	*p*-CH_3PhSO_2Na (4.4)	CH_3CN	23	1	**15** recovered
6	2 M HCl (4.76)	-	DME	23	2	**15** recovered

When the deprotection was tested with $ZnBr_2$ in the presence of EtSH,[8] the bridged diketopiperazine resulting from twofold nucleophilic substitution of the methoxy group with the ethylthio group was isolated after work-up (Scheme S2). This proves that MOM cleavage proceeds through the formation of the N,N'-bis(iminium) intermediate (**S8**), which undergoes nucleophilic attack by the thiolate. When the reaction was conducted in absence of EtSH olefin **15** was recovered.

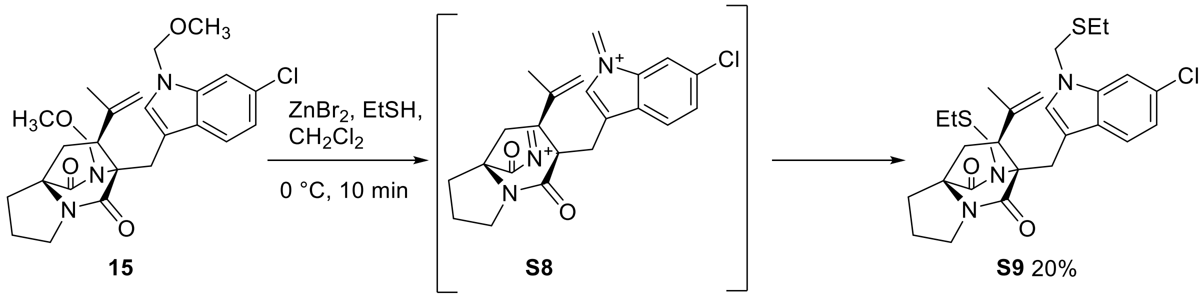

Scheme S2. Attempted MOM-deprotection using $ZnBr_2$ in the presence of EtSH, leading to DKP **S9**.

3. General information

All reactions were conducted under a nitrogen atmosphere. DME, THF, toluene and CH_2Cl_2 were dried using standard methods under an argon atmosphere. MeOH was dried over magnesium in the presence of iodine and distilled under a nitrogen atmosphere. Commercially available extra dry 99.9+% acetonitrile was used. TLC plates POLYGRAM SIL G/UV_{254} (Macherey-Nagel) were used for monitoring reactions, and visualization was performed either by UV light (254 nm) or chemical staining with potassium permanganate or anisaldehyde. Flash column chromatographic separations were performed on silica gel 60 (Fluka, 230-400 mesh). ^{1}H and ^{13}C NMR spectra were recorded on Bruker Avance instruments at 400.1, 500 or 600 MHz for ^{1}H NMR or 100.6, 126 or 151 MHz for ^{13}C NMR spectroscopies, respectively. Connectivity was determined by ^{1}H-^{1}H COSY and HMBC experiments. ^{13}C NMR assignments were obtained from HSQC experiments. IR spectra were taken on a Bruker ALPHA FT-IR spectrometer as neat samples using an ATR device. ESI mass spectra were obtained on a Thermo Fisher Scientific LCQ Fleet spectrometer, sample concentration approx. 1 µg/mL, spray voltage pos. mode: 3.3 kV. HRMS spectra were measured on a Waters Q-Tof micro spectrometer, resolution: 100000. Optical rotations were measured on an Autopol IV instrument (Rudolph Research Analytical, Flanders, USA).

Cautionary notes for using harmful substances

Diisobutylaluminum hydride (DIBAL-H): Highly flammable liquid and vapor. Catches fire spontaneously if exposed to air. Releases flammable gases on contact with water, which may ignite spontaneously. May be fatal if swallowed and enters airways. Causes severe skin burns and eye damage. Keep away from heat, hot surfaces, sparks, open flames and other ignition sources. Handle and store contents under inert gas and protect from moisture. Wear protective gloves, protective clothing, eye and face protection.

Conc. hydrochloric acid (HCl): May be corrosive to metals. Causes severe skin burns and eye damage. May cause respiratory irritation when breathing mist or vapors. Use only in a well-ventilated area and wear protective gloves, protective clothing, eye and face protection.

***N*,*O*-Bis(trimethylsilyl)trifluoroacetamide (BSTFA)**: Highly flammable liquid and vapor. Harmful if swallowed. Causes severe skin burns and eye damage. Keep away from

heat, hot surfaces, sparks, open flames and other ignition sources. Keep container tightly closed. Wear protective gloves, protective clothing, eye and face protection.

Potassium bis(trimethylsilyl)amide (KHMDS) in THF: Highly flammable liquid and vapor. Harmful if swallowed. Causes severe skin burns and eye damage. May cause respiratory irritation, drowsiness or dizziness. Suspected of causing cancer. Keep away from heat, hot surfaces, sparks, open flames and other ignition sources. Wear protective gloves, protective clothing, eye and face protection.

Pyridine: Highly flammable liquid and vapor. Harmful if swallowed, inhaled, or in contact with skin. Severely irritating to eyes and skin; vapors irritate the respiratory tract. Keep away from heat, hot surfaces, sparks, open flames and other ignition sources. Wear protective gloves, protective clothing, eye and face protection.

Sodium bis(trimethylsilyl)amide (NaHMDS) in THF: Causes severe skin burns and eye damage. Do not breathe dust of the solid. Wear protective gloves, protective clothing, eye and face protection.

Sodium metaperiodate ($NaIO_4$): May cause fire or explosion as a strong oxidizing reagent. Causes severe skin burns and eye damage. Causes damage to organs (especially thyroid) after prolonged or repeated exposure. Keep away from heat, hot surfaces, sparks, open flames and other ignition sources. Do not breathe dust. Avoid release to the environment because very toxic to aquatic life with long lasting effects. Wear protective gloves, protective clothing eye and face protection.

Thionyl chloride ($SOCl_2$): Harmful if swallowed. Causes severe skin burns and eye damage. Toxic if inhaled. May cause respiratory irritation. Reacts violently with water, this liberates toxic gases. Avoid breathing mist or vapors. Wear protective gloves, protective clothing, eye and face protection.

4. Experimental procedures and characterization

(*S*)-*N*$^{\alpha}$-(*tert*-Butoxycarbonyl)-6-chloro-1-(triisopropylsilyl)tryptophan methyl ester (S5)

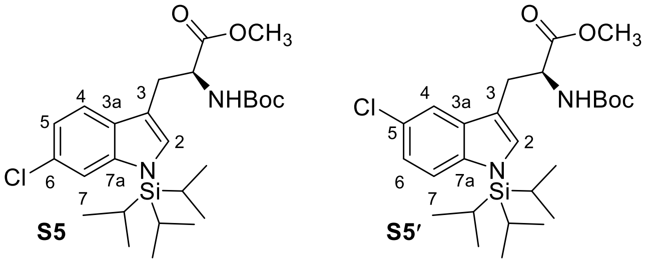

S5 **S5′**

Compound **S5** was prepared[3] from known compound **S4** (8:1 = **S4**:**S4′**), which was synthesized from L-tryptophan methyl ester hydrochloride (**S1**) applying a published protocol.[1,2] **S5** was obtained as an 8.6:1 mixture with compound **S5′**. **MS ESI+ *m/z*, (%)**: 1043/1041/1039 (5/31/60, $[2M+Na]^+$), 533/531 (36/100, $[M+Na]^+$), 455/453 (4/14, $[(M–isobutene)+H]^+$), 433/431 (2/6, $[(M–isobutene–CO_2)+Na]^+$), **HRMS ESI+ *m/z***, ($[M+Na]^+$): calcd. for $C_{26}H_{41}O_4N_2{}^{35}ClNaSi$: 531.2416, found: 531.2418. Calcd. for $C_{26}H_{41}O_4N_2{}^{37}ClNaSi$: 533.2393, found: 533.2393. **S5**: **^{1}H NMR (400 MHz, $CDCl_3$)**: δ = 7.43-7.41 (m, 2H, H-4, H-7), 7.08 (dd, *J* = 8.6, 1.7 Hz, 1H, H-5), 7.00 (s, 1H, H-2), 5.04 (br d, *J* = 8.2 Hz, 1H, $N\underline{H}CHCH_2$), 4.65-4.60 (m, 1H, $NHC\underline{H}CH_2$), 3.72 (s, 3H, OCH_3), 3.28-3.18 (m, 2H, $NHCHC\underline{H}_2$), 1.65 (sept, *J* = 7.5 Hz, 3H, $Si(C\underline{H}(CH_3)_3)$, 1.43 (s, 9H, $OC(CH_3)_3$), 1.131 (d, *J* = 7.5 Hz, 9H, $Si(CH(C\underline{H}_3)_3)$, 1.130 (d, *J* = 7.5 Hz, 9H, $Si(CH(C\underline{H}_3)_3)$ ppm. **^{13}C NMR (100.6 MHz, $CDCl_3$)**: δ = 172.2 ($\underline{C}OOCH_3$), 155.2 ($\underline{C}OOC(CH_3)_3$), 141.7 (C-7a), 130.5 (C-2), 129.8 (C-6), 127.7 (C-3a), 120.4 (C-5), 119.5 (C-4), 113.9 (C-7), 112.4 (C-3), 79.9 ($O\underline{C}(CH_3)_2$), 54.2 ($NH\underline{C}HCH_2$), 52.3 (OCH_3), 28.5 ($OC(\underline{C}H_3)_3$), 28.2 ($NHCH\underline{C}H_2$), 18.2 ($Si(CH(\underline{C}H_3)_3)$, 12.9 ($Si(\underline{C}H(CH_3)_3)$ ppm.

Methyl (*S*)-3-(*tert*-butoxycarbonyl)-1,2,3-oxathiazolidine-4-carboxylate-2,2-dioxide (12)

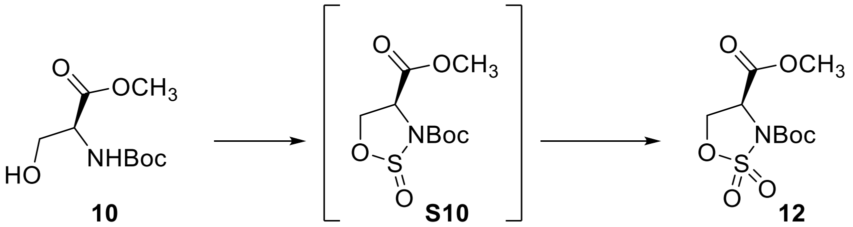

10 **S10** **12**

$SOCl_2$ (2.68 mL, 36.88 mmol) was added at –42 °C to a flame-dried Schlenk flask containing dry CH_2Cl_2 (33 mL), which was degassed by three freeze-pump-thaw cycles.

The flask was equipped with a dropping funnel and a solution of L-Boc-Ser-OMe **10** (5.39 g, 24.58 mmol) in dry CH_2Cl_2 (33 mL, degassed by three freeze-pump-thaw cycles) was added over 30 min, followed by dry pyridine (5.93 mL, 73.74 mmol) over 10 min. The solution, which turned opaque and yellow, was stirred for 15 min, slowly warmed to 23 °C over 1 h and stirred for 15 h. The mixture was cooled to 0 °C and quenched with ice (10 small chunks), stirred for 5 min, and filtered through celite. The solution was washed with sat. $NaHCO_3$ solution (2x33 mL) and brine (40 mL), dried over $MgSO_4$, filtered, and concentrated under reduced pressure. The resulting residue was dissolved in Et_2O, filtered through celite, and concentrated under reduced pressure, giving **S10** (5.65 g, ~87%) as a red oil, which was used in the next step without further purification. Sulfamidite **S10** (5.65 g, 21.29 mmol) was dissolved in CH_3CN (48 mL), and the solution was cooled to 0 °C. $RuCl_3{\cdot}H_2O$ (28.0 mg, 0.123 mmol) was added, followed by a solution of $NaIO_4$ (5.78 g, 27.03 mmol) in H_2O (48 mL). The brown suspension was stirred at 0 °C for 15 min, warmed to 23 °C, and stirred for 2 h. The mixture was diluted with Et_2O (100 mL) and brine (80 mL). The layers were separated, and the aqueous was extracted with EtOAc (4x80 mL). The combined organic layers were washed with sat. $NaHCO_3$ solution (2x90 mL) and brine (90 mL), dried over Na_2SO_4, filtered, and concentrated under reduced pressure. The resulting residue was recrystallized from a 2:1 Et_2O/CH_2Cl_2 mixture. After standing in the freezer overnight, the crystals were filtered and washed with an ice-cold mixture of pentane and Et_2O, followed by ice-cold Et_2O. The procedure was repeated on the filtrate after concentrating it. This provided **12** as pale-yellow crystals (4.72 g, 68% over two steps). **R_f**: 0.16 (petroleum ether/EtOAc = 2:1), **$[\alpha]^{20}_D$**: –32.9 (*c* 0.249, EtOAc). **^{1}H NMR (400 MHz, $CDCl_3$)**: δ = 4.81 (dd, *J* = 6.7, 1.9 Hz, 1H, $CH_2C\underline{H}N$), 4.77 (dd, *J* = 9.1, 6.7 Hz, 1H, $C\underline{H_2}CHN$), 4.69 (dd, *J* = 9.1, 1.9 Hz, 1H, $C\underline{H_2}CHN$), 3.86 (s, 3H, OCH_3), 1.56 (s, 9H, $OC(CH_3)_3$) ppm. **^{13}C NMR (100.6 MHz, $CDCl_3$)**: δ = 167.6 ($\underline{C}OOCH_3$), 148.2 ($\underline{C}OOC(CH_3)_3$), 86.5 ($O\underline{C}(CH_3)_3$), 67.6 ($\underline{C}H_2CHN$), 57.6 ($CH_2\underline{C}HN$), 53.8 (OCH_3), 28.0 ($OC(\underline{C}H_3)_3$) ppm. The NMR data are in line with those reported in the literature.[9]

(*S*)-N^{α}-(*tert*-Butoxycarbonyl)-6-chlorotryptophan methyl ester (13)

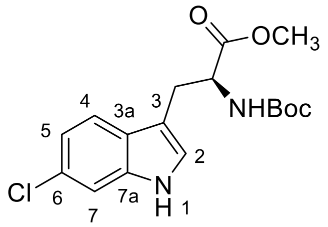

A flame-dried Schlenk flask was charged with 1H-6-chloroindole (323 mg, 2.13 mmol), which was dissolved in dry CH_2Cl_2 (7.1 mL). CuCl (182 mg, 1.85 mmol) was added as a single portion, the mixture was degassed by three freeze-pump-thaw cycles and cooled to 0 °C. MeMgCl (3.0 M in THF, 0.62 mL, 1.85 mmol) was added over 10 min. The suspension was stirred at 0 °C for 1 h and cooled to –20 °C. A similarly degassed solution of sulfamidate **12** (400 mg, 1.42 mmol) in dry CH_2Cl_2 (1.6 mL) was added over 20 min, the mixture was warmed to 23 °C, stirred for 2.5 h, and diluted with CH_2Cl_2 (14 mL). A 2 M citric acid solution (14 mL) was added dropwise at 0 °C, the mixture was warmed to 23 °C, stirred for 10 min, and filtered through celite. The layers were separated and the aqueous was extracted with CH_2Cl_2 (3x15 mL). The combined organic layers were washed with brine (45 mL), dried over Na_2SO_4, filtered, and concentrated under reduced pressure. Purification by flash column chromatography on silica gel, eluting with 2%, 4%, 13% and 20% EtOAc in cyclohexane, provided **13** as yellow crystals (186 mg, 37%). **R*f***: 0.14 (petroleum ether/EtOAc = 8:2). **M.p.**: 133-134 °C. **$[\alpha]^{20}_{D}$** : +43.4 (*c* 0.205, $CHCl_3$), lit[10] **$[\alpha]^{25}_{D}$**: –40 (*c* 0.20, $CHCl_3$) for the enantiomer. **MS ESI+ *m/z*, (%)**: 377/375 (32/100, $[M+Na]^+$), 321/319 (4/12, $[(M–isobutene)+Na]^+$), 299/297 (7/22, M–isobutene)+H]$^+$), 255/253 (13/43, $[(M–isobutene–CO_2)+H]^+$). **HRMS ESI+ *m/z*, ($[M+Na]^+$):** calcd. for $C_{17}H_{21}O_4N_2{}^{35}ClNa$: 375.1082, found: 375.1082. Calcd. for $C_{17}H_{21}O_4N2^{37}ClNa$: 377.1052, found: 377.1053. **IR**: ν (cm^{-1}) 3339, 2978, 2953, 1737, 1693, 1620, 1503, 1455, 1439, 1392, 1366, 1252, 1216, 1165, 1062, 1021, 908, 805. **^{1}H NMR (400 MHz, $CDCl_3$)**: δ = 8.07 (br s, 1H, NH indole), 7.45 (d, *J* = 8.5 Hz, 1H, H-4), 7.34 (d, *J* = 1.8 Hz, 1H, H-7), 7.08 (dd, *J* = 8.5, 1.8 Hz, 1H, H-5), 7.00 (d, *J* = 2.3 Hz, 1H, H-2), 5.06 (br d, *J* = 8.3 Hz, 1H, N<u>H</u>CHCH$_2$), 4.65-4.61 (m, 1H, NHC<u>H</u>CH$_2$), 3.67 (s, 3H, OCH_3), 3.27-3.24 (m, 2H, NHCHC<u>H</u>$_2$), 1.43 (s, 9H, $OC(CH_3)_3$) ppm. **^{13}C NMR (100.6 MHz, $CDCl_3$)**: δ = 172.7 (<u>C</u>OOCH$_3$), 155.3 (<u>C</u>OOC(CH$_3$)$_3$), 136.5 (C-7a), 128.6 (C-6), 126.8 (C-3a), 123.5 (C-2), 120.6 (C-5), 119.9 (C-4), 111.2 (C-7), 110.8 (C-3), 80.1 (O<u>C</u>(CH$_3$)$_3$), 54.3 (NH<u>C</u>HCH$_2$),

52.5 (OCH_3), 28.5 ($OC(\underline{C}H_3)_3$), 28.1 ($NHCH\underline{C}H_2$) ppm. The NMR data match those reported in the literature.[10]

(*S*)-*N*$^{\alpha}$-(*tert*-Butoxycarbonyl)-6-chlorotryptophan (5)

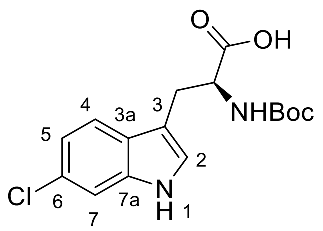

A 50 mL flask was charged with methyl ester **13** (586 mg, 1.66 mmol), which was dissolved in THF (14 mL). A solution of $LiOH{\cdot}H_2O$ (342 mg, 8.15 mmol) in H_2O (7 mL) was added, and the yellow suspension was stirred at 23 °C for 2 h. THF was evaporated under reduced pressure, the mixture was cooled to 0 °C, acidified with aq. citric acid solution (0.7 M) to pH 2-3, and extracted with EtOAc (3x60 mL). The combined organic layers were dried over $MgSO_4$, filtered, and concentrated under reduced pressure. Purification by flash chromatography on silica gel, eluting with 50% EtOAc in cyclohexane, gave **5** as a yellow solid (475 mg, 85%). **R_f**: 0.29 (petroleum ether/EtOAc = 1:1). **M.p.**: 130-132 °C. **$[\alpha]^{20}_{D}$**: +28.6 (*c* 0.252, EtOAc). **MS ESI+ *m/z*, (%)**: 703/701/699 (1/6/14, $[2M+Na]^+$), 363/361 (30/100, $[M+Na]^+$), 285/283 (1/5, $[(M–isobutene)+H]^+$), 241/239 (3/14, $[(M–CO_2–isobutene)+H]^+$). **HRMS ESI+ *m/z*, ($[M+Na]^+$):** calcd. for $C_{16}H_{19}O_4N_2{}^{35}ClNa$**:** 361.0926, found: 361.0926. Calcd. for $C_{16}H_{19}O_4N_2{}^{37}ClNa$: 363.0896, found: 363.0896. **IR**: ν (cm^{-1}): 3650-2300 (br), 3400, 3342, 2979, 2932, 1693, 1621, 1506, 1455, 1394, 1369, 1338, 1249, 1161, 1100, 1061, 1047, 938, 907, 850, 803. **^{1}H NMR (400 MHz, CD_3CN)**: δ = 9.26 (br s, 1H, NH indole), 7.53 (d, *J* = 8.5, Hz, 1H, H-4), 7.42 (d, *J* = 1.9 Hz, 1H, H-7), 7.12 (d, *J* = 2.6 Hz, 1H, H-2), 7.04 (dd, *J* = 8.5, 1.9 Hz, 1H, H-5), 5.47 (br d, *J* = 8.3 Hz, 1H, $N\underline{H}CHCH_2$), 4.42-4.36 (m, 1H, $NHC\underline{H}CH_2$), 3.25 (dd, *J* = 14.7, 5.2 Hz, 1H, $NHCHC\underline{H}_2$), 3.09 (dd, *J* = 14.7, 7.8 Hz, 1H, $NHCHC\underline{H}_2$), 1.35 (s, 9H, $OC(CH_3)_3$) ppm. The COOH signal is not observed in the ^{1}H NMR spectrum. **^{13}C NMR (100.6 MHz, CD_3CN)**: δ = 171.8 (COOH), 156.4 ($\underline{C}OOC(CH_3)_3$), 137.6 (C-7a), 127.8 (C-6), 127.3 (C-3a), 125.6 (C-2), 120.7 (C-4), 120.3 (C-5), 112.1 (C-7), 111.3 (C-3), 80.0 ($O\underline{C}(CH_3)_3$), 55.0 ($NH\underline{C}HCH_2$), 28.5 ($OC(\underline{C}H_3)_3$), 27.9 ($NHCH\underline{C}H_2$) ppm.

(*S*)-2-(3-Methylbut-2-en-1-yl)proline methyl ester (11)

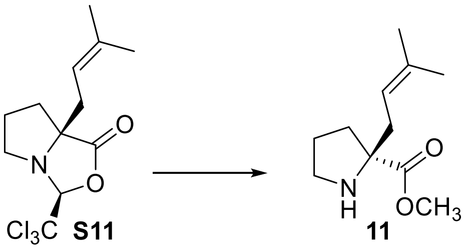

A flame-dried Schlenk flask was charged with oxazolidinone **S11** (1.00 g, 3.20 mol), which was prepared from D-proline according to the literature.[5,11,12] **S11** was dissolved in dry MeOH (5.5 mL), a solution of ammonia (7 M in MeOH, 4.6 mL, 32 mmol) was added, and the pale-yellow solution was stirred at 23 °C for 16 h. The volatiles were evaporated and the crude product was purified by flash chromatography on silica gel, eluting with 20% and 30% EtOAc in petroleum ether, giving **11** (450 mg, 71%), which contains 5 mol% of *N*-formylated methyl ester. $\mathbf{R}_{\boldsymbol{f}}$: 0.36 (EtOAc). $[\boldsymbol{\alpha}]^{20}_{\mathbf{D}}$: +53.6 (*c* 0.405, $CHCl_3$); lit.[5] $[\boldsymbol{\alpha}]^{20}_{\mathbf{D}}$: –59.8 (*c* 0.403, $CHCl_3$) for the enantiomer. **^{1}H NMR (400 MHz, $CDCl_3$)**: δ = 5.11-5.07 (m, 1H, $CH_2C\underline{H}=C(CH_3)_2$), 3.69 (s, 3H, OCH_3), 2.99-2.98 (m, 2H, $NHC\underline{H_2}CH_2CH_2$), 2.53 (ddquint, *J* = 14.1, 7.8, 1.0 Hz, 1H, $C\underline{H_2}CH=C(CH_3)_2$), 2.29 (ddquint, *J* = 14.1, 7.0, 1.0 Hz, 1H, $C\underline{H_2}CH=C(CH_3)_2$), 2.19-2.12 (m, 1H, $NHCH_2CH_2C\underline{H_2}$), 1.85-1.68 (m, 3H, $NHCH_2C\underline{H_2}CH_2$, $NHCH_2CH_2C\underline{H_2}$), 1.69 (q, *J* = 1.3 Hz, 3H, CH_3), 1.60 (s, 3H, CH_3) ppm. **^{13}C NMR (100.6 MHz, $CDCl_3$)**: δ = 177.6 (CO), 134.9 ($\underline{C}(CH_3)_2$), 119.3 ($CH_2\underline{C}H=C(CH_3)_2$, 69.8 (C Pro), 52.3 ($OCH_3$), 46.6 ($NH\underline{C}H_2CH_2CH_2$), 38.2 ($\underline{C}H_2CH=C(CH_3)_2$), 35.3 ($NHCH_2CH_2\underline{C}H_2$), 26.1 ($CH_3$), 25.1 ($NHCH_2\underline{C}H_2CH_2$), 18.1 ($CH_3$) ppm. The NMR data are in line with those reported in the literature.[5]

Methyl (*S*)-1-((*S*)-2-amino-3-(6-chloro-1H-indol-3-yl)propanoyl)-2-(3-methylbut-2-en-1-yl)pyrrolidine-2-carboxylate (14)

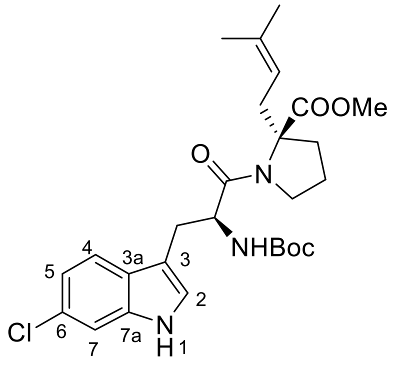

A flame-dried Schlenk flask was charged with tryptophan **5** (381 mg, 1.12 mmol), which was suspended in dry CH_2Cl_2 (8.8 mL). EEDQ (307 mg, 1.24 mmol) was added in a single portion, followed by proline methyl ester **11** (245 mg, 1.17 mmol, contains mol 5% of *N*-formylated proline methyl ester) as a solution in dry CH_2Cl_2 (2.9 mL). The mixture was stirred at 23 °C for 17 h and concentrated under reduced pressure. Purification of the residue by flash chromatography on silica gel, eluting with 5%, 10%, 15%, 20%, 25% and 30% EtOAc in cyclohexane, provided **14** as a colorless solid, which contains 4 mol% of *N*-formylated proline ester (442 mg, 76%). **R_f**: 0.15 (cyclohexane/EtOAc = 2:1). **M.p.** 66-68 °C. **$[\alpha]^{20}_D$**: –2.9 (*c* 0.248, EtOAc). **MS ESI+ *m/z*, (%)**: 1061/1059/1057 (1/3/6, $[2M+Na]^+$), 542/540 (30/100, $[M+Na]^+$), 520/518 (3/15, $[M+H]^+$), 464/462 (2/9, [(M–isobutene)+H^+), 420/418 (4/16, $[(M–CO_2–isobutene)+H]^+$). **HRMS ESI+ *m/z***, ($[M+H]^+$): calcd. for $C_{27}H_{37}{}^{35}ClN_3O_5$: 518.2416, found: 518.2416. Calcd. for $C_{27}H_{37}{}^{37}ClN_3O_5$: 520.2387, found: 520.2385. **IR**: ν (cm^{-1}) 3309, 2976, 2930, 1736, 1707, 1637, 1498, 1435, 1392, 1366, 1337, 1142, 1166, 1127, 1108, 907, 850, 803, 736. **^{1}H NMR (400 MHz, $CDCl_3$)**: δ = 8.10 (br s, 1H, NH indole), 7.60 (d, *J* = 8.5 Hz, 1H, H-4), 7.33 (d, *J* = 1.8 Hz, 1H, H-7), 7.09 (dd, *J* = 8.5, 1.8 Hz, 1H, H-5), 7.04 (d, *J* = 2.3 Hz, 1H, H-2), 5.28 (d, *J* = 9.0 Hz, 1H, $N\underline{H}CHCH_2$), 4.85-4.79 (m, 1H, $NHC\underline{H}CH_2$), 4.49-4.40 (m, 1H, $CH_2C\underline{H}=C(CH_3)_2$), 3.84 (dd, *J* = 8.3, 6.8 Hz, 1H, $NC\underline{H}_2CH_2CH_2$), 3.68 (s, 3H, OCH_3), 3.16 (dd, *J* = 14.3, 8.1 Hz, 1H, $NHCHC\underline{H}_2$), 3.07-3.01 (m, 2H, $NHCHC\underline{H}_2$, $NC\underline{H}_2CH_2CH_2$), 2.72 (dd, *J* = 14.9, 6.0 Hz, 1H, $C\underline{H}_2CH=C(CH_3)_2$), 2.63 (dd, *J* = 14.9, 8.9 Hz, 1H, $C\underline{H}_2CH=C(CH_3)_2$), 1.93-1.82 (m, 3H, $NCH_2CH_2C\underline{H}_2$, $NCH_2C\underline{H}_2CH_2$), 1.72-1.67 (m, 1H, $NCH_2C\underline{H}_2CH_2$), 1.58 (s, 3H, CH_3), 1.56 (s, 3H, CH_3), 1.41 (s, 9H, $OC(CH_3)_3$) ppm. **^{13}C NMR (100.6 MHz, $CDCl_3$)**: δ = 174.6 ($\underline{C}OOCH_3$), 170.7 (CON), 155.8 ($\underline{C}OOC(CH_3)_3$), 136.8 (C-7a), 135.6 ($CH_2CH=\underline{C}(CH_3)_2$), 128.5 (C-6), 126.8 (C-3a), 124.1

(C-2), 120.9 (C-5), 120.2 (C-4), 119.2 ($CH_2\underline{C}H=C(CH_3)_2$), 111.6 (C-7), 111.5 (C-3), 80.1 ($O\underline{C}(CH_3)_3$), 69.4 (C Pro), 52.9 ($NH\underline{C}HCH_2$), 52.7 (OCH_3), 49.3 ($N\underline{C}H_2CH_2CH_2$), 35.2 ($NCH_2CH_2\underline{C}H_2$), 31.9 ($\underline{C}H_2CH=C(CH_3)_2$), 29.5 ($NHCH\underline{C}H_2$), 28.8 ($OC(\underline{C}H_3)_3$), 26.5 ($CH_3$), 24.3 ($NCH_2\underline{C}H_2CH_2$), 18.5 ($CH_3$) ppm.

(3*S*,8*aS*)-3-((6-Chloro-1H-indol-3-yl)methyl)-8a-(3-methylbut-2-en-1-yl)hexahydropyrrolo[1,2-a]pyrazine-1,4-dione (8a)

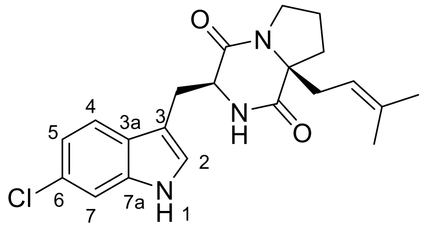

A flame-dried Schlenk flask was charged with SiO_2 (2.22 g, 6 weight equiv.) and dipeptide **14** (370 mg, 0.69 mmol) dissolved in dry CH_2Cl_2 (15 mL) was added. The solvent was carefully evaporated under reduced pressure, and the mixture was kept in high vacuum for 30 min. The flask was evacuated and filled with nitrogen three consecutive times, equipped with a reflux condenser, and placed in a silicone oil bath kept at 190 °C. The mixture was vigorously stirred at this temperature for 2 h and cooled to 23 °C. The silica gel was transferred to a frit, washed with acetone and the solvent was evaporated under reduced pressure, giving crude **8a**. Purification by flash chromatography on silica gel, eluting with 50%, 75% and 80% EtOAc in cyclohexane provided **8a** as a colorless solid (212 mg, 80%). **R*f***: 0.24 (EtOAc/petroleum ether = 3:1). **M.p.**: 142-143 °C. **$[\alpha]^{20}_D$**: –92.3 (*c* 0.247, EtOAc). **MS ESI+ *m/z*, (%)**: 797/795/793 (3/14/25, $[2M+Na]^+$), 410/408 (29/100, $[M+Na]^+$), 388/386 (7/34, $[M+H]^+$). **HRMS ESI+ *m/z*, (%)** ($[M+Na]^+$): calcd. for $C_{21}H_{24}{}^{35}ClN_3O_2Na$: 408.1449, found: 408.1450. Calcd. for $C_{21}H_{24}{}^{37}ClN_3O_2Na$: 410.1420, found: 410.1418. ($[M+H]^+$): calcd. for $C_{21}H_{25}{}^{35}ClN_3O_2$: 386.1630, found: 386.1630. Calcd. for $C_{21}H_{25}{}^{37}ClN_3O_2$: 388.1601, found: 388.1602. **IR**: ν (cm^{-1}) 3258, 2965, 2920, 1671, 1623, 1451, 1378, 1331, 1305, 1262, 1236, 1104, 1067, 908, 847, 803. **^{1}H NMR (400 MHz, $CDCl_3$)**: δ = 8.31 (br s, 1H, NH indole), 7.54 (d, *J* = 8.4 Hz, 1H, H-4), 7.39 (d, *J* = 1.8 Hz, 1H, H-7), 7.12 (dd, *J* = 8.5, 1.8 Hz, 1H, H-5), 7.05 (d, *J* = 2.4 Hz, 1H, H-2), 5.67 (br s, 1H, $N\underline{H}CHCH_2$), 5.22-5.17 (m, 1H, $CH_2C\underline{H}=C(CH_3)_2$), 4.16 (dt, *J* = 11.3, 3.0 Hz, 1H, $NHC\underline{H}CH_2$), 4.07-4.00 (m, 1H, $NC\underline{H}_2CH_2CH_2$), 3.58 (dd, *J* = 14.3, 3.0 Hz, 1H, $NHCHC\underline{H}_2$), 3.50-3.43 (m, 1H, $NC\underline{H}_2CH_2CH_2$), 2.98 (dd, *J* = 14.3, 11.3 Hz, 1H,

NHCHC$\underline{H}_2$), 2.57 (dd, J = 14.3, 8.9 Hz, 1H, C$\underline{H}_2$CH=C(CH$_3$)$_2$), 2.35 (dd, J = 14.3, 7.6 Hz, 1H, C$\underline{H}_2$CH=C(CH$_3$)$_2$), 2.23-2.20 (m, 1H, NCH$_2$CH$_2$C$\underline{H}_2$), 2.07-1.95 (m, 3H, NCH$_2$CH$_2$C$\underline{H}_2$, NCH$_2$C$\underline{H}_2$CH$_2$), 1.80 (s, 3H, CH$_3$), 1.67 (d, J = 1.0 Hz, 3H, CH$_3$) ppm. **^{13}C NMR (100.6 MHz, CDCl$_3$)**: δ = 170.1 (CONH), 164.8 (CON), 137.4 (CH$_2$CH=$\underline{C}$(CH$_3$)$_2$), 137.0 (C-7a), 128.8 (C-6), 125.5 (C-3a), 123.9 (C-2), 121.0 (C-5), 119.8 (C-4), 118.2 (CH$_2$$\underline{C}$H=C(CH$_3$)$_2$), 111.5 (C-7), 110.9 (C-3), 67.9 (C Pro), 58.1 (NH$\underline{C}$HCH$_2$), 44.9 (N$\underline{C}$H$_2$CH$_2$CH$_2$), 36.3 ($\underline{C}$H$_2$CH=C(CH$_3$)$_2$), 35.3 (NCH$_2$CH$_2$$\underline{C}H_2$), 31.8 (NHCH$\underline{C}H_2$), 26.5 (CH$_3$), 19.8 (NCH$_2$$\underline{C}H_2CH_2$), 18.2 (CH$_3$) ppm.

(3*S*,8a*S*)-3-((6-Chloro-1-(methoxymethyl)-1H-indol-3-yl)methyl)-2-(methoxymethyl)-8a-(3-methylbut-2-en-1-yl)hexahydropyrrolo[1,2-a]pyrazine-1,4-dione (8b)

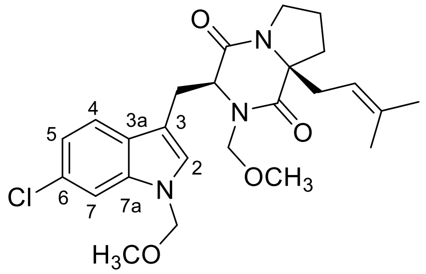

A flame-dried Schlenk tube was charged with diketopiperazine **8a** (160 mg, 0.415 mmol), which was dissolved in dry THF (4 mL). The solution was degassed by three freeze-pump-thaw cycles and cooled to 0 °C. NaH (60% in mineral oil, 46 mg, 1.161 mmol) and KHMDS (1.0 M in tetrahydrofuran, 0.116 mL, 0.116 mmol) were added. The resulting mixture was stirred at 0 °C for 1 h and MOMBr (0.12 mL, 1.66 mmol) was dropwise added. The reaction mixture was warmed to 23 °C and stirred for 2 h. Sat. NH$_4$Cl solution (1.5 mL) and EtOAc (7 mL) were added at 0 °C and the mixture was warmed to 23 °C. The layers were separated and the aqueous was extracted with EtOAc (2x7 mL) and Et$_2$O (2x7 mL). The combined organic layers were dried over Na$_2$SO$_4$, filtered, and concentrated under reduced pressure. Purification of the residue by flash chromatography on silica gel, eluting with 50% and 66% EtOAc in cyclohexane provided **8b** as a colorless oil (162 mg, 83%). **R*f***: 0.21 (petroleum ether/EtOAc = 1:3). **[α]$^{20}_{D}$**: +20.2 (*c* 0.247, EtOAc). **MS ESI+ *m/z*, (%)**: 973/971/969 (4/11/20, [2M+Na]$^+$), 498/496 (29/100, [M+Na]$^+$). **HRMS ESI+ *m/z*, (%)** ([M+Na]$^+$): calcd. for C$_{25}$H$_{32}$^{35}ClN$_3$O$_4$Na: 496.1974, found: 496.1970. Calcd. for C$_{25}$H$_{32}$^{37}ClN$_3$O$_4$Na: 498.1950, found: 498.1942. ([M+H]$^+$): calcd. for C$_{25}$H$_{33}$^{35}ClN$_3$O$_4$: 474.2154, found: 474.2152. Calcd. for C$_{25}$H$_{33}$^{37}ClN$_3$O$_4$:

476.2125, found: 476.2122. **IR**: ν (cm^{-1}) 2927, 1655, 1552, 1430, 1387, 1360, 1326, 1281, 1236, 1211, 1163, 1132, 1092, 1045, 913, 807, 647. **^{1}H NMR (400 MHz, CD_3CN)**: δ = 7.58 (dd, *J* = 8.5, 0.5 Hz, 1H, H-4), 7.50 (dd, *J* = 1.8, 0.5 Hz, 1H, H-7), 7.19 (s, 1H, H-2), 7.12 (dd, *J* = 8.5, 1.8 Hz, 1H, H-5), 5.38 (d, *J* = 11.2 Hz, 1H, OCH_2N indole), 5.34 (d, *J* = 11.2 Hz, 1H, OCH_2N indole), 4.96 (d, *J* = 10.0 Hz, 1H, OCH_2NCO), 4.85-4.80 (m, 1H, $CH_2C\underline{H}=C(CH_3)_2$), 4.53 (d, *J* = 10.0 Hz, 1H, OCH_2NCO), 4.31 (t, *J* = 5.3 Hz, 1H, $NC\underline{H}CH_2$), 3.82-3.75 (m, 1H, $NC\underline{H}_2CH_2CH_2$), 3.35 (dd, *J* = 5.3, 0.6 Hz, 2H, $NCHC\underline{H}_2$), 3.31-3.23 (m, 1H, $NC\underline{H}_2CH_2CH_2$), 3.22 (s, 3H, OCH_3), 3.17 (s, 3H, OCH_3), 2.06-1.99 (m, 1H, $NCH_2CH_2C\underline{H}_2$), 1.90-1.70 (m, 5H, $NCH_2CH_2C\underline{H}_2$, $NCH_2C\underline{H}_2CH_2$, $C\underline{H}_2CH=C(CH_3)_2$, 1.64 (d, *J* = 1.3 Hz, 3H, CH_3), 1.47 (d, *J* = 1.5 Hz, 3H, CH_3) ppm. **^{13}C NMR (100.6 MHz, CD_3CN)**: δ = 171.2 ($\underline{C}ONCH_2O$), 165.6 ($\underline{C}OCHCH_2$), 138.0 (C-7a), 135.8 ($CH_2CH=\underline{C}(CH_3)_2$), 129.9 (C-2), 128.6 (C-6), 128.3 (C-3a), 121.6 (C-5), 121.2 (C-4), 119.4 ($CH_2\underline{C}H=C(CH_3)_2$), 112.3 (C-3), 111.1 (C-7), 77.9 (OCH_2N indole), 76.0 ($O\underline{C}H_2NCO$), 68.4 (C Pro), 62.1 ($N\underline{C}HCH_2$), 56.7 ($\underline{C}H_3OCH_2NCO$), 56.2 ($\underline{C}H_3OCH_2N$ indole), 46.0 ($N\underline{C}H_2CH_2CH_2$), 37.6 ($\underline{C}H_2CH=C(CH_3)_2$), 35.5 ($NCH_2CH_2\underline{C}H_2$), 30.0 ($NCH\underline{C}H_2$), 26.0 ($CH_3$), 20.4 ($NCH_2\underline{C}H_2CH_2$), 17.9 ($CH_3$) ppm.

(6*S*,7*R*,8a*S*)-6-((6-Chloro-1-(methoxymethyl)-1H-indol-3-yl)methyl)-10-(methoxymethyl)-7-(prop-1-en-2-yl)tetrahydro-1*H*-6,8a-(epiminomethano)indolizine-5,9(6H)-dione (15)

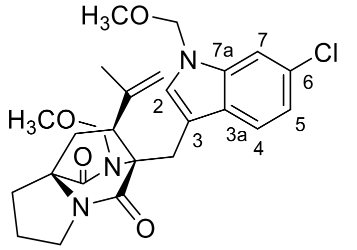

Condition A: A flame-dried Schlenk tube was charged with diketopiperazine **8b** (61 mg, 0.129 mmol), which was dissolved in dry DME (3.6 mL). The solution was degassed by three freeze-pump-thaw cycles and cooled to –78 °C. NaHMDS (1 M in THF, 0.284 mL, 0.284 mmol) was dropwise added and the resulting yellow solution was stirred for 30 min. The Schlenk tube was equipped with a condenser, placed in a pre-heated 100 °C silicone oil bath, and Cp_2FePF_6 (141 mg, 0.426 mmol) was added as a single portion. The reaction mixture was refluxed for 45 min, cooled to 23 °C and the solvent was evaporated under

reduced pressure. The resulting residue was suspended in benzene and filtered through a pad of silica gel, which was eluted with cyclohexane and subsequently EtOAc. The solvents were removed under reduced pressure. Threefold purification of the resulting residue by flash chromatography on silica gel, eluting with 20%, 33% and 50% EtOAc in cyclohexane provided **15** as a colorless solid (54 mg, 87%).

Condition B: A flame-dried Schlenk tube was charged with diketopiperazine **8b** (39 mg, 0.082 mmol), which was dissolved in dry DME (1.7 mL). The solution was degassed by three freeze-pump-thaw cycles and cooled to –78 °C. *n*-BuLi (1.6 M in hexane, 0.082 mL, 0.131 mmol) was dropwise added, the resulting pale yellow solution was stirred for 1.5 h and warmed to –40 °C over 30 min. TEMPO (16 mg, 0.103 mmol) was added as a single portion, followed by $Cp_2Fe^+PF_6^-$ (43 mg, 0.131 mmol) in small portions until the blue color persisted. The mixture was stirred for 15 min, the tube was equipped with a reflux condenser and placed into a pre-heated 100 °C silicone oil bath. The reaction mixture was stirred for 1.5 h, cooled to 23 °C and the solvent was evaporated under reduced pressure. The resulting residue was dissolved in EtOAc and filtered through a pad of silica gel, which was eluted with EtOAc. Concentration under reduced pressure and purification of the crude product by flash chromatography on silica gel, eluting with 33% and 50% EtOAc in cyclohexane provided **15** as a colorless solid (16 mg, 44%).

R_f: 0.30 (cyclohexane/EtOAc = 1:3). **M.p.**: 73-75 °C. **$[\alpha]^{20}_D$**: +8.2 (*c* 0.25, EtOAc). **MS ESI+ *m/z*, (%)**: 496/494 (27/100, $[M+Na]^+$), 451/449 (1/9, $[(M–CH_3OCH_2)+Na]^+$). **HRMS ESI+ *m/z*, (%)** ($[M+Na]^+$): calcd. for $C_{25}H_{30}{}^{35}ClN_3O_4Na$: 494.1817, found: 494.1814. calcd. for $C_{25}H_{30}{}^{37}ClN_3O_4Na$: 496.1787, found: 496.1785. **IR**: ν (cm^{-1}) 2926, 1679, 1637, 1468, 1379, 1320, 1131, 1087, 843. **^1H NMR (400 MHz, CD_3CN)**: δ = 7.57 (d, *J* = 8.5 Hz, 1H, H-4), 7.52 (d, *J* = 1.9 Hz, 1H, H-7), 7.26 (s, 1H, H-2), 7.13 (dd, *J* = 8.5, 1.9 Hz, 1H, H-5), 5.40 (d, *J* = 11.1 Hz, 1H, OCH_2N indole), 5.35 (d, *J* = 11.1 Hz, 1H, OCH_2N indole), 4.74 (d, *J* = 10.6 Hz, 1H, OCH_2NCO), 4.73 (br s, 1H, $C=CH_2$), 4.66 (d, *J* = 10.6 Hz, 1H, OCH_2NCO), 4.63 (br s, 1H, $C=CH_2$), 3.54-3.50 (m, 2H, $NC\underline{H}_2CH_2CH_2$), 3.42 (dd, *J* = 17.5, 1.5 Hz, 1H, CH_2 Trp), 3.38-3.30 (m, 2H, CH bridge, CH_2 Trp), 3.17 (s, 3H, $C\underline{H}_3OCH_2N$ indole), 3.06 (s, 3H, $C\underline{H}_3OCH_2NCO$), 2.69 (dd, *J* = 12.3, 6.5 Hz, 1H, $NCH_2CH_2C\underline{H}_2$), 2.26 (dd, *J* = 13.6, 10.4 Hz, 1H, CH_2 bridge), 2.10-2.04 (m, 1H, $NCH_2C\underline{H}_2CH_2$), 1.98-1.86 (m, 3H, CH_2 bridge, $NCH_2CH_2C\underline{H}_2$, $NCH_2C\underline{H}_2CH_2$), 1.59 (s, 3H, CH_3) ppm. **^{13}C NMR (100.6 MHz, CD_3CN)**: δ = 175.3 ($\underline{C}ONCH_2O$), 168.7 ($\underline{C}ONCH_2CH_2$), 144.7 ($\underline{C}=CH_2$), 137.2 (C-7a), 129.2 (C-2), 129.0 (C-3a), 128.4 (C-6),

120.9 (C-5), 120.6 (C-4), 116.1 (C=$\underline{C}H_2$), 111.2 (C-3), 110.8 (C-7), 77.8 (OCH_2N indole), 74.5 ($O\underline{C}H_2NCO$), 68.6 (C bridgehead Pro), 66.9 (C bridgehead Trp), 56.6 ($\underline{C}H_3OCH_2NCO$), 56.0 ($\underline{C}H_3OCH_2N$ indole), 51.5 (CH bridge), 44.9 ($N\underline{C}H_2CH_2CH_2$), 36.7 ($CH_2$ bridge), 30.1 ($NCH_2CH_2\underline{C}H_2$), 24.7 ($NCH_2\underline{C}H_2CH_2$), 24.4 ($CH_2$ Trp), 19.3 (CH_3) ppm.

(6*S*,7*S*,8a*S*)-6-((6-Chloro-1-(hydroxymethyl)-1H-indol-3-yl)methyl)-7-(prop-1-en-2-yl)tetrahydro-1H-6,8a-(epiminomethano)indolizine-5,9-(6H)-dione (16)

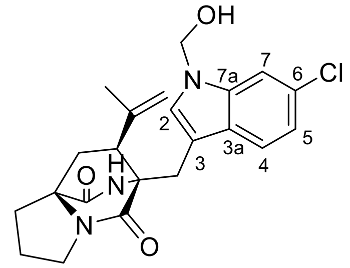

Bridged diketopiperazine **15** (54 mg, 0.114 mmol) was dissolved in a mixture of dry CH_2Cl_2 and dry CH_3CN (1:1 v/v, 2 mL). 2,2'-Bipyridine (107 mg, 0.686 mmol) was added at 0 °C, followed by dropwise addition of TESOTf (0.104 mL, 0.456 mmol). The resulting mixture was stirred at 0 °C for 4 h and H_2O (0.7 mL) was added followed by EtOAc (2 mL). The mixture was warmed to 23 °C, transferred to a 25 mL Schlenk flask and diluted with H_2O (0.8 mL) and EtOAc (3 mL). Sat. $NaHCO_3$ solution (1.3 mL) was added, and the biphasic solution was stirred at 23 °C for 16 h. Additional sat. $NaHCO_3$ solution was added after 16 h (0.7 mL) and after 20 h (0.1 mL). After stirring for 1.5 additional hours, the layers were separated and the aqueous was extracted with EtOAc (5x8 mL). The combined organic layers were dried over $MgSO_4$, filtered and concentrated under reduced pressure. Twofold purification by flash chromatography on silica gel eluting with 10% and 80% EtOAc in cyclohexane to remove bipyridine provided a mixture of **16** and N,N^a-bis-hemiaminal in 4:1 ratio as a colorless solid (78 mg). The mixture was dissolved in EtOAc (3 mL) and H_2O (0.3 mL) was added, followed by sat. $NaHCO_3$ solution (0.5 mL). The biphasic solution was stirred at 23 °C under N_2 atmosphere for 3 h. Additional sat. $NaHCO_3$ solution was added after 3 h (0.1 mL) and after 6 h (0.1 mL). The mixture was stirred for an additional hour, the layers were separated and the aqueous was extracted with EtOAc (5x8 mL). The combined organic layers were dried over $MgSO_4$, filtered and concentrated under reduced pressure. Twofold purification of the residue by flash chromatography on silica gel, eluting with 50% and 80% EtOAc in cyclohexane provided

16 as a colorless solid (45 mg), which needed to be re-purified. Twofold trituration with pentane, followed by flash chromatography on silica gel eluting with 50% and 80% EtOAc in pentane, and threefold trituration with pentane provided **16** (32 mg, 68%). **R*f***: 0.27 (pentane/EtOAc = 1:4). **M.p.**: 75-77 °C. **[α]^{20}D**: +9.5 (*c* 0.18, EtOAc). **MS ESI+ *m/z*, (%)**: 853/851/849 (2/4/10, $[2M+Na]^+$), 438/436 (29/100, $[M+Na]^+$), 398/396 (26/87, $[(M–OH^-)]^+$. **HRMS ESI+ *m/z*, (%)** ($[M+Na]^+$): calcd. for $C_{22}H_{24}O_3N_3{}^{35}ClNa$: 436.1398, found: 436.1401. calcd. for $C_{22}H_{24}O_3N_3{}^{37}ClNa$: 438.1369, found: 438.1371. **IR**: ν (cm^{-1}): 3479, 3282, 2960, 2924, 1687, 1614, 1467, 1395, 1363, 1260, 1173, 1035, 903, 852, 804. **^{1}H NMR (400 MHz, CD_3CN)**: δ = 7.65 (dd, *J* = 8.5, 0.5 Hz, 1H, H-4), 7.55 (dd, *J* = 1.9, 0.5 Hz, 1H, H-7), 7.30 (s, 1H, H-2), 7.12 (dd, *J* = 8.5, 1.9 Hz, 1H, H-5), 6.11 (br s, 1H, NH), 5.49 (d, *J* = 7.4 Hz, 2H, C<u>H</u>$_2$OH), 4.94-4.93 (m, 2H, C=CH_2), 4.43 (t, *J* = 7.4 Hz, 1H, CH_2O<u>H</u>), 3.49-3.43 (m, 2H, NC<u>H</u>$_2$$CH_2$$CH_2$), 3.40 (dd, *J* = 15.7, 1.0 Hz, 1H, CH_2 Trp), 3.04-3.02 (m, 1H, CH bridge), 3.03 (d, *J* = 15.7 Hz, 1H, CH_2 Trp), 2.54 (ddd, *J* = 13.1, 7.1, 6.0 Hz, 1H, N$CH_2$$CH_2$C<u>H</u>$_2$), 2.26-2.19 (m, 1H, CH_2 bridge), 2.03-1.99 (m, 1H, NCH_2C<u>H</u>$_2$$CH_2$), 1.90-1.78 (m, 3H, CH_2 bridge, N$CH_2$$CH_2$C<u>H</u>$_2$, N$CH_2$C<u>H</u>$_2$$CH_2$), 1.69 (dd, *J* = 1.8, 0.4 Hz, 3H, CH_3) ppm. **^{13}C NMR (126 MHz, CD_3CN)**: δ = 173.6 (CONH), 169.3 (<u>C</u>ONCH_2), 144.9 (<u>C</u>=CH_2), 137.1 (C-7a), 130.0 (C-2), 129.2 (C-6), 128.3 (C-3a), 121.1 (C-5), 120.0 (C-4), 116.0 (C=<u>C</u>H$_2$), 111.1 (C-7), 109.8 (C-3), 69.9 (CH_2OH), 67.4 (C bridgehead Pro), 64.7 (C bridgehead Trp), 53.4 (CH bridge), 44.7 (N<u>C</u>H$_2$$CH_2$$CH_2$), 37.3 ($CH_2$ bridge), 29.6 (N$CH_2$$CH_2$<u>C</u>H$_2$), 25.3 ($CH_2$ Trp), 25.0 (NCH_2<u>C</u>H$_2$$CH_2$), 19.7 ($CH_3$) ppm. **^{1}H NMR (500 MHz, CD_3OD)**: δ = 7.58 (dd, *J* = 8.5, 0.5 Hz, 1H, H-4), 7.55 (dd, *J* = 1.9, 0.5 Hz, 1H, H-7), 7.31 (s, 1H, H-2), 7.09 (dd, *J* = 8.5, 1.9 Hz, 1H, H-5), 5.53 (s, 2H, C<u>H</u>$_2$OH), 4.93-4.88 (m, 2H, C=CH_2), 3.55-3.52 (m, 2H, NC<u>H</u>$_2$$CH_2$$CH_2$), 3.39 (dd, *J* = 15.9, 1.1 Hz, 1H, CH_2 Trp), 3.15 (dd, *J* = 15.9, 0.7 Hz, 1H, CH_2 Trp), 3.08 (dd, 10.3, 5.5 Hz, CH bridge), 2.64 (ddd, 13.0, 7.0, 5.8 Hz, 1H, N$CH_2$$CH_2$C<u>H</u>$_2$), 2.27 (dd, *J* = 13.6, 10.3 Hz, 1H, CH_2 bridge), 2.12-2.07 (m, 1H, NCH_2C<u>H</u>$_2$$CH_2$), 1.99-1.93 (m, 1H, N$CH_2$C<u>H</u>$_2$$CH_2$), 1.91-1.85 (m, 2H, CH_2 bridge, N$CH_2$$CH_2$C<u>H</u>$_2$), 1.68 (dd, *J* = 1.3, 0.8 Hz, 3H, CH_3) ppm. **^{13}C NMR (126 MHz, CD_3OD)**: δ = 175.3 (CONH), 170.9 (<u>C</u>ONCH_2), 144.8 (<u>C</u>=CH_2), 137.6 (C-7a), 129.61 (C-6), 129.55 (C-2), 129.1 (C-3a), 121.4 (C-5), 120.8 (C-4), 116.4 (C=<u>C</u>H$_2$), 111.1 (C-7), 109.9 (C-3), 70.1 (CH_2OH), 68.2 (C Pro), 65.4 (C bridgehead), 53.2 (CH bridge), 45.3 (N<u>C</u>H$_2$$CH_2$$CH_2$), 37.5 ($CH_2$ bridge), 29.9 (N$CH_2$$CH_2$<u>C</u>H$_2$), 25.5 ($CH_2$ Trp), 25.3 (NCH_2<u>C</u>H$_2$$CH_2$), 19.9 ($CH_3$) ppm.

(5a*S*,12a*S*,13a*S*)-9-Chloro-12,12-dimethyl-2,3,11,12,12a,13-hexahydro-1H,5H,6H-5a,13a-(epiminomethano)indolizino[7,6-b]carbazole-5,14-dione (17)

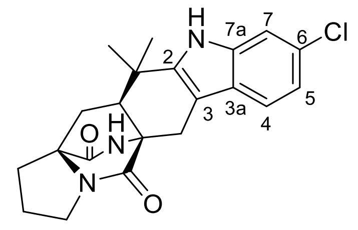

A Schlenk tube was charged with diketopiperazine **16** (10 mg, 0.024 mmol), which was dissolved in DME (1 mL) and conc. HCl solution (6 drops) was added. The tube was sealed, placed into a pre-heated 65 °C silicone oil bath, and the suspension was stirred for 4.5 h. HFIP (1 drop) was added, the mixture was stirred at 65 °C for an additional hour and cooled to 0 °C. EtOAc (1 mL) was added, followed by sat. NaOH solution (15 drops). The supernatant was carefully decanted from the precipitate into a beaker and the solid was thoroughly washed with EtOAc. The solution was dried over $MgSO_4$ for 30 minutes, filtered and concentrated under reduced pressure, providing a mixture of **17** and olefin **S7** in 4:1 molar ratio (**17**: 52%, **S7**: 13%) as a colorless solid. **R*f***: 0.24 (pentane/EtOAc = 1:4). **$[\alpha]^{20}_{D}$**: +61.8 (*c* 0.033, MeOH), lit.[13] **$[\alpha]^{19}_{D}$**: –122 (*c* 1.0, MeOH) for the enantiomer. **MS ESI+ *m/z*, (%)**: 408/406 (13/85, $[M+Na]^+$), 386/384 (23/100, $[M+H]^+$). **HRMS ESI+ *m/z*, (%)** ($[M+H]^+$): calcd. for $C_{21}H_{23}O_2N_3{}^{35}Cl$: 384.1473, found: 384.1474. calcd. for $C_{21}H_{23}O_2N_3{}^{37}Cl$: 386.1444, found: 386.1444. ($[M+Na]^+$): calcd. for $C_{21}H_{22}O_2N_3{}^{35}ClNa$: 406.1293, found: 406.1293. calcd. for $C_{21}H_{22}O_2N_3{}^{37}ClNa$: 408.1263, found: 408.1264. **IR**: ν (cm^{-1}): 3498, 2924, 2854, 1687, 1457, 1383, 1257, 1231, 1174, 846, 766, 643. **^{1}H NMR (600 MHz, CD_3CN)**: δ = 9.27 (br s, 1H, NH indole), 7.43 (dd, *J* = 8.4, 0.7 Hz, 1H, H-4), 7.33 (d, *J* = 1.9 Hz, 1H, H-7), 7.03 (dd, *J* = 8.4, 1.8 Hz, 1H, H-5), 6.83 (br s, 1H, NH amide), 3.55 (d, *J* = 15.4 Hz, 1H, CH_2 Trp), 3.40 (ddd, *J* = 11.3, 7.2, 5.7 Hz, 1H, $N\underline{C}\underline{H}_2CH_2CH_2$), 3.31 (dt, *J* = 11.3, 6.2 Hz, 1H, $NC\underline{H}_2CH_2CH_2$), 2.68 (d, *J* = 15.4 Hz, 1H, CH_2 Trp), 2.63-2.61 (m, 1H, CH bridge), 2.60-2.58 (m, 1H, $NCH_2CH_2C\underline{H}_2$), 2.17-2.13 (m, 1H, CH_2 bridge), 2.04-2.00 (m, 2H, CH_2 bridge, $NCH_2C\underline{H}_2CH_2$), 1.92-1.85 (m, 2H, $NCH_2CH_2C\underline{H}_2$, $NCH_2C\underline{H}_2CH_2$), 1.28 (s, 3H, CH_3), 1.04 (s, 3H CH_3) ppm. **^{13}C NMR (151 MHz, CD_3CN)**: δ = 174.2 (CONH), 169.6 (CON), 142.7 (C-2), 137.9 (C-7a), 127.4 (C-6), 126.5 (C-3a), 120.1 (C-5), 119.8 (C-4), 111.5 (C-7), 105.3 (C-3), 67.4 (C bridgehead Pro), 61.1 (C bridgehead Trp), 50.0 (CH bridge), 44.6 ($N\underline{C}H_2CH_2CH_2$), 35.7 ($\underline{C}(CH_3)_2$),

31.4 (CH_2 bridge), 29.8 ($NCH_2CH_2\underline{C}H_2$), 28.4 ($CH_3$), 25.1 ($NCH_2\underline{C}H_2CH_2$), 24.9 ($CH_2$ Trp), 22.2 (CH_3) ppm.

(5a*S*,12a*S*,13a*S*)-9-Chloro-12,12-dimethyl-2,3,11,12,12a,13-hexahydro-1H,5H,6H-5a,13a-(epiminomethano)indolizino[7,6-b]carbazol-14-one (malbrancheamide B) (1)

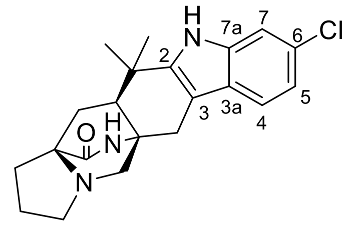

A mixture of diketopiperazine **17** and olefin **S7** in a 4:1 molar ratio (4 mg, 0.01 mmol) was suspended in toluene (2.5 mL) and the mixture was degassed by three freeze-pump-thaw cycles. DIBAL-H (1 M in toluene, 0.25 mL, 0.25 mmol) was dropwise added. The reaction mixture was stirred at 23 °C for 5.5 h, and Na_2SO_4 (4 micro spoons) and H_2O (2 drops) were added. The suspension was stirred for 10 min and filtered through a pad of celite, which was eluted with EtOAc and subsequently with MeOH. The solvents were evaporated and purification of the residue by flash column chromatography on silica gel, eluting with 2% MeOH in CH_2Cl_2, provided **1** as a colorless thin film (1.1 mg, 36%). $[\alpha]^{20}_D$: +24.0 (*c* 0.025, MeOH), lit. $[\alpha]^{19}_D$: –36 (*c* 0.81, MeOH) for the enantiomer,[13] $[\alpha]_D$: +50 (*c* 1.0, MeOH).[14] **MS ESI+ *m/z*, (%)**: 394/392 (15/49, $[M+Na]^+$), 372/370 (29/100, $[M+H]^+$). **HRMS ESI+ *m/z*, (%)** ($[M+H]^+$): calcd. for $C_{21}H_{25}ON_3{}^{35}Cl$: 370.1681, found: 370.1682. calcd. for $C_{21}H_{25}ON_3{}^{37}Cl$: 372.1651, found: 372.1652. ($[M+Na]^+$): calcd. for $C_{21}H_{24}ON_3{}^{35}ClNa$: 392.1500, found: 392.1501. calcd. for $C_{21}H_{24}ON_3{}^{37}ClNa$: 394.1470, found: 394.1471. **IR**: ν (cm^{-1}): 3226, 3195, 2923, 2852, 1667, 1634, 1468, 1411, 1061, 800. **^{1}H NMR (500 MHz, CD_3OD)**: δ = 7.32 (d, *J* = 8.3 Hz, 1H, H-4), 7.27 (d, *J* = 1.8 Hz, 1H, H-7), 6.95 (dd, *J* = 8.3, 1.8 Hz, 1H, H-5), 3.46 (d, *J* = 10.3 Hz, 1H, $NC\underline{H}_2C$), 3.07 (ddd, *J* = 9.0, 6.9, 3.7 Hz, 1H, $NC\underline{H}_2CH_2CH_2$), 2.91 (d, *J* = 15.4 Hz, 1H, CH_2 Trp), 2.88 (d, *J* = 15.4 Hz, 1H, CH_2 Trp), 2.54 (ddd, *J* = 12.6, 8.8, 5.9 Hz, 1H, $NCH_2CH_2C\underline{H}_2$), 2.27 (dd, *J* = 10.3, 1.9 Hz, 1H, $NC\underline{H}_2C$), 2.21-2.15 (m, 2H, CH bridge, $NC\underline{H}_2CH_2CH_2$), 2.04-2.01 (m, 1H, CH_2 bridge), 2.00-1.97 (m, 1H, CH_2 bridge), 1.91-1.85 (m, 2H, $NCH_2C\underline{H}_2CH_2$), 1.50-1.46 (m, 1H, $NCH_2CH_2C\underline{H}_2$) 1.44 (s, 3H, CH_3), 1.35 (s, 3H, CH_3) ppm. **^{13}C NMR (126 MHz, CD_3OD)**: δ = 176.7 (CO), 142.4 (C-2), 138.8 (C-7a), 127.8

(C-6), 126.9 (C-3a), 120.0 (C-5), 119.4 (C-4), 111.5 (C-7), 104.9 (C-3), 66.1 (C bridgehead Pro), 59.5 ($N\underline{C}H_2C$), 57.6 (C bridgehead Trp), 55.4 ($N\underline{C}H_2CH_2CH_2$), 48.7 (CH bridge), 35.4 ($\underline{C}(CH_3)_2$), 32.5 (CH_2 bridge), 30.8 (CH_3), 30.2 (CH_2 Trp), 28.1 ($NCH_2CH_2\underline{C}H_2$), 24.3 ($CH_3$), 23.6 ($NCH_2\underline{C}H_2CH_2$) ppm. **^{1}H NMR (600 MHz, DMSO-*d$_6$*)**: δ = 11.08 (s, 1H, NH indole), 8.38 (s, 1H, NH amide), 7.32 (d, *J* = 8.3 Hz, 1H, H-4), 7.28 (d, *J* = 1.9 Hz, 1H, H-7), 6.95 (dd, *J* = 8.3, 1.9 Hz, 1H, H-5), 3.28 (d, *J* = 10.0 Hz, 1H, $NC\underline{H}_2C$), 2.96-2.93 (m, 1H, $NC\underline{H}_2CH_2CH_2$), 2.78 (d, *J* = 16.2 Hz, 1H, CH_2 Trp), 2.75 (d, *J* = 16.2 Hz, 1H, CH_2 Trp), 2.44 (ddd, *J* = 12.3, 9.3, 5.5 Hz, 1H, $NCH_2CH_2C\underline{H}_2$), 2.15 (d, *J* = 10.0 Hz, 1H, $NC\underline{H}_2C$), 2.04-1.96 (m, 2H, CH bridge, $NC\underline{H}_2CH_2CH_2$), 1.88 (dd, *J* = 12.9, 11.2 Hz, CH_2 bridge), 1.81 (dd, *J* = 12.9, 4.6 Hz, 1H, CH_2 bridge), 1.76-1.72 (m, 2H, $NCH_2C\underline{H}_2CH_2$), 1.33 (s, 3H, CH_3), 1.33-1.28 (m, 1H, $NCH_2CH_2C\underline{H}_2$), 1.27 (s, 3H, CH_3) ppm. **^{13}C NMR (151 MHz, DMSO-*d$_6$*)**: 173.1 (CO), 142.6 (C-2), 136.9 (C-7a), 125.4 (C-6), 125.2 (C-3a), 118.7 (C-5), 118.5 (C-4), 110.3 (C-7), 103.7 (C-3), 64.1 (C bridgehead Pro), 58.6 ($N\underline{C}H_2C$), 55.3 (C bridgehead Trp), 53.9 ($N\underline{C}H_2CH_2CH_2$), 47.0 (CH bridge), 34.0 ($\underline{C}(CH_3)_2$), 31.3 (CH_2 bridge), 30.0 (CH_3), 28.7 (CH_2 Trp), 26.6 ($NCH_2CH_2\underline{C}H_2$), 23.7 ($CH_3$), 22.5 ($NCH_2\underline{C}H_2CH_2$) ppm.

Comparison of the NMR data for synthetic malbrancheamide B (1) and *ent*-1 synthesized by Simpkins *et al.*[13] (numbering according to Mata *et al.*[14])

Table S2. ^{1}H NMR data of **1** and *ent*-**1** in d_6-DMSO.

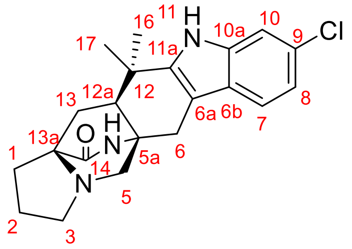

C-H	**1** (δ_H)	*ent*-**1** (δ_H)[13]	Deviation ($\Delta\delta_H$)
1	2.44, ddd, *J* = 12.3, 9.3, 5.5 Hz	2.44, ddd, *J* = 12.5, 9.0, 6.0 Hz	0.00, *J* –0.2, +0.3, –0.5 Hz
	1.33-1.28, m	1.30, m	+ 0.03- –0.02
2	1.76-1.72, m	1.77-1.71, m	–0.01 -+0.01
3	2.96-2.93, m	2.95, m	+0.01 - –0.02
	2.04-1.96, m	2.03, m	+0.01 - –0.07
5	3.28, d, *J* = 10.0 Hz	3.28, d, *J* = 10.0 Hz	0.00
	2.15, d, *J* = 10.0 Hz	2.15, d, *J* = 10.0 Hz	0.00
6	2.78, d, *J* = 16.2 Hz	2.78, d, *J* = 16.0 Hz	0.00, *J* +0.2 Hz
	2.75, d, *J* = 16.2 Hz	2.75, d, *J* = 16.0 Hz	0.00, *J* +0.2 Hz
7	7.32, d, *J* = 8.3 Hz	7.32, d, *J* = 8.5 Hz	0.00, *J* –0.2 Hz
8	6.95, dd, *J* = 8.3, 1.9 Hz	6.95, dd, *J* = 8.5, 2.0 Hz	0.00, *J* –0.2, –0.1 Hz
10	7.28, d, *J* = 1.9 Hz	7.27, d, *J* = 1.5 Hz	+0.01, *J* +0.4 Hz
11	11.03, s	11.03, s	0.00
12a	2.04-1.96, m	2.03, m	+0.01 - –0.07
13	1.88, dd, *J* = 12.9, 11.2 Hz	1.88, dd, *J* = 13.0, 11.0 Hz	0.00, *J* –0.1, +0.2 Hz
	1.81, dd, *J* = 12.9, 4.6 Hz	1.81, dd, *J* = 13.0, 4.5 Hz	0.00, *J* –0.1, +0.1 Hz
15	8.38, s	8.35, s	+0.03
16	1.27, s	1.27, s	0.00
17	1.33, s	1.33, s	0.00

Comparison of the NMR data for synthetic malbrancheamide B (1) and isolated malbracheamide B[14] (numbering according to Mata *et al.*[14])

Table S3. ^{1}H NMR data of **1** in d_6-DMSO.

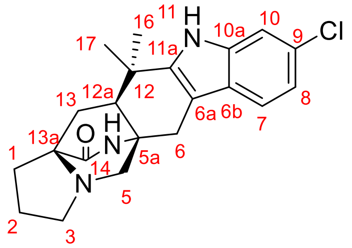

C-H	**1** (δ_H)	Isolation (δ_H)[14]	Deviation ($\Delta\delta_H$)
1	2.44, ddd, *J* = 12.3, 9.3, 5.5 Hz	2.42, m	+0.02
	1.33-1.28, m	1.34, m	–0.01- –0.06
2	1.76-1.72, m	1.72, m	+0.04 - 0.00
3	2.96-2.93, m	2.93, ddd *J* = 9.6, 6.0, 2.1 Hz	+0.03 - 0.00
	2.04-1.96, m	2.41, ddd *J* = 9.6; 6.0, 2.1 Hz	–0.37 - –0.45
5	3.28, d, *J* = 10.0 Hz	3.25, d, *J* = 9.9 Hz	+0.03, *J* +0.1 Hz
	2.15, d, *J* = 10.0 Hz	2.13, d, *J* = 9.9 Hz	+0.02, *J* +0.1 Hz
6	2.78, d, *J* = 16.2 Hz	2.79, d, *J* = 15.9 Hz	–0.01, *J* +0.3 Hz
	2.75, d, *J* = 16.2 Hz	2.72, d, *J* = 15.9 Hz	+0.03, *J* +0.3 Hz
7	7.32, d, *J* = 8.3 Hz	7.31, d, *J* = 8.7 Hz	+0.01, *J* –0.4 Hz
8	6.95, dd, *J* = 8.3, 1.9 Hz	6.94, dd, *J* = 8.4, 1.8 Hz	+0.01, *J* –0.1, +0.1 Hz
10	7.28, d, *J* = 1.9 Hz	7.26, d, *J* = 1.7 Hz	+0.02, *J* +0.2 Hz
11	11.03, s	11.04, s	–0.01
12a	2.04-1.96, m	2.07, m	–0.03 - –0.11
13	1.88, dd, *J* = 12.9, 11.2 Hz	1.98, m	–0.10
	1.81, dd, *J* = 12.9, 4.6 Hz	1.91, m	–0.10
15	8.38, s	8.37, s	+0.01
16	1.27, s	1.26, s	+0.01
17	1.33, s	1.31, s	+0.02

Table S4. ^{13}C NMR spectroscopic data of **1** in d_6-DMSO.

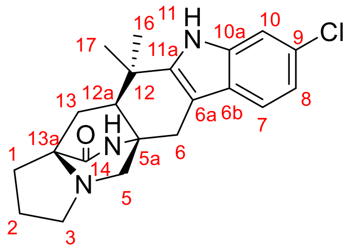

C	**1** (δ_C)	*ent*-**1** (δ_C)[13]	Deviation ($\Delta\delta_C$)[13]	Isolation (δ_C)[14]	Deviation ($\Delta\delta_C$)[14]
1	26.6	26.6	0.0	28.5	–1.9
2	22.5	22.4	+0.1	22.4	+0.1
3	53.9	53.8	+0.1	55.2	–1.3
5	58.6	58.3	+0.3	59.5	–0.9
5a	55.3	55.3	0.0	58.5	–3.2
6	28.7	28.7	0.0	29.9	–1.2
6a	103.7	103.6	+0.1	103.7	0.0
6b	125.2	125.2	0.0	122.7	+2.5
7	118.5	118.4	+0.1	120.7	–2.2
8	118.7	118.6	+0.1	125.6	–6.9
9	125.4	125.3	+0.1	126.6	–1.2
10	110.3	110.3	0.0	112.1	–1.8
10a	136.9	136.8	+0.1	135.4	+1.5
11a	142.6	142.6	0.0	144.4	–1.8
12	34.0	33.9	+0.1	34.1	–0.1
12a	47.0	47.0	0.0	46.9	+0.1
13	31.3	31.1	+0.2	33.9	–2.6
13a	64.1	64.0	+0.1	64.1	0.0
14	173.1	173.0	+0.1	173.1	0.0
16	30.0	30.0	0.0	31.1	–1.1
17	23.7	23.6	+0.1	26.6	–2.9

The ^{1}H and even more the ^{13}C NMR data of isolated[14] and synthetic malbrancheamide (**1**) differ to a significant extent from each other. However, the ^{1}H and ^{13}C NMR data of our, Williams's[15] (not shown) synthetic malbrancheanide B (**1**) and those of synthetic *ent*-**1** by Simpkins *et al.*[13] are in full agreement to each other.

(5a*S*,6a*S*,11a*R*,13a*S*)-9-Chloro-13a-(3-methylbut-2-en-1-yl)-6a-((2,2,6,6-tetramethylpiperidin-1-yl)oxy)-1,2,3,6,6a,11,11a,13a-octahydro-13H-pyrrolo[1'',2'':4',5']pyrazino[1',2':1,5]pyrrolo[2,3-b]indole-5,13(5aH)-dione (20)

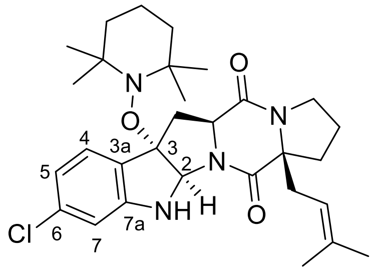

A flame-dried Schlenk tube was charged with diketopiperazine **8a** (40 mg, 0.10 mmol). BSTFA (0.14 mL, 0.52 mmol) and TMSCl (1.39 µL in 0.7 mL of dry CH_3CN) were subsequently added. The solution was stirred at 23 °C for 24 h, the volatiles were evaporated in high vacuum, and the residue was kept in high vacuum at 23 °C overnight. DME (2.3 mL) was added, and the resulting solution was degassed by three freeze-pump-thaw cycles. NaHMDS (1 M in THF, 0.23 mL, 0.23 mmol) was added dropwise at –78 °C. The reaction mixture was stirred at this temperature for 2.5 h and warmed to –40 °C over 30 min. TEMPO (21 mg, 0.13 mmol) was added as a single portion, followed by Cp_2FePF_6 (79 mg, 0.24 mmol) in small portions until the blue color persisted. The reaction mixture was stirred for 10 min, and the septum was exchanged for a condenser. The tube was placed in a pre-heated 100 °C silicone oil bath, the mixture was refluxed for 1.5 h and cooled to 23 °C. The solvent was evaporated under reduced pressure, the residue was dissolved in EtOAc, and the mixture was filtered through a pad of silica gel, which was eluted with EtOAc. The solution was concentrated under reduced pressure and purification of the resulting residue by flash chromatography on silica gel, eluting with 50% EtOAc in cyclohexane, provided **20** as a yellow solid (32 mg, 59%). **R*f***: 0.39 (cyclohexane/EtOAc = 1:1). **M.p.**: 78-80 °C. **$[\alpha]^{20}_D$**: –0.8 (EtOAc, *c* 0.274). **MS ESI+ *m/z*, (%)**: 565/563 (13/80, $[M+Na]^+$), 543/541 (26/100, $[M+H]^+$), 386/384 (1/16, $[(M–TEMPOH)+H]^+$). **HRMS ESI+ *m/z*,** ($[M+Na]^+$): calcd. for $C_{30}H_{41}O_3N_4{}^{35}ClNa$**:** 563.2759, found: 563.2757. Calcd. for $C_{30}H_{41}O_3N_4{}^{37}ClNa$: 565.2730, found: 565.2733. ($[M+H]^+$): calcd. for $C_{30}H_{42}O_3N_4{}^{35}Cl$: 541.2938, found: 541.2940. Calcd. for $C_{30}H_{42}O_3N_4{}^{37}Cl$: 543.2911, found: 543.2915. **IR**: ν (cm^{-1}) 3655, 2924, 2854, 1637, 1458, 1376, 1310, 1182, 1068, 841, 742. **^{1}H NMR (500 MHz, CD_3CN)**: δ = 7.29 (d, *J* = 8.0 Hz, 1H, H-4), 6.68 (dd, *J* = 8.0, 1.9 Hz, 1H, H-5), 6.60 (d, *J* = 1.9 Hz, 1H, H-7), 6.12 (br s, 1H, NH), 5.77 (d, *J* = 1.3 Hz, 1H, H-2), 4.66-4.62 (m, 1H, $CH_2C\underline{H}=C(CH_3)_2$), 4.45 (dd, *J* = 12.4, 6.2 Hz, 1H, $C\underline{H}CH_2$), 3.81-3.75 (m, 1H, $NC\underline{H_2}CH_2CH_2$), 3.31-3.25 (m, 1H, $NC\underline{H_2}CH_2CH_2$), 3.03

(dd, J = 13.1, 6.2 Hz, 1H, $CHC\underline{H}_2$), 2.37-2.33 (m, 1H, $C\underline{H}_2CH{=}C(CH_3)_2$), 2.31-2.27 (m, 1H, $C\underline{H}_2CH{=}C(CH_3)_2$), 2.11-2.06 (m, 2H, $NCH_2CH_2C\underline{H}_2$, $NCH_2C\underline{H}_2CH_2$), 2.03-1.99 (m, 3H, $CHC\underline{H}_2$, $NCH_2CH_2C\underline{H}_2$, $NCH_2C\underline{H}_2CH_2$), 1.57-1.53 (m, 2H, CH_2 TMP), 1.47-1.42 (m, 3H, CH_2 TMP), 1.39 (d, J = 1.4 Hz, 3H, $C(\underline{C}H_3)_2$), 1.34-1.32 (m, 1H, CH_2 TMP), 1.11 (s, 3H, CH_3 TMP), 1.06 (s, 3H, CH_3 TMP), 0.98 (d, 1.3 Hz, 3H, $C(\underline{C}H_3)_2$), 0.94 (s, 3H, CH_3 TMP), 0.80 (s, 3H, CH_3 TMP) ppm. **^{13}C NMR (126 MHz, CD_3CN)**: δ = 167.9 ($\underline{C}ONCH_2CH_2$), 164.1 ($\underline{C}ONCH$), 152.0 (C-7a), 137.7 ($CH_2CH{=}\underline{C}(CH_3)_2$), 135.4 (C-6), 130.4 (C-3a), 128.1 (C-4), 118.6 ($CH_2\underline{C}H{=}C(CH_3)_2$), 118.4 (C-5. Overlaps with the CD_3CN signal), 109.6 (C-7), 96.1 (C-3), 82.0 (C-2), 70.8 (C Pro), 61.2 (C TMP), 60.3 (C TMP), 59.5 ($\underline{C}HCH_2$), 45.8 ($CH\underline{C}H_2$), 44.8 ($N\underline{C}H_2CH_2CH_2$), 41.5 ($CH_2$ TMP), 40.9 (CH_2 TMP), 37.3 ($\underline{C}H_2CH{=}C(CH_3)_2$), 34.8 ($NCH_2CH_2\underline{C}H_2$), 34.1 ($CH_3$ TMP), 33.7 (CH_3 TMP), 25.4 ($C(\underline{C}H_3)_2$), 21.0 (CH_3 TMP), 20.8 (CH_3 TMP), 20.3 ($NCH_2\underline{C}H_2CH_2$), 17.7 ($C(\underline{C}H_3)_2$), 17.6 ($CH_2$ TMP) ppm.

(5a*S*,14a*S*,16*S*)-9-Chloro-16-(prop-1-en-2-yl)-2,3,11,12-tetrahydro-1H,5H,6H,14H-5a,14a-ethanopyrrolo[1'',2'':4',5']pyrazino[1',2':1,6]pyrido[3,4-b]indole-5,14-dione (23)

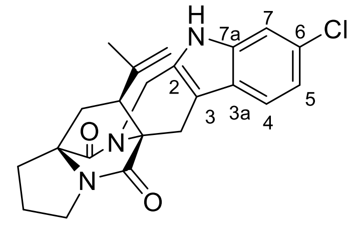

A Schlenk tube was charged with a solution of diketopiperazine **15** (10 mg, 0.021 mmol) in DME (1 mL). Conc. aq. HCl (0.5 mL) was added, the rubber septum was exchanged for a glass stopper and the N_2 inlet tube was closed. The tube was placed in a 65 °C silicone oil bath, the reaction mixture was stirred for 20 min and cooled to 0 °C. EtOAc (4 mL) was added, followed by 10 M NaOH solution (0.6 mL). The mixture was stirred at 0 °C for 10 min, the layers were separated and the aqueous was extracted with EtOAc (4x4 mL). The combined organic layers were dried over $MgSO_4$, filtered and concentrated under reduced pressure, providing diketopiperazines **17** and **23** as a 1:1 mixture (4 mg, **17**: 24%, **23**: 24%). **Data for compound 23**: **MS ESI+ *m/z*, (%)**: 817/815/813 (2/4/7, $[2M+Na]^+$), 420/418 (29/100, $[M+Na]^+$). **HRMS ESI+ *m/z*, (%)**: ($[M+Na]^+$): calcd. for

$C_{22}H_{22}O_2N_3{}^{35}ClNa$: 418.1293, found: 418.1291. calcd. for $C_{22}H_{22}O_2N_3{}^{37}ClNa$: 420.1263, found: 420.1261. **^{1}H NMR (500 MHz, CD_3CN)**: δ = 9.31 (br s, NH), 7.47 (d, *J* = 8.4 Hz, 1H, H-4), 7.41 (d, *J* = 1.9 Hz, 1H, overlaps with the signal of compound **17**, H-7), 7.05 (dd, *J* = 8.4, 1.9 Hz, H-5), 4.90-4.89 (m, 1H, $C{=}CH_2$), 4.67 (d, *J* = 16.1 Hz, 1H, $NC\underline{H}_2CNH$), 4.63-4.62 (m, 1H, $C{=}CH_2$), 4.50 (dd, *J* = 16.1, 1.0, $NC\underline{H}_2CNH$), 3.49-3.42 (m, 3H, CH_2 Trp, $NC\underline{H}_2CH_2CH_2$), 2.97 (d, *J* = 15.8 Hz, 1H, CH_2 Trp), 2.93-2.90 (m, 1H, CH bridge), 2.69-2.66 (m, 1H, overlaps with the signal of compound **17**, $NCH_2CH_2C\underline{H}_2$), 2.14-2.10 (m, 1H, overlaps with the signal of compound **17**, CH_2 bridge), 2.03-2.01 (m, 2H, overlaps with the signals of compound **17**, $NCH_2CH_2C\underline{H}_2$, $NCH_2C\underline{H}_2CH_2$), 1.92-1.86 (m, 2H, overlaps with the signals of compound **17**, CH_2 bridge, $NCH_2C\underline{H}_2CH_2$), 1.65 (dd, *J* = 1.4, 0.8 Hz, 1H, CH_3) ppm. **^{13}C NMR (126 MHz, CD_3CN)**: δ = 172.4 ($\underline{C}ONCH_2C$), 168.6 ($\underline{C}ONCH_2CH_2$), 144.9 ($\underline{C}{=}CH_2$), 137.6 (C-7a), 132.2 (C-2), 127.4 (C-6), 126.2 (C-3a), 120.5 (C-5), 119.7 (C-4), 116.4 ($C{=}\underline{C}H_2$), 112.1 (C-7), 107.0 (C-3), 67.2 (C bridgehead Pro), 65.8 (C bridgehead Trp), 52.0 (CH bridge), 44.8 ($N\underline{C}H_2CH_2CH_2$), 39.1 ($N\underline{C}H_2CNH$), 36.6 ($CH_2$ bridge), 30.2 ($NCH_2CH_2\underline{C}H_2$), 24.9 ($NCH_2\underline{C}H_2CH_2$), 22.4 ($CH_2$ Trp), 19.1 (CH_3) ppm.

5. References

(1) Lesma, G.; Cecchi, R.; Cagnotto, A.; Gobbi, M.; Meneghetti, F.; Musolino, M.; Sacchetti, A.; Silvani, A. Tetrahydro-β-carboline-Based Spirocyclic Lactam as Type II′ β-Turn: Application to the Synthesis and Biological Evaluation of Somatostatine Mimetics. *J. Org. Chem.* **2013**, *78*, 2600–2610.

(2) Feng, Y.; Holte, D.; Zoller, J.; Umemiya, S.; Simke, L. R.; Baran, P. S. Total Synthesis of Verruculogen and Fumitremorgin A Enabled by Ligand-Controlled C–H Borylation. *J. Am. Chem. Soc.* **2015**, *137*, 10160–10163.

(3) Murphy, J. M.; Liao, X.; Hartwig, J. F. Meta Halogenation of 1,3-Disubstituted Arenes via Iridium-Catalyzed Arene Borylation. *J. Am. Chem. Soc.* **2007**, *129*, 15434–15435.

(4) Karmel, C.; Chen, Z.; Hartwig, J. F. Iridium-Catalyzed Silylation of C–H Bonds in Unactivated Arenes: A Sterically Encumbered Phenanthroline Ligand Accelerates Catalysis. *J. Am. Chem. Soc.* **2019**, *141*, 7063–7072.

(5) Dokli, I.; Pohl, R.; Klepetářová, B.; Jahn, U. First total synthesis of *ent*-asperparaline C and assignment of the absolute configuration of asperparaline C. *Chem. Commun.* **2019**, *55*, 3931–3934.

(6) Šimek, M.; Bártová, K.; Issad, S.; Hájek, M.; Císařová, I.; Jahn, U. Unified Total Synthesis of Diverse Meroterpenoids from *Ganoderma Applanatum*. *Org. Lett.* **2022**, *24*, 4552–4556.

(7) Barnes, D. M.; Barkalow, J.; Plata, D. J. A Facile Method for the Preparation of MOM-Protected Carbamates. *Org. Lett.* **2009**, *11*, 273–275.

(8) Han, J. H.; Kwon, Y. E.; Sohn, J.-H.; Ryu, D. H. A facile method for the rapid and selective deprotection of methoxymethyl (MOM) ethers. *Tetrahedron* **2010**, *66*, 1673–1677.

(9) Denoël, T.; Zervosen, A.; Gerards, T.; Lemaire, C.; Joris, B.; Blanot, D.; Luxen, A. Stereoselective synthesis of lanthionine derivatives in aqueous solution and their incorporation into the peptidoglycan of *Escherichia coli*. *Bioorg. Med. Chem.* **2014**, *22*, 4621–4628.

(10) Bartoccini, F.; Fanini, F.; Retini, M.; Piersanti, G. General synthesis of unnatural 4-, 5-, 6-, and 7-bromo-D-tryptophans by means of a regioselective indole alkylation. *Tetrahedron Lett.* **2020**, *61*, 151923.

(11) Seebach, D.; Sting, A. R.; Hoffmann, M. Self-regeneration of stereocenters (SRS)-applications, limitations, and abandonment of a synthetic principle. *Angew. Chem. Int. Ed. Engl.* **1996**, *35*, 2708–2748.

(12) Artman III, G. D.; Rafferty, R. J.; Williams, R. M.; Aaron, G. L.; Davis, M. M.; Brummond, K. M. (3*R*, 7a*S*)-3-(Trichloromethyl)Tetrahydropyrrolo[1,2-C]Oxazol-1(3*H*)-One: An Air and Moisture Stable Reagent for the Synthesis of Optically Active α-Branched Prolines. *Org. Synth.* **2009**, *86*, 262–273.

(13) Frebault, F.; Simpkins, N. S.; Fenwick, A. Concise Enantioselective Synthesis of *ent*-Malbrancheamide B. *J. Am. Chem. Soc.* **2009**, *131*, 4214–4215.

(14) Figueroa, M.; González, M. D. C.; Mata, R. Malbrancheamide B, a novel compound from the fungus Malbranchea aurantiaca. *Nat. Prod. Res*. **2008**, *22*, 709−714.

(15) Miller, K. A.; Welch, T. R.; Greshock, T. J.; Ding, Y.; Sherman, D. H.; Williams, R. M. Biomimetic Total Synthesis of Malbrancheamide and Malbrancheamide B. *J. Org. Chem.* **2008**, *73*, 3116–3119.

1. Copies of ^{1}H and ^{13}C NMR spectra

Methyl (*S*)-3-(*tert*-butoxycarbonyl)-1,2,3-oxathiazolidine-4-carboxylate-2,2-dioxide (12) in $CDCl_3$ (400 MHz)

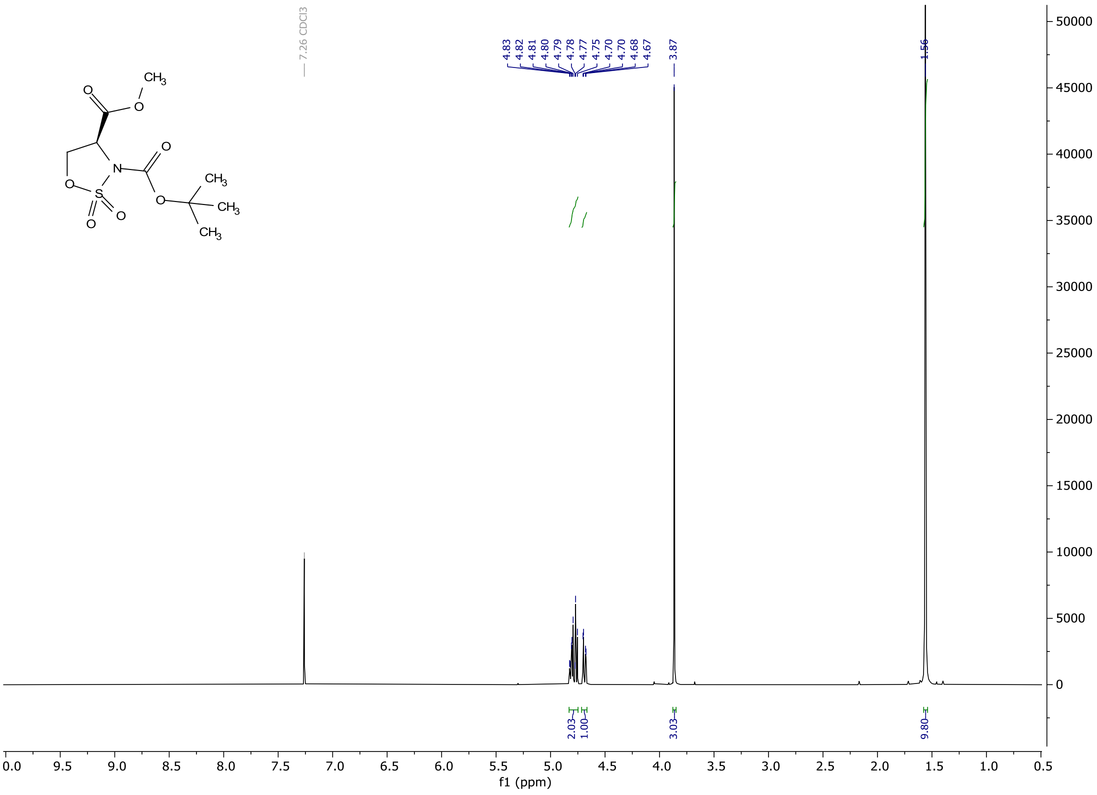

in $CDCl_3$ (100.6 MHz)

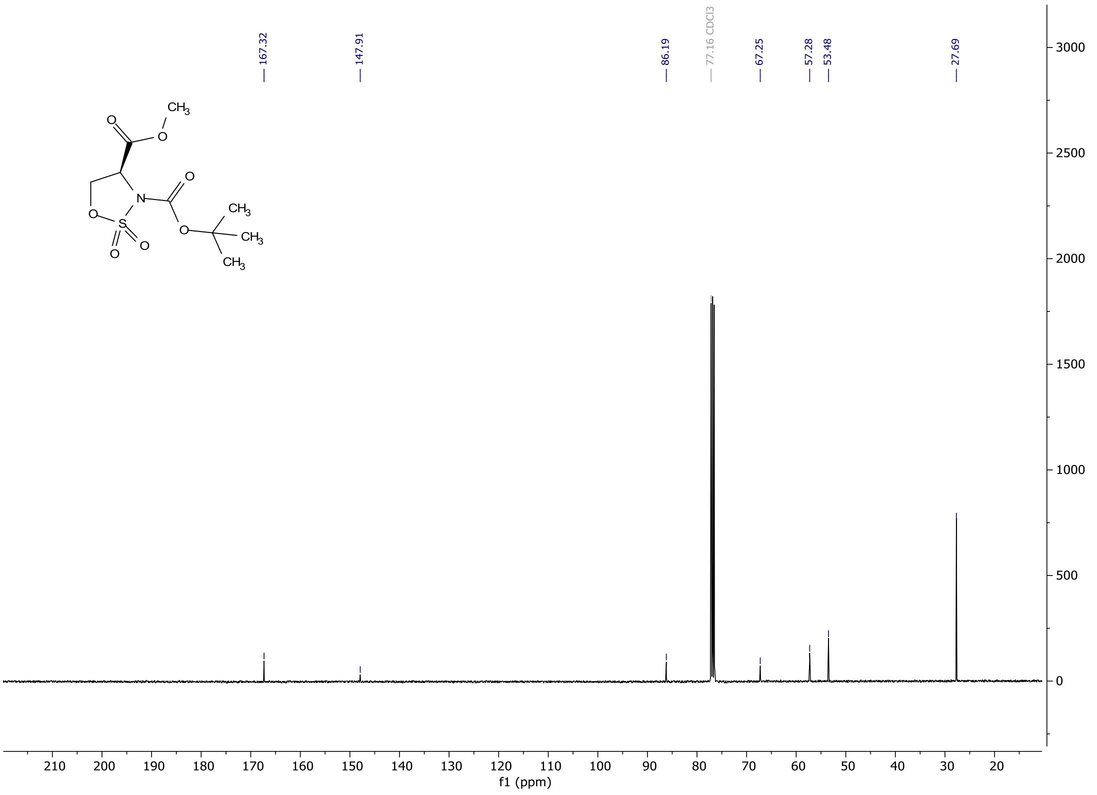

Methyl (*S*)-3-(*tert*-butoxycarbonyl)-1,2,3-oxathiazolidine-4-carboxylate-2,2-dioxide (12) in $CDCl_3$

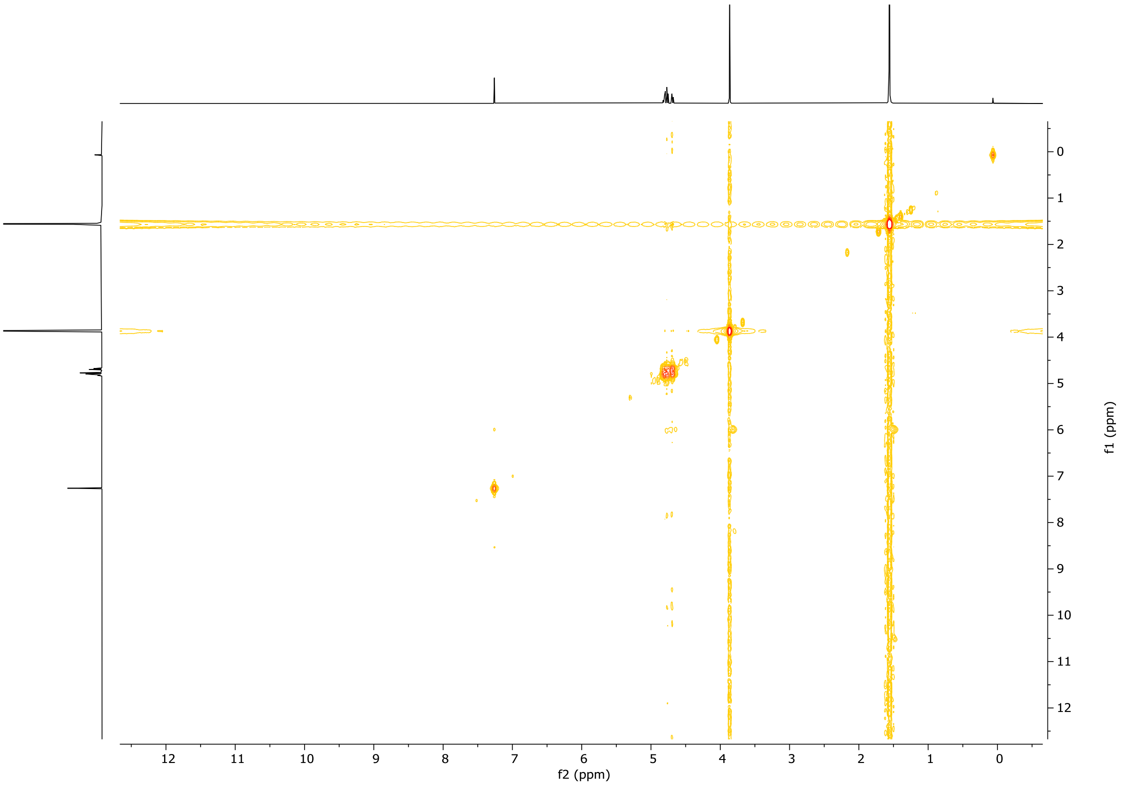

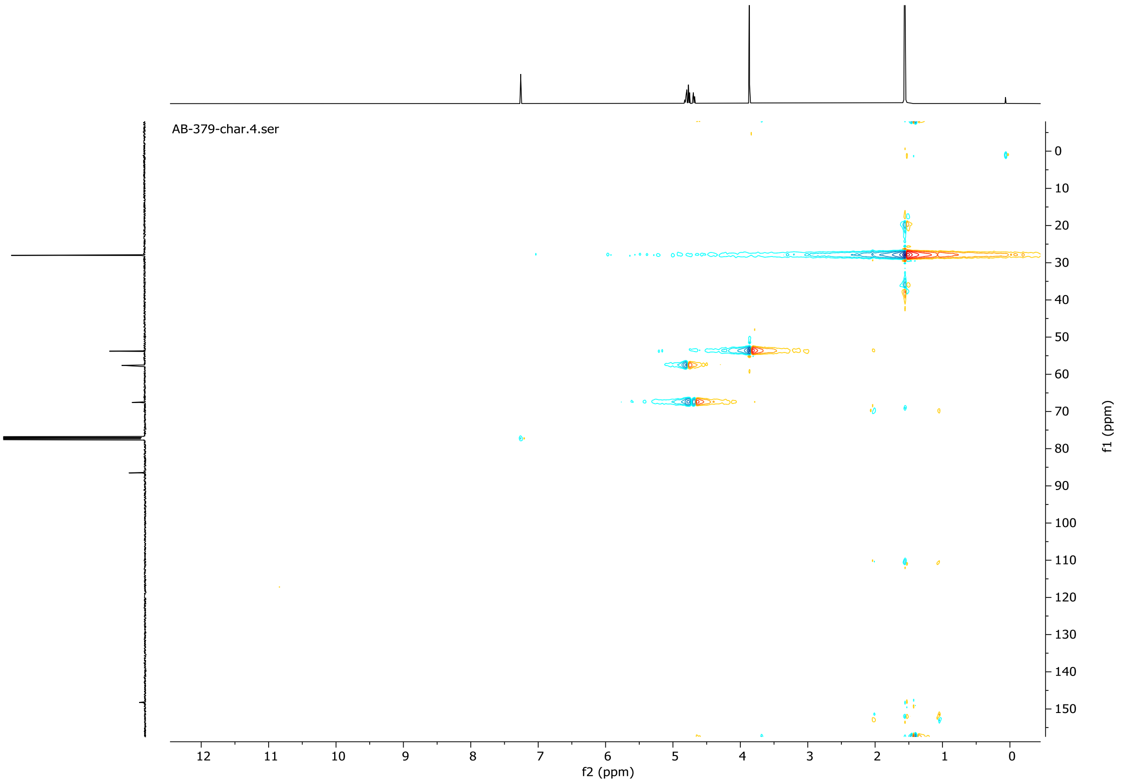

(*S*)-N^{α}-(*tert*-Butoxycarbonyl)-6-chlorotryptophan methyl ester (13) in $CDCl_3$ (400 MHz)

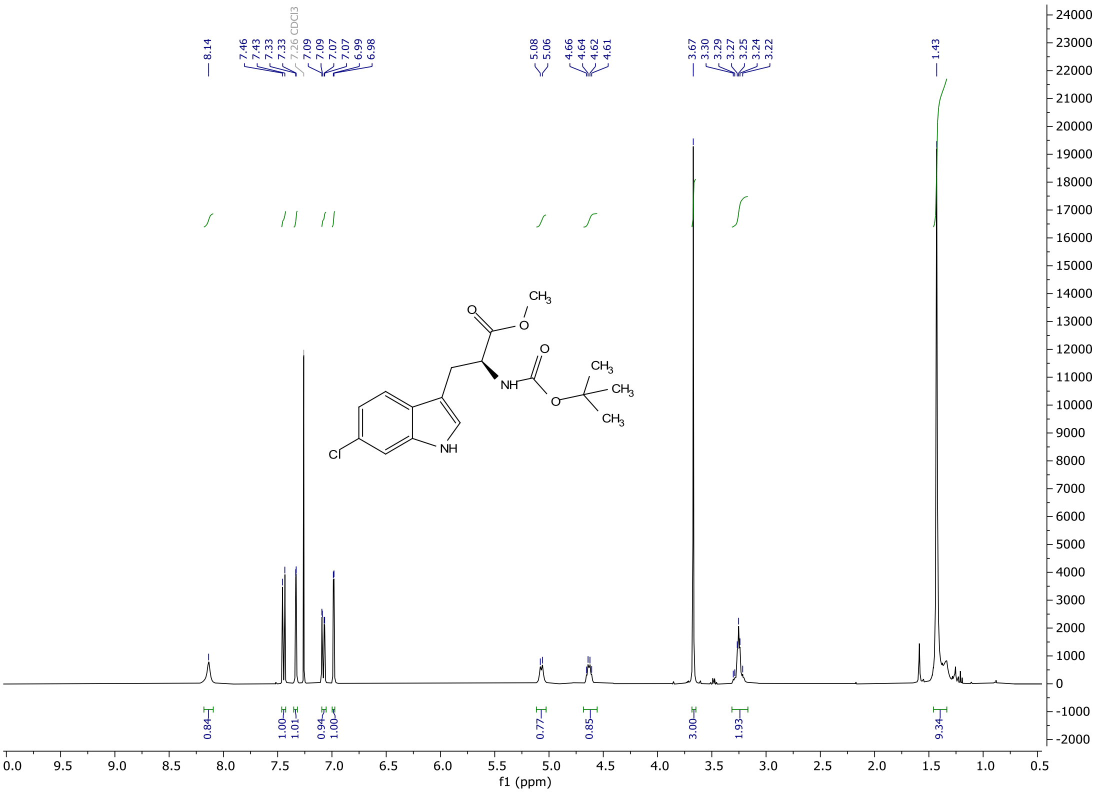

in $CDCl_3$ (100.6 MHz)

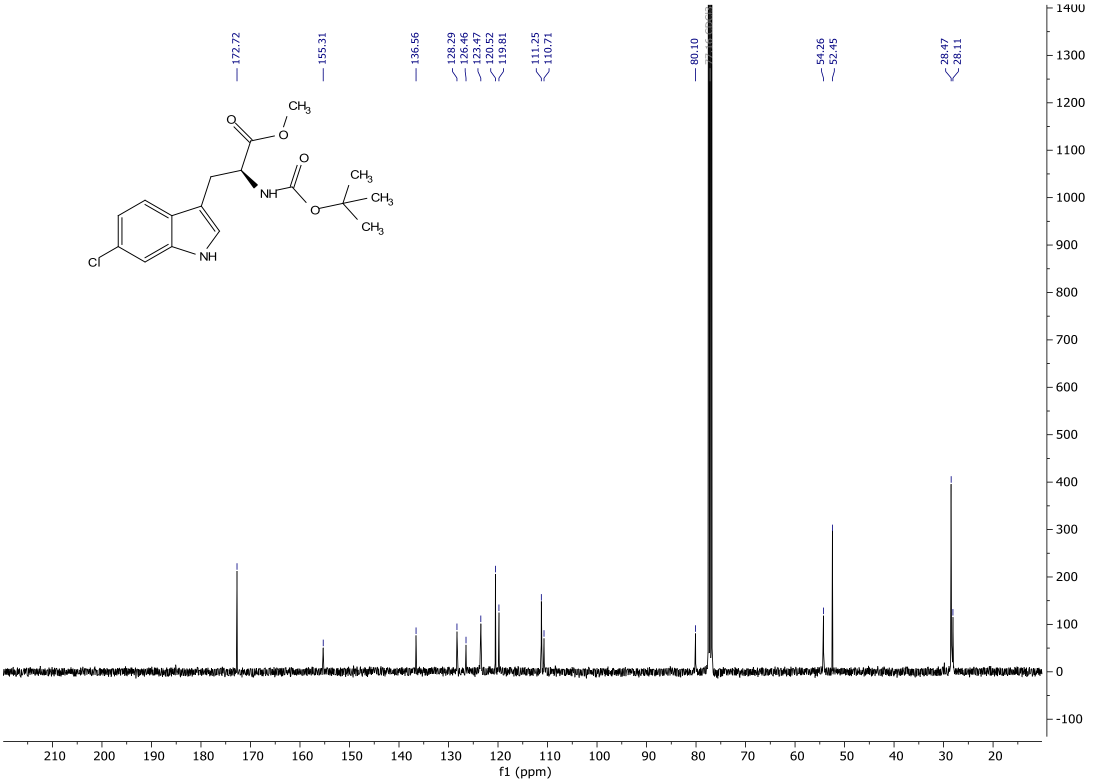

(*S*)-*N*[α]-(*tert*-Butoxycarbonyl)-6-chlorotryptophan methyl ester (13) in $CDCl_3$

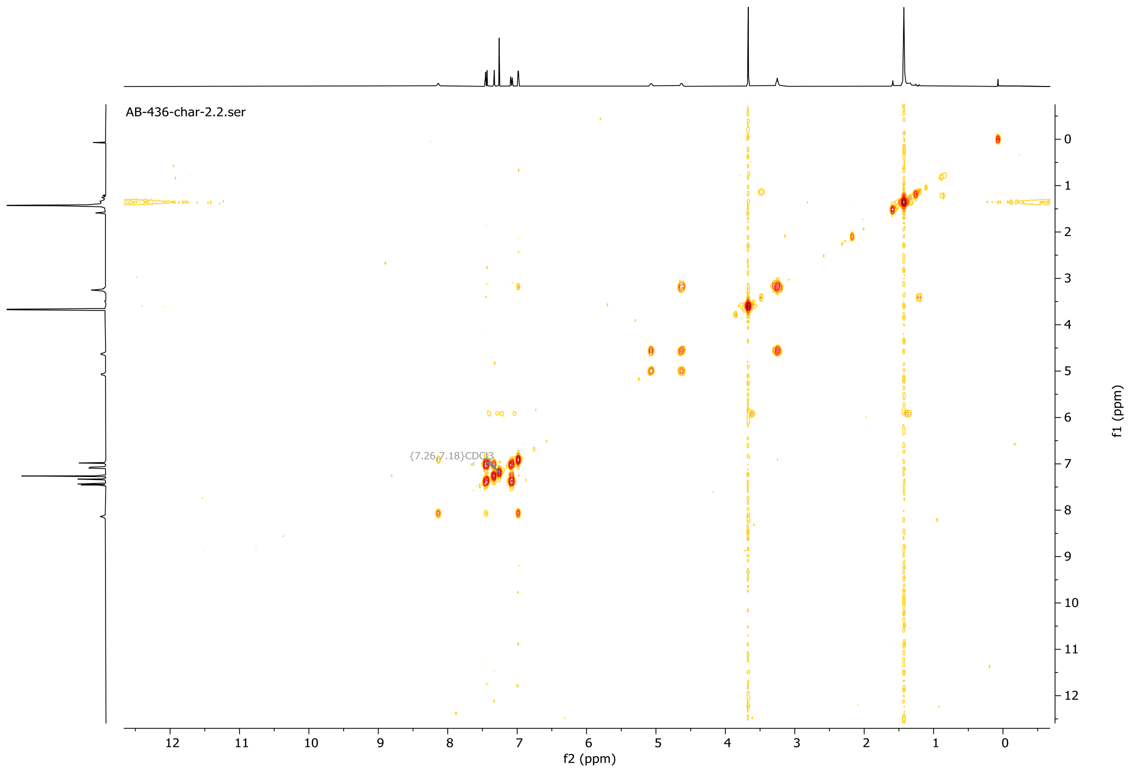

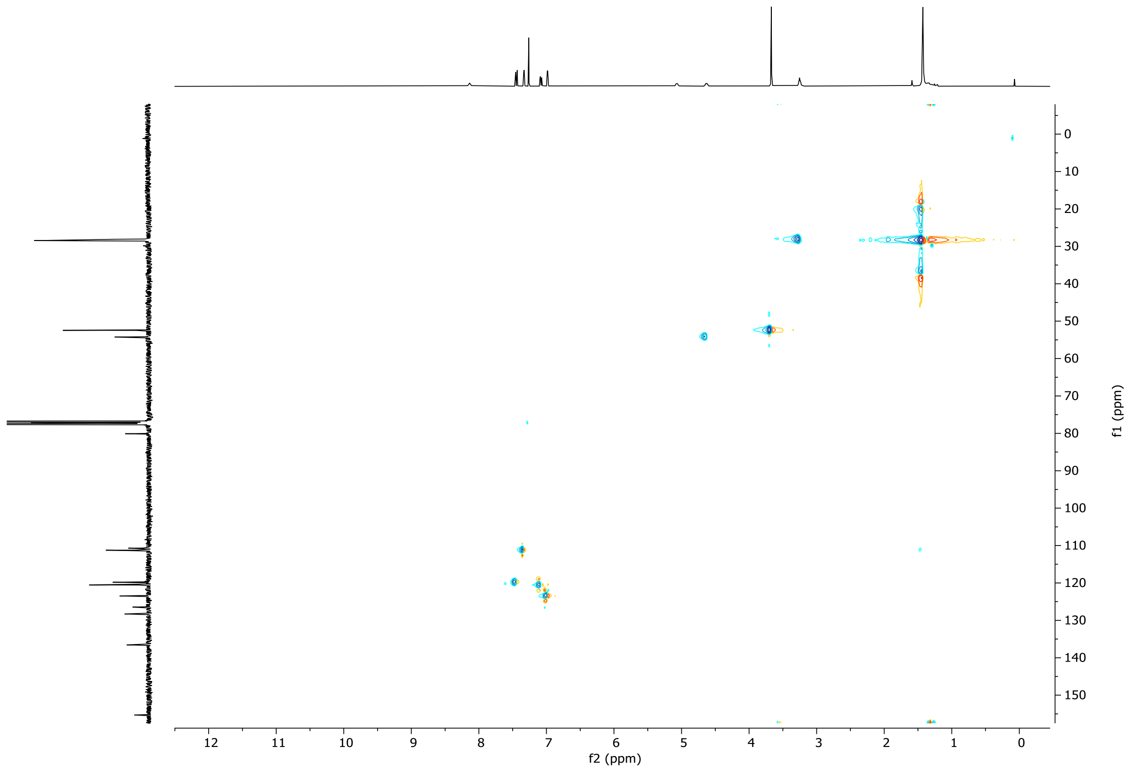

(*S*)-*N*^{α}-(*tert*-Butoxycarbonyl)-6-chlorotryptophan (5) in CD_3CN (400 MHz)

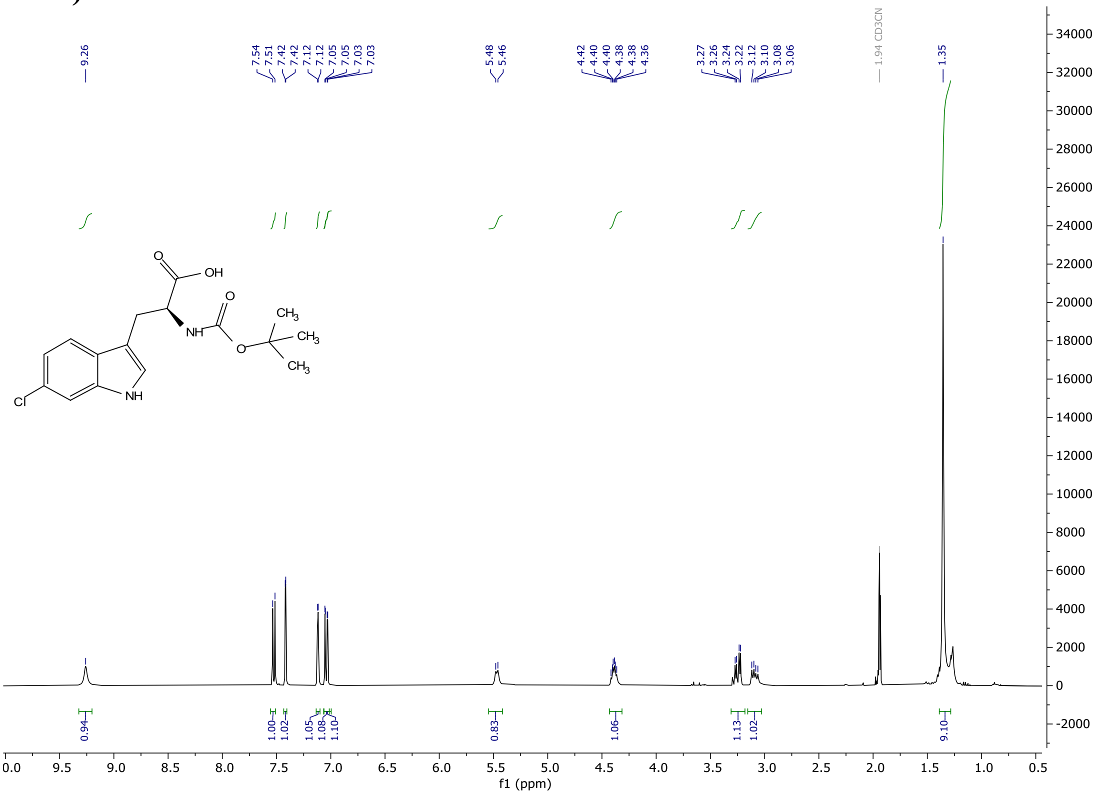

in CD_3CN (100.6 MHz)

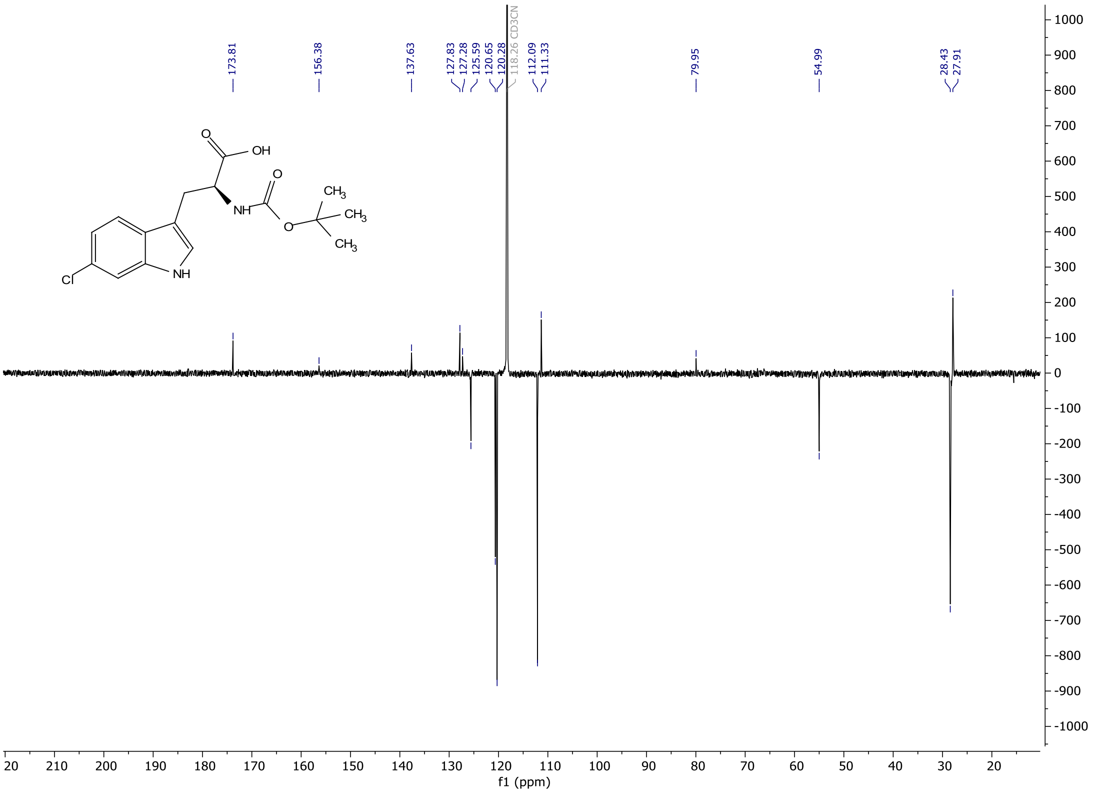

(*S*)-*N*[α]-(*tert*-Butoxycarbonyl)-6-chlorotryptophan (5) in CD_3CN

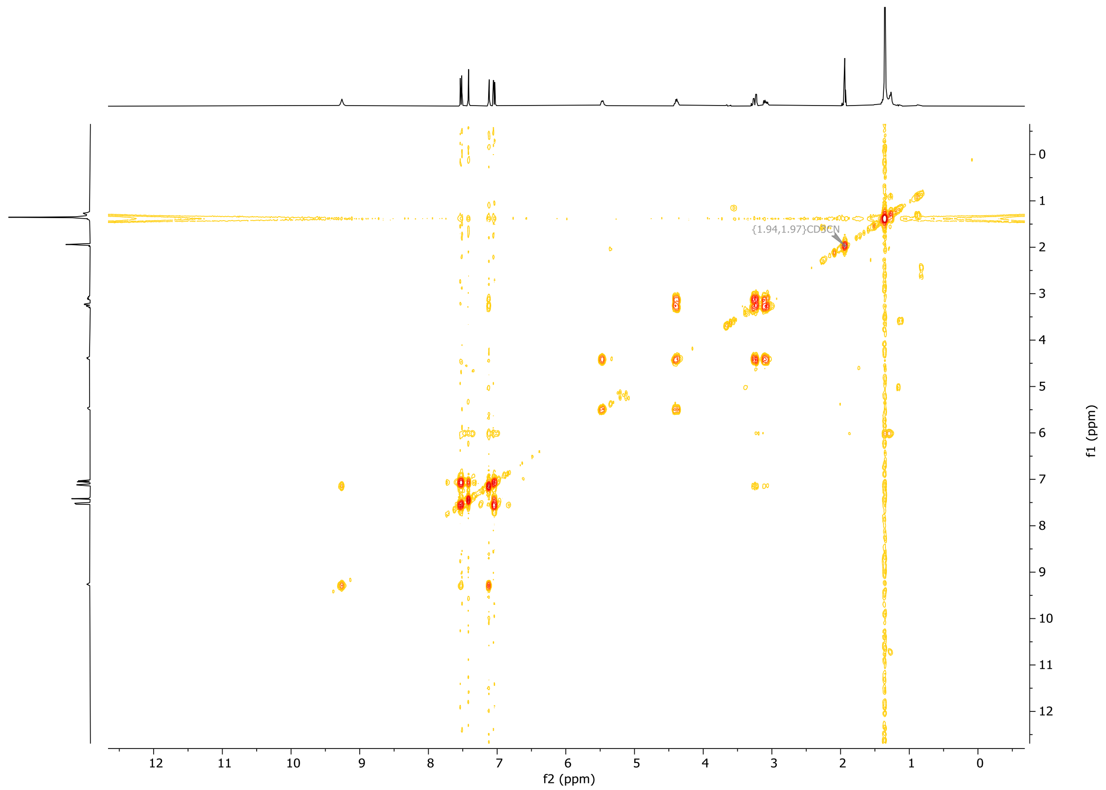

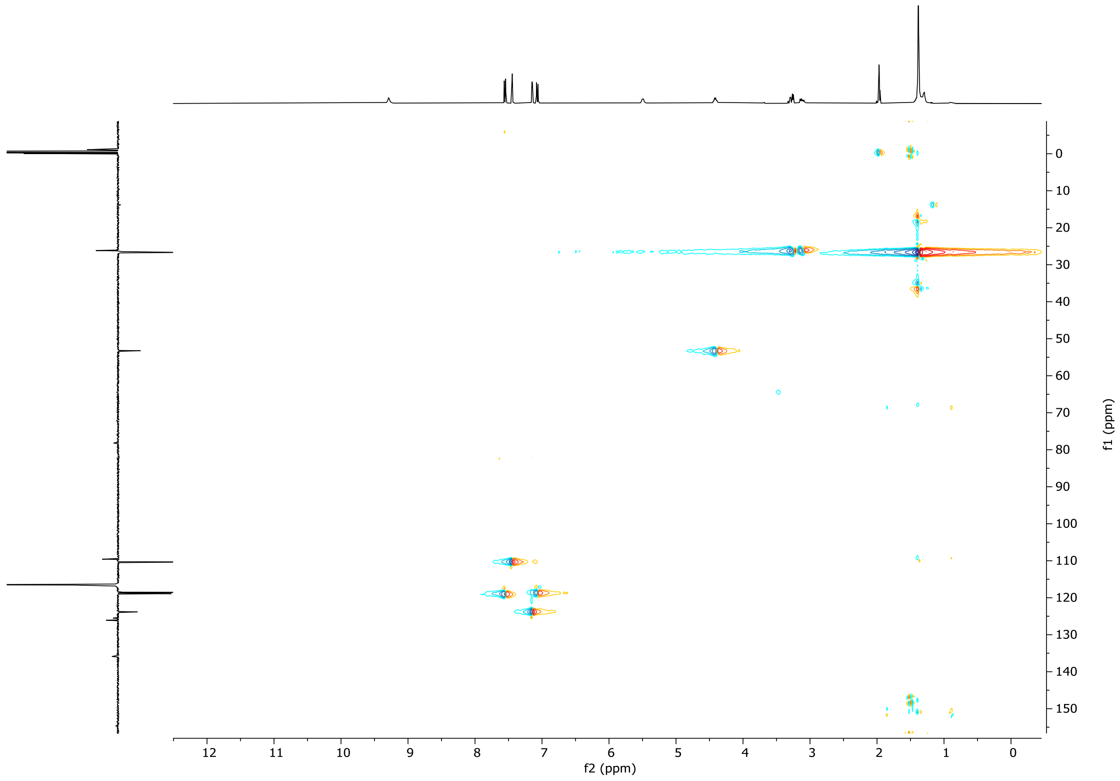

(*S*)-2-(3-Methylbut-2-en-1-yl)proline methyl ester (11) in $CDCl_3$ (400 MHz)

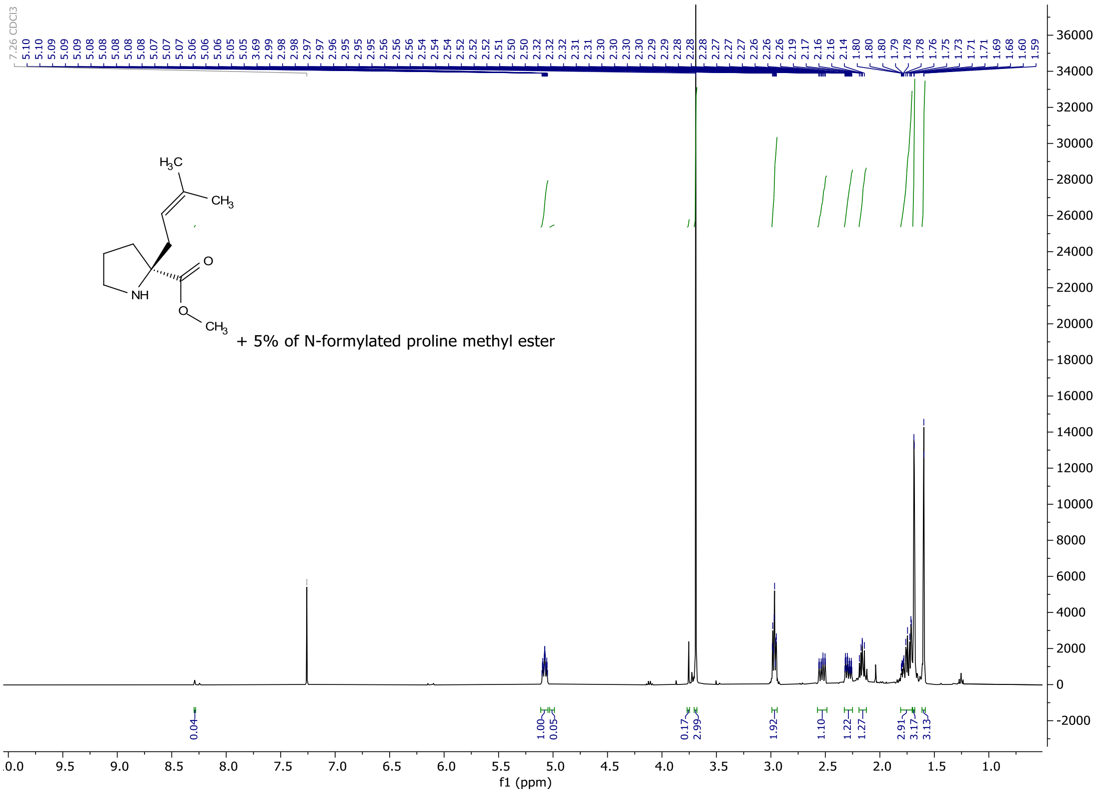

in $CDCl_3$ (100.6 MHz)

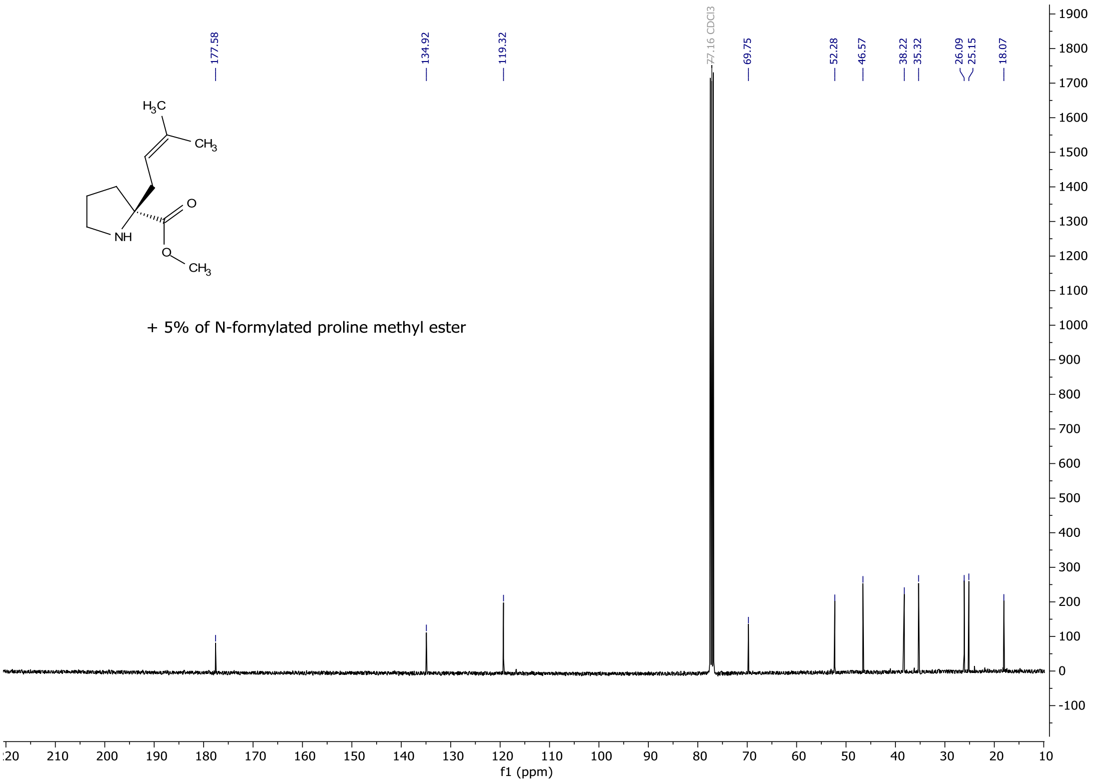

(*S*)-2-(3-Methylbut-2-en-1-yl)proline methyl ester (11) in $CDCl_3$

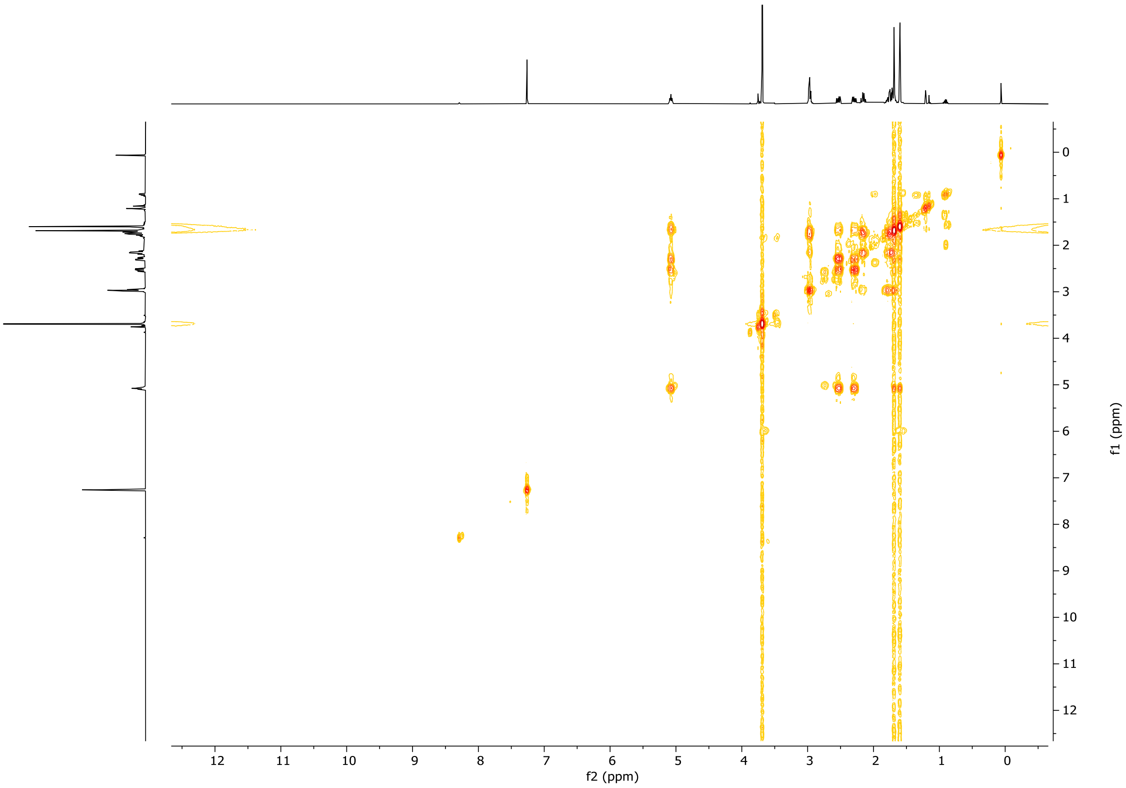

Methyl (*S*)-1-((*S*)-2-amino-3-(6-chloro-1H-indol-3-yl)propanoyl)-2-(3-methylbut-2-en-1-yl)pyrrolidine-2-carboxylate (14) in CDCl$_3$ (400 MHz)

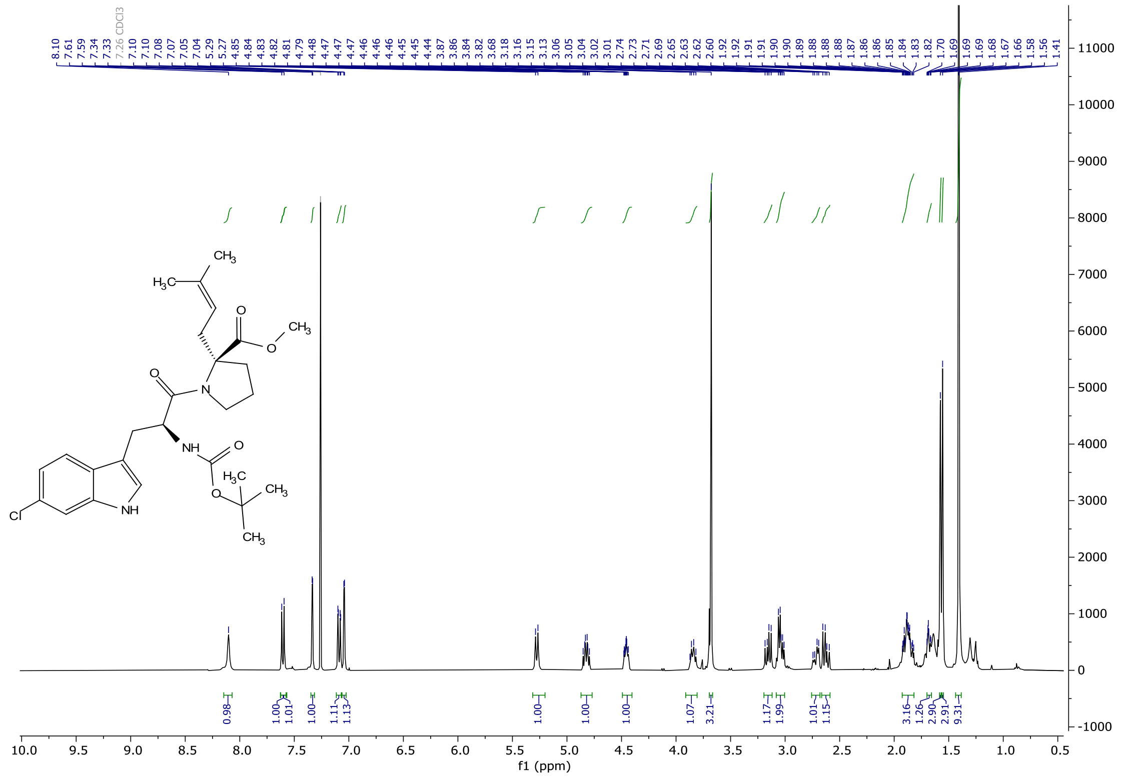

in CDCl$_3$ (100.6 MHz)

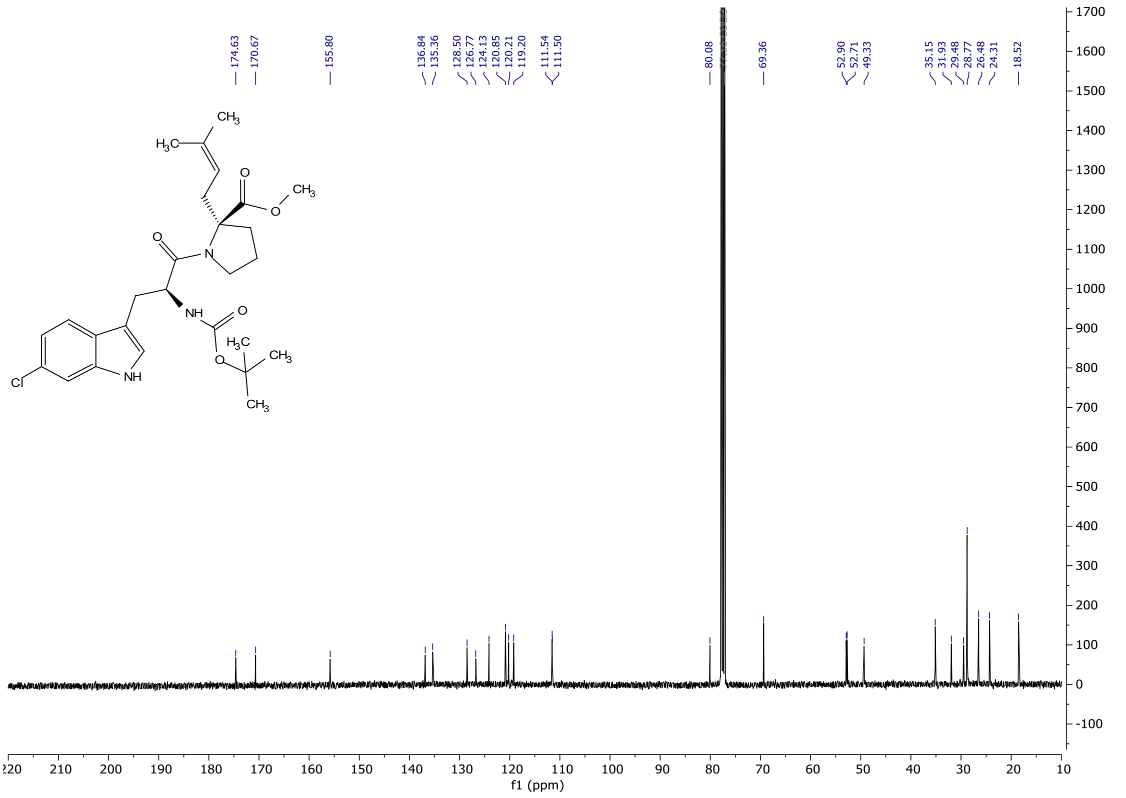

Methyl (*S*)-1-((*S*)-2-amino-3-(6-chloro-1H-indol-3-yl)propanoyl)-2-(3-methylbut-2-en-1-yl)pyrrolidine-2-carboxylate (14) in $CDCl_3$

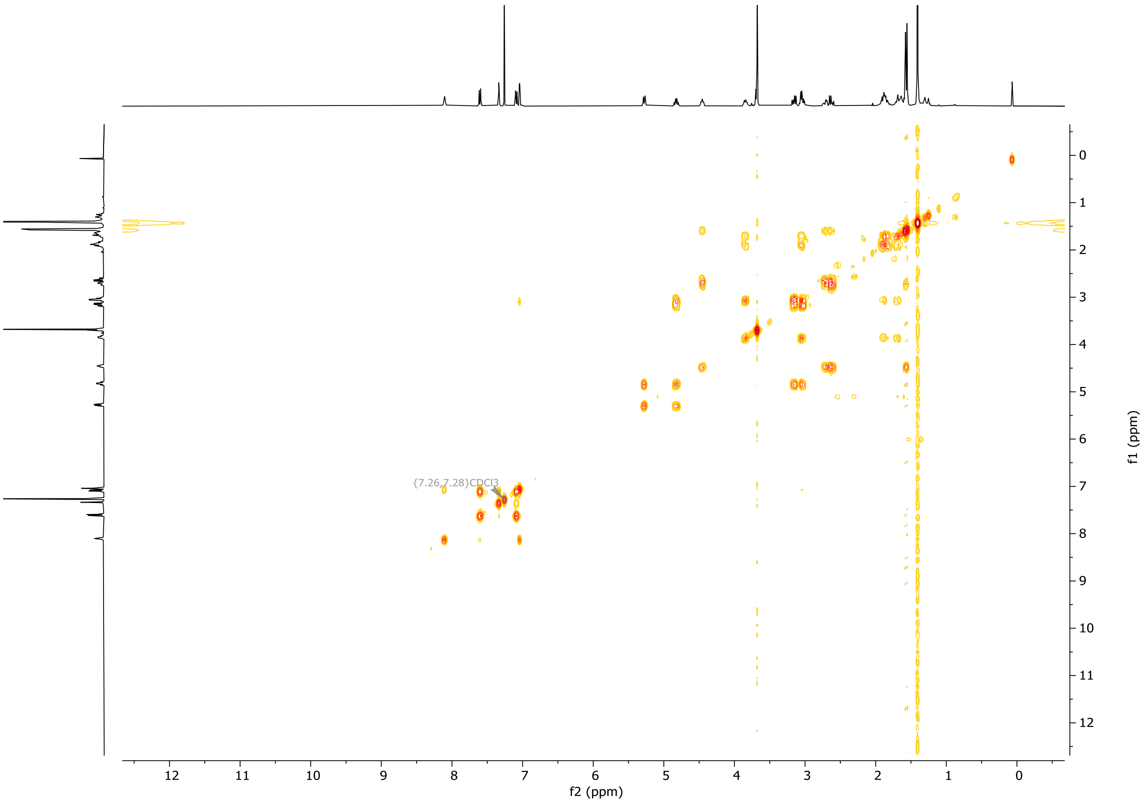

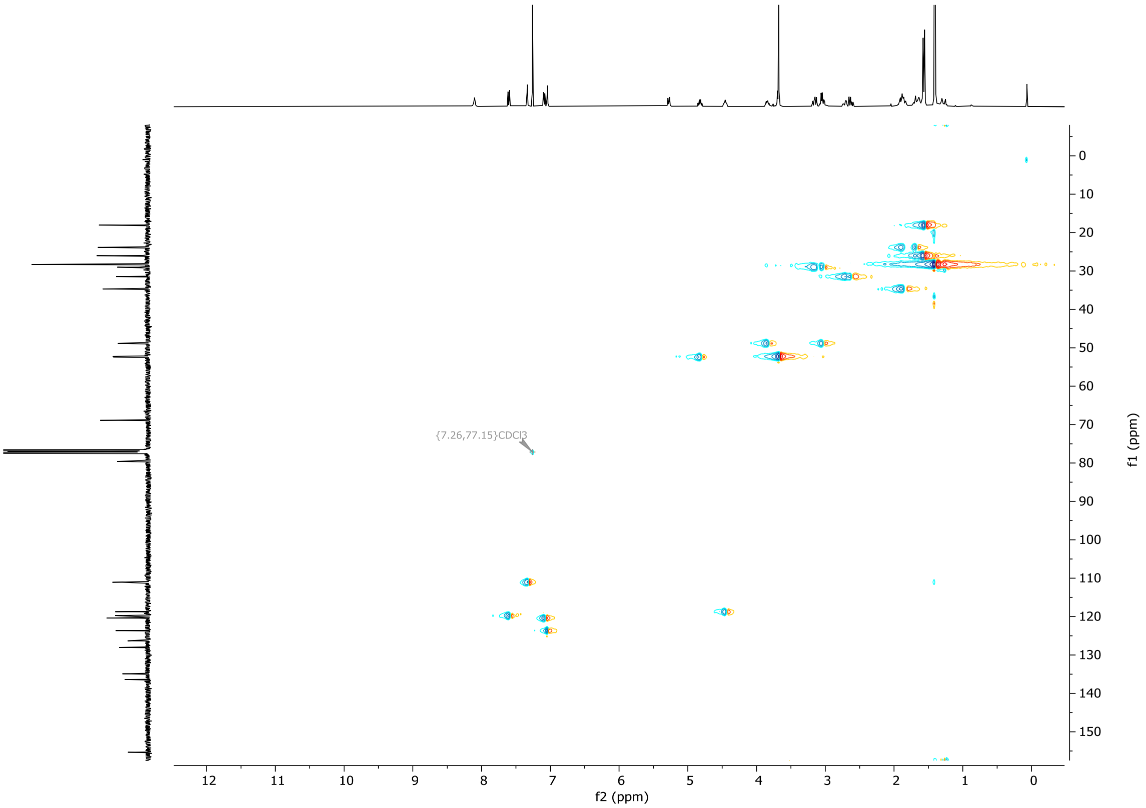

(3*S*,8a*S*)-3-((6-Chloro-1H-indol-3-yl)methyl)-8a-(3-methylbut-2-en-1-yl)hexahydropyrrolo[1,2-a]pyrazine-1,4-dione (8a) in $CDCl_3$ (400 MHz)

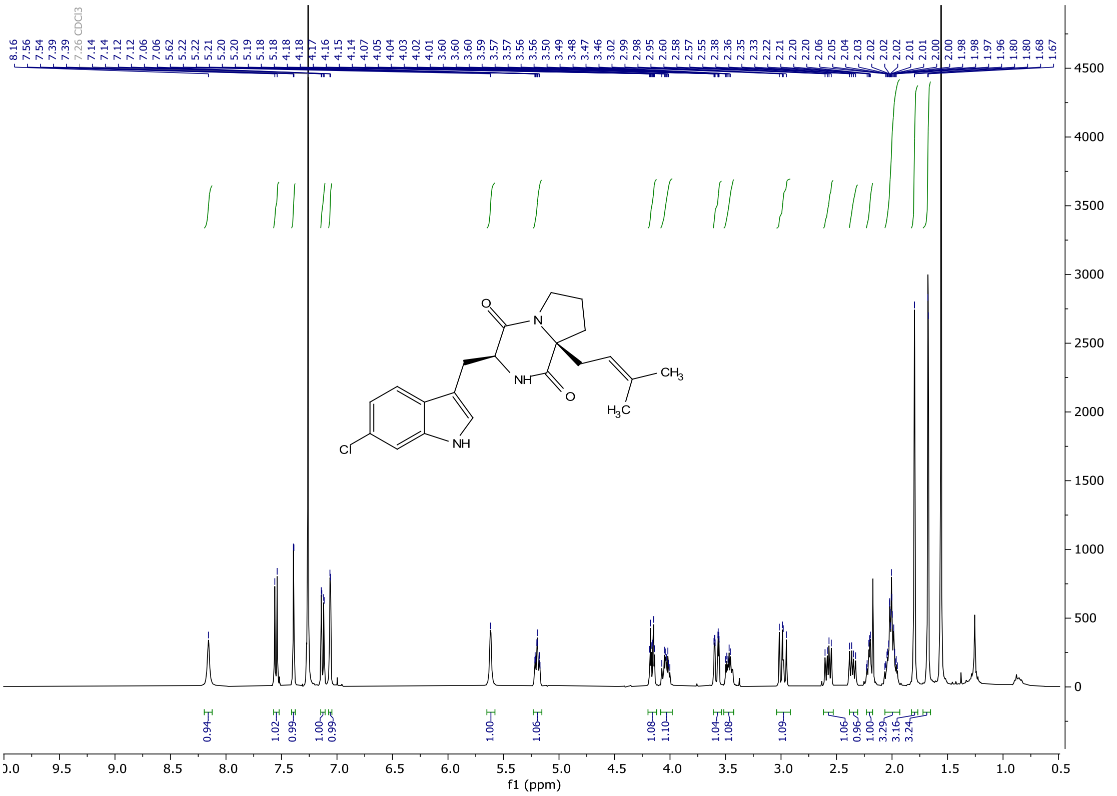

in $CDCl_3$ (100.6 MHz)

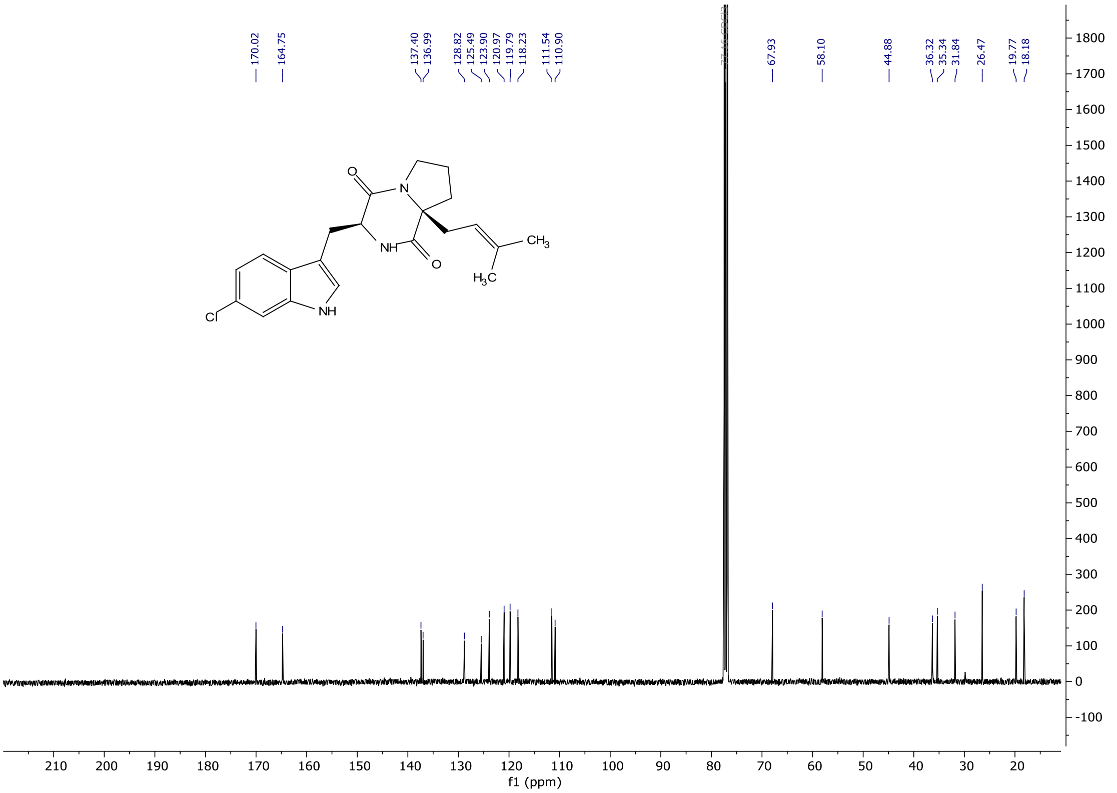

(3*S*,8a*S*)-3-((6-Chloro-1H-indol-3-yl)methyl)-8a-(3-methylbut-2-en-1-yl)hexahydropyrrolo[1,2-a]pyrazine-1,4-dione (8a) in $CDCl_3$

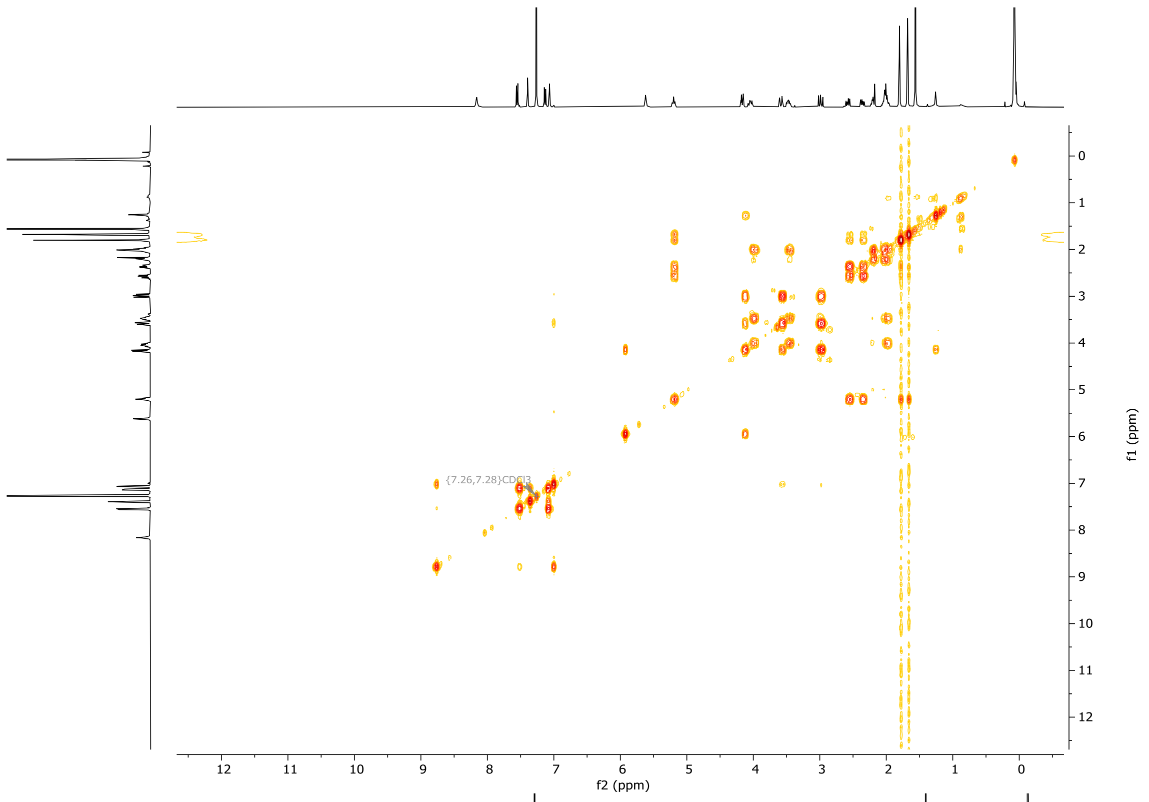

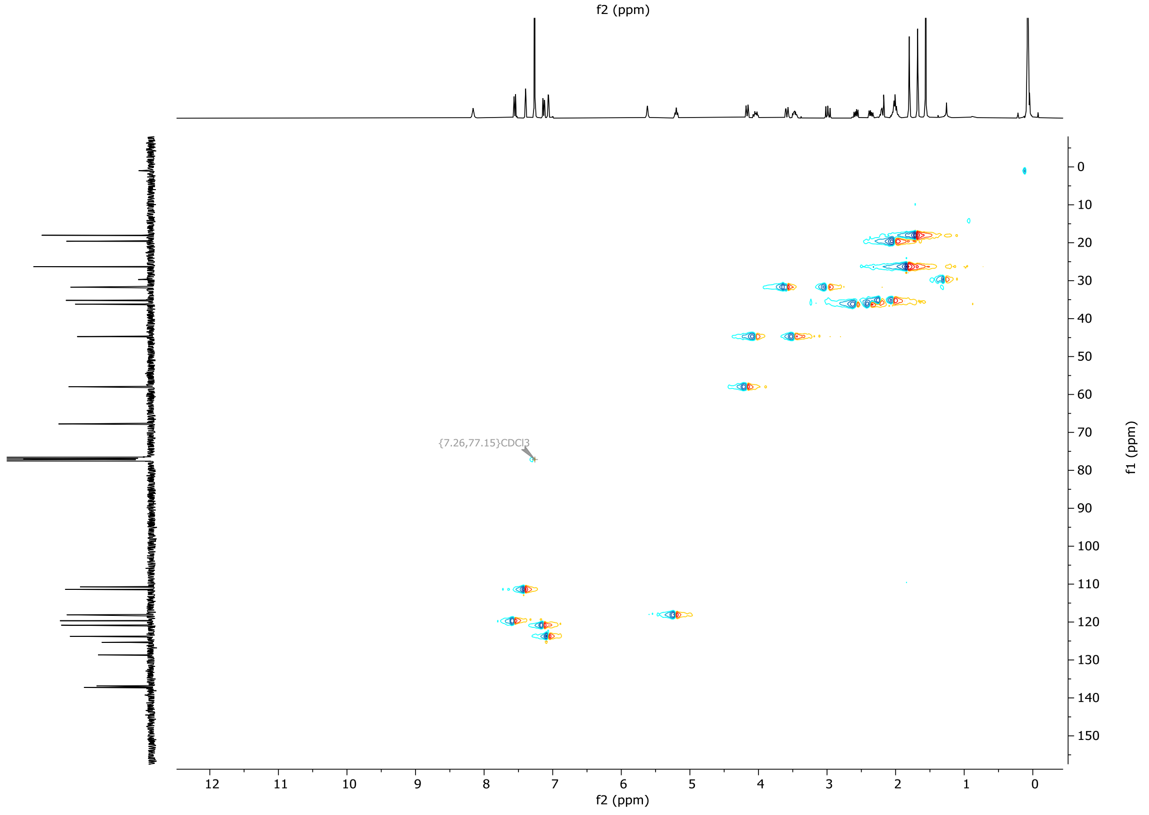

(3*S*,8a*S*)-3-((6-Chloro-1-(methoxymethyl)-1H-indol-3-yl)methyl)-2-(methoxymethyl)-8a-(3-methylbut-2-en-1-yl)hexahydropyrrolo[1,2-a]pyrazine-1,4-dione (8b) in CD3CN (400 MHz)

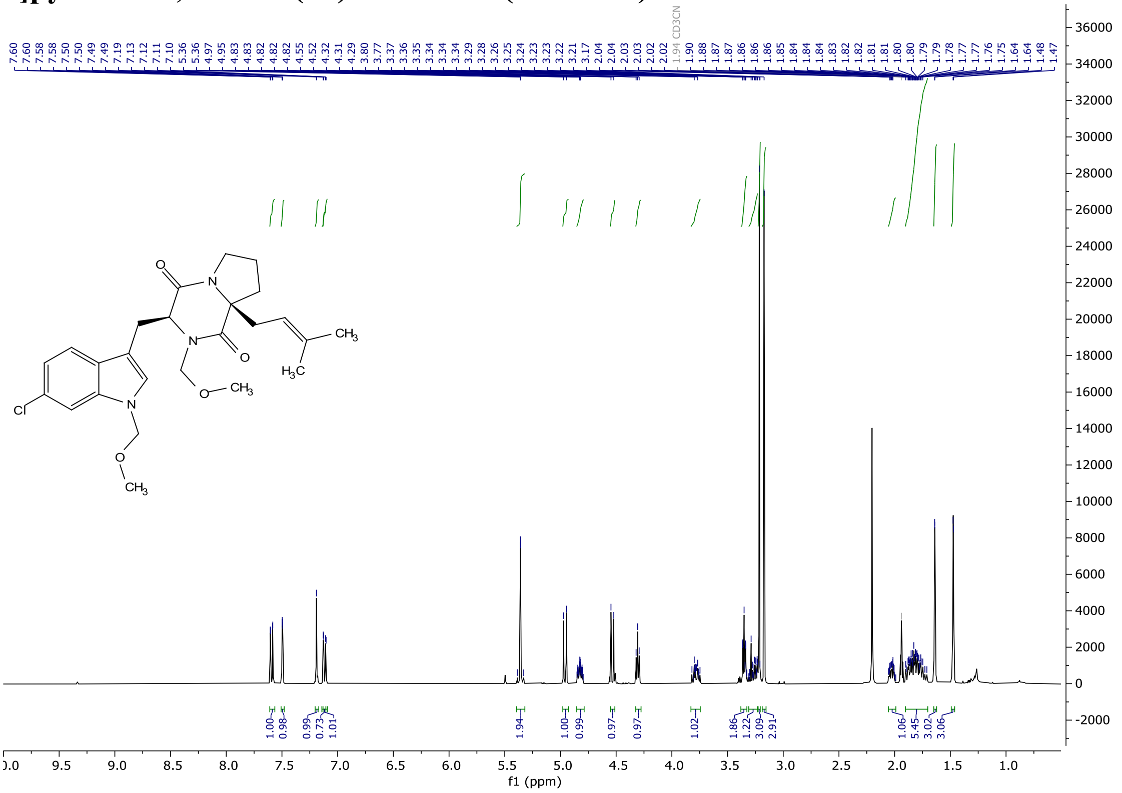

in CD3CN (100.6 MHz)

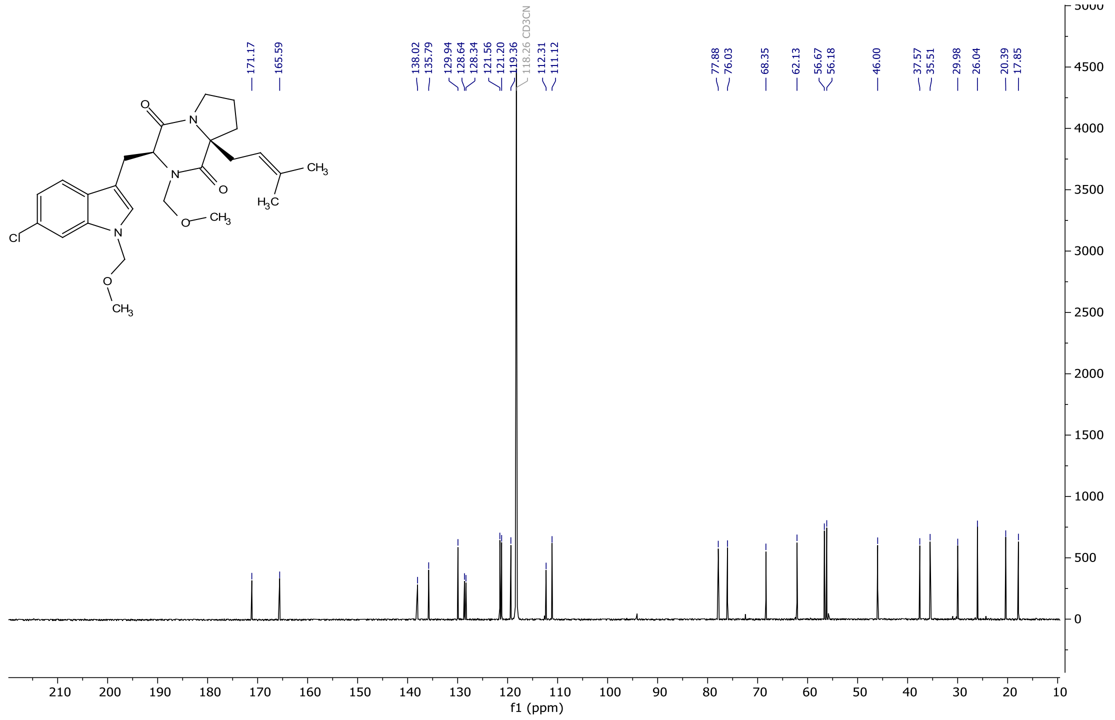

(3*S*,8a*S*)-3-((6-Chloro-1-(methoxymethyl)-1H-indol-3-yl)methyl)-2-(methoxymethyl)-8a-(3-methylbut-2-en-1-yl)hexahydropyrrolo[1,2-a]pyrazine-1,4-dione (8b) in CD_3CN

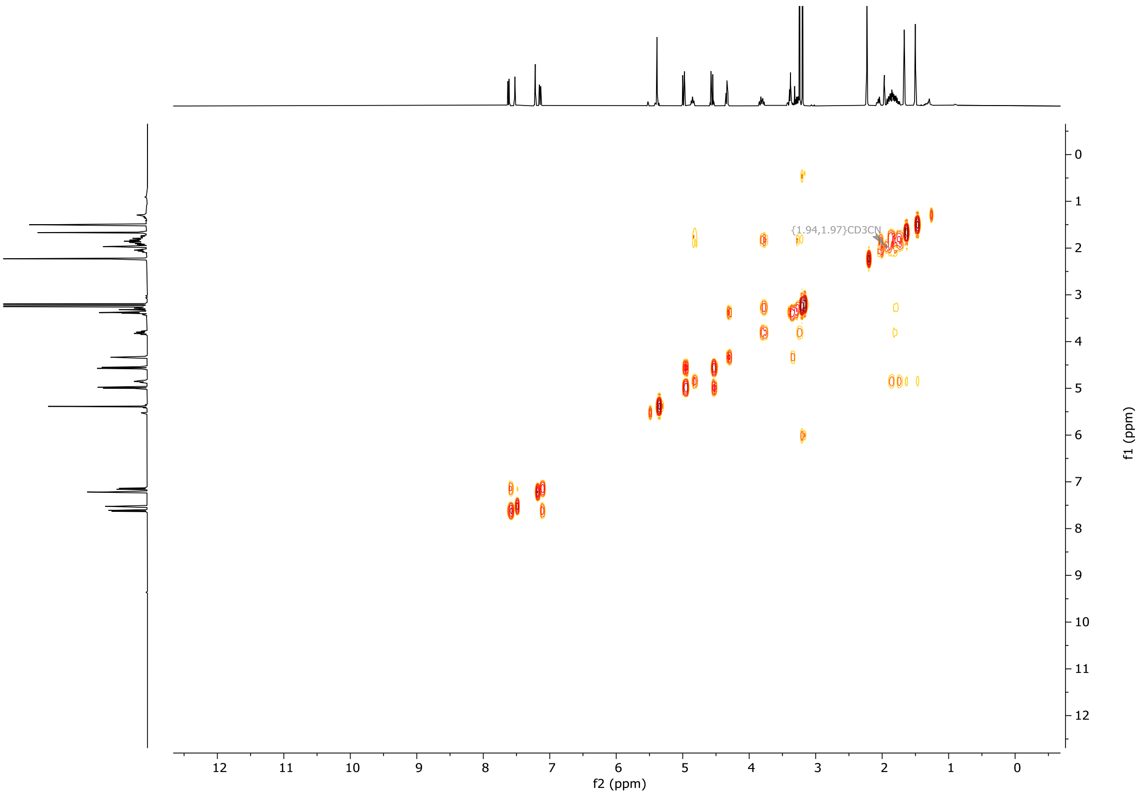

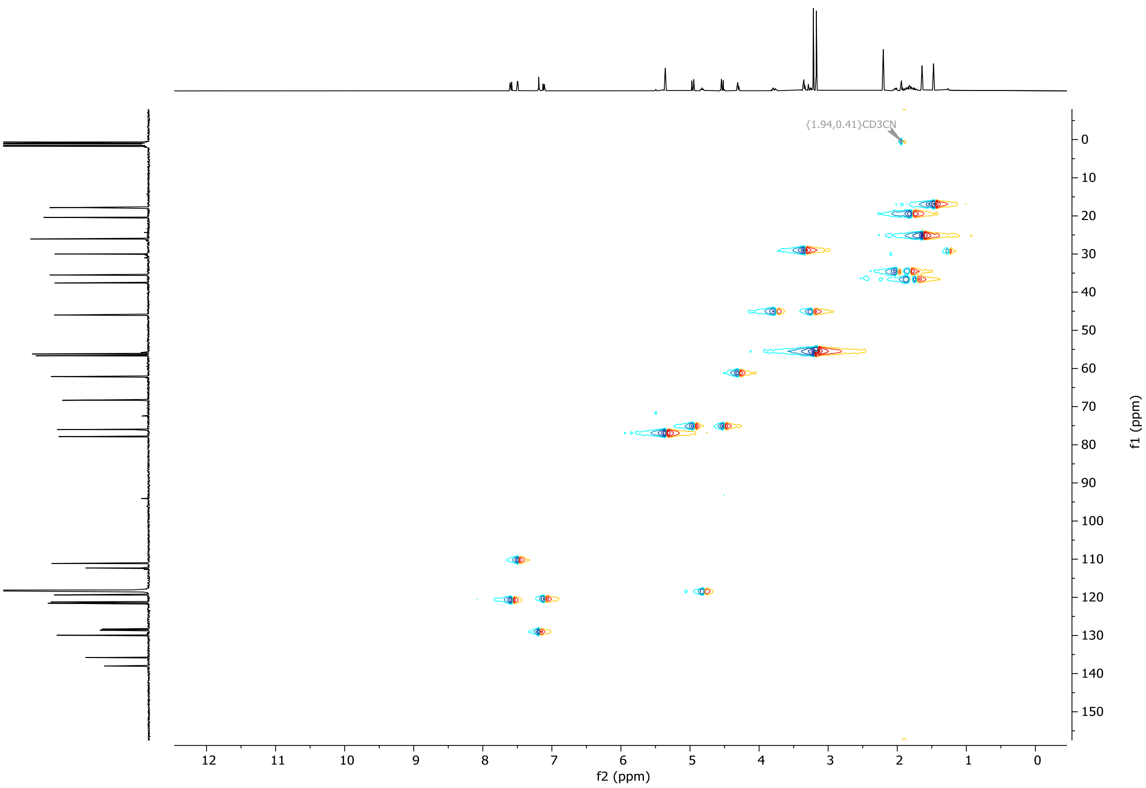

(6*S*,7*R*,8a*S*)-6-((6-Chloro-1-(methoxymethyl)-1H-indol-3-yl)methyl)-10-(methoxymethyl)-7-(prop-1-en-2-yl)tetrahydro-1*H*-6,8a(epiminomethano)indolizine-5,9(6H)-dione (15) in CD_3CN (400 MHz)

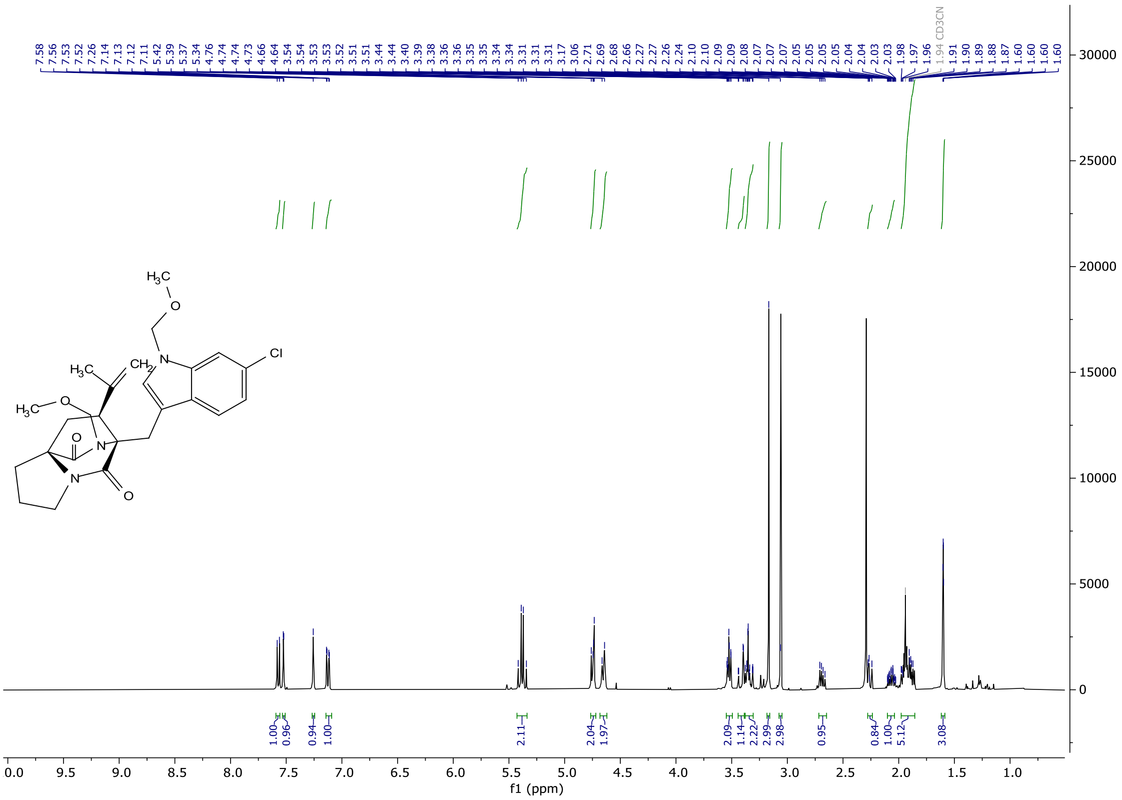

in CD_3CN (100.6 MHz)

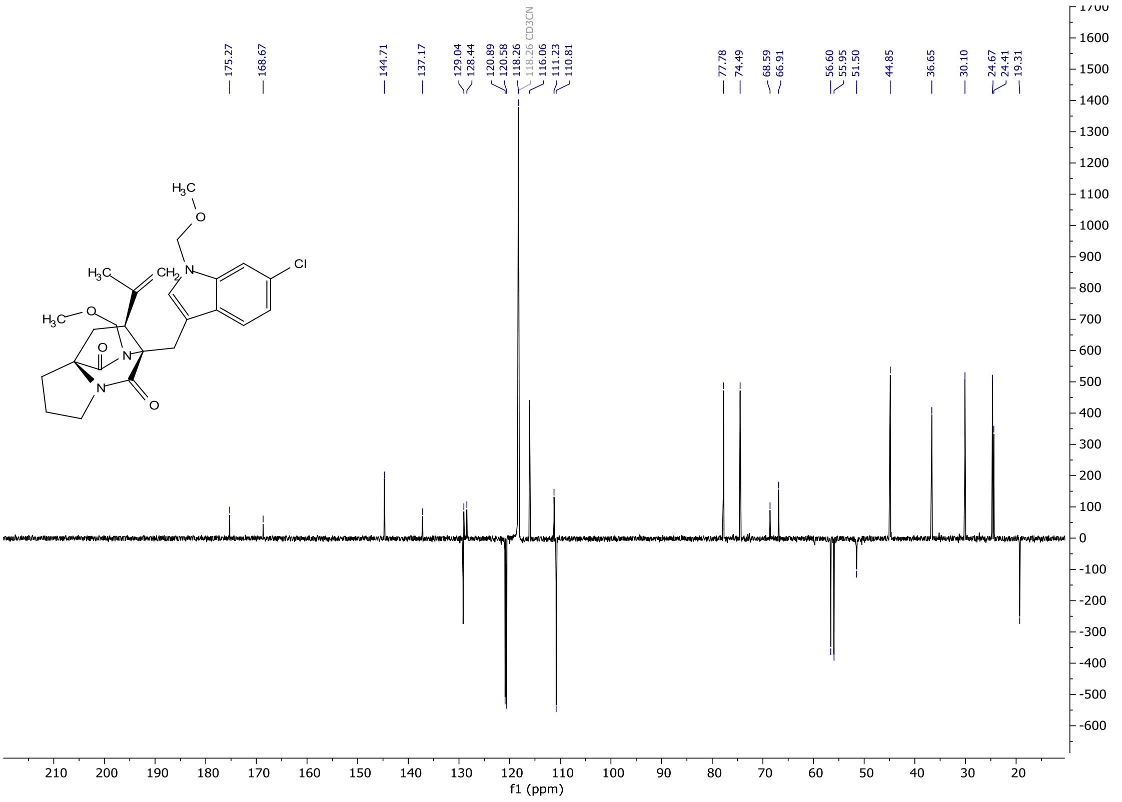

(6*S*,7*R*,8a*S*)-6-((6-Chloro-1-(methoxymethyl)-1H-indol-3-yl)methyl)-10-(methoxymethyl)-7-(prop-1-en-2-yl)tetrahydro-1*H*-6,8a(epiminomethano)indolizine-5,9(6H)-dione (15) in CD_3CN

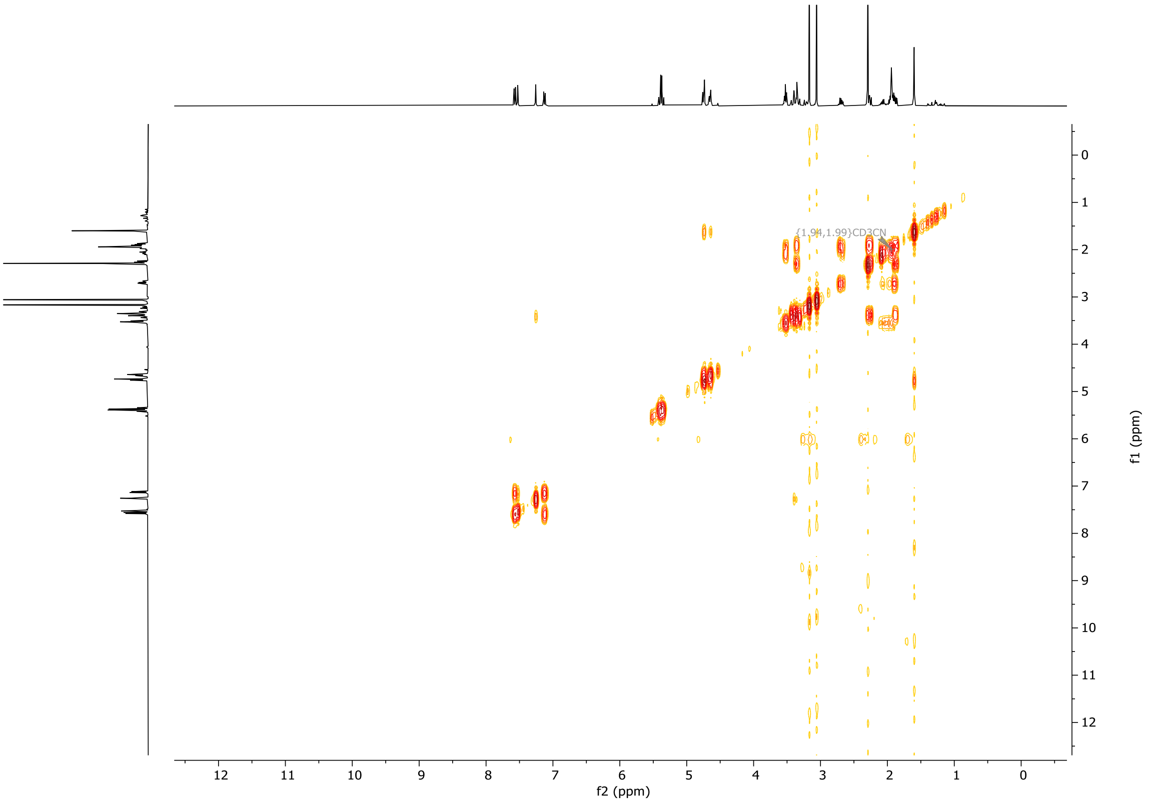

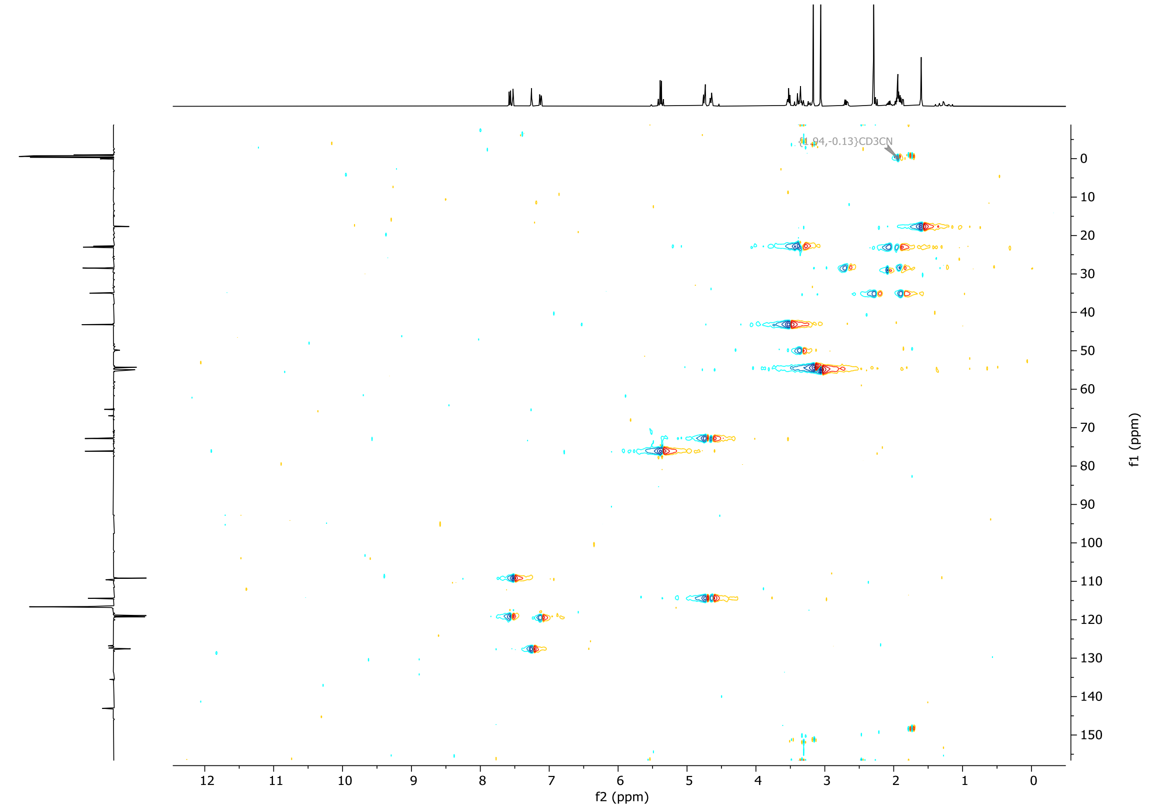

(6*S*,7*S*,8a*S*)-6-((6-Chloro-1-(hydroxymethyl)-1H-indol-3-yl)methyl)-7-(prop-1-en-2-yl)tetrahydro-1H-6,8a-(epiminomethano)indolizine-5,9-(6H)-dione (16) in CD_3OD (500 MHz)

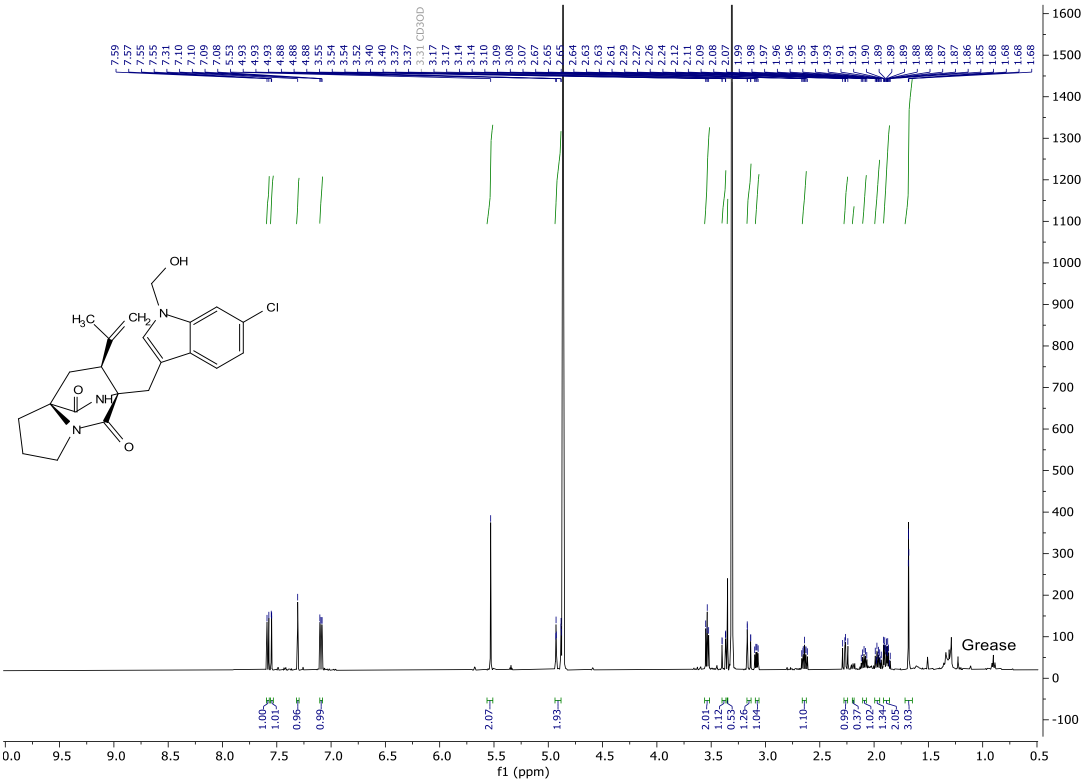

in CD_3OD (126 MHz)

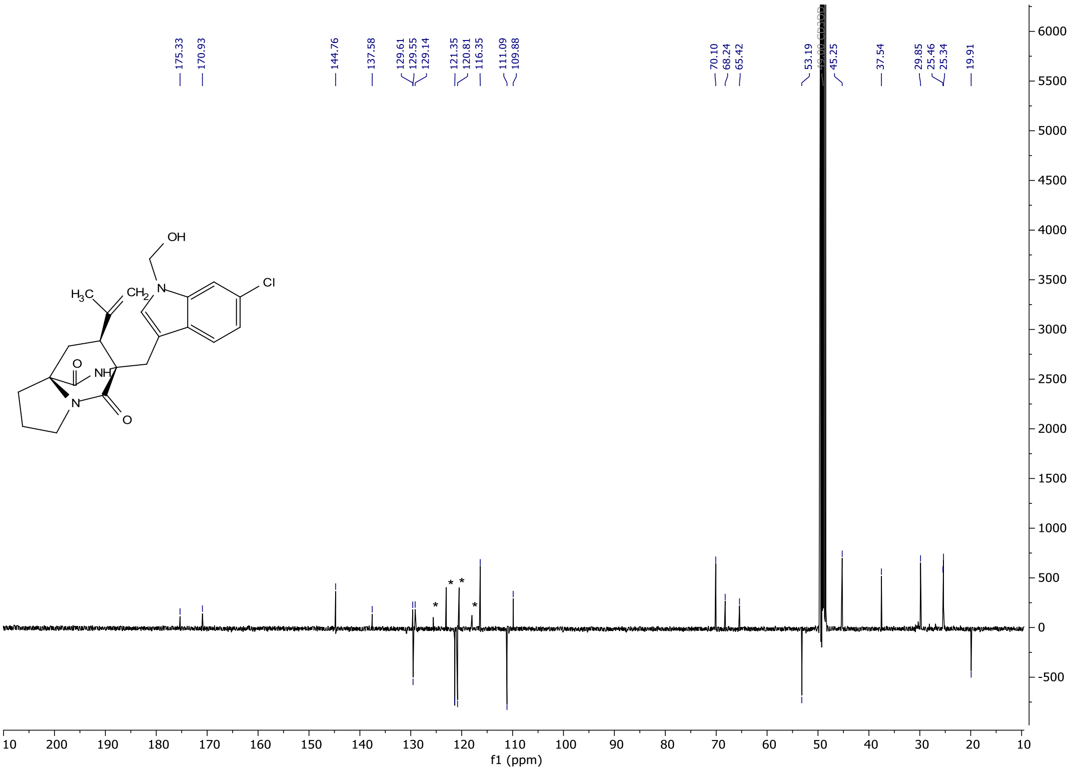

*The marked signals correspond to a CF_3 group. The origin is unknown; it is not part of the molecule and may be a byproduct of the MOM-deprotection reaction using TESOTf.

(6*S*,7*S*,8a*S*)-6-((6-Chloro-1-(hydroxymethyl)-1H-indol-3-yl)methyl)-7-(prop-1-en-2-yl)tetrahydro-1H-6,8a-(epiminomethano)indolizine-5,9-(6H)-dione (16) in CD_3OD

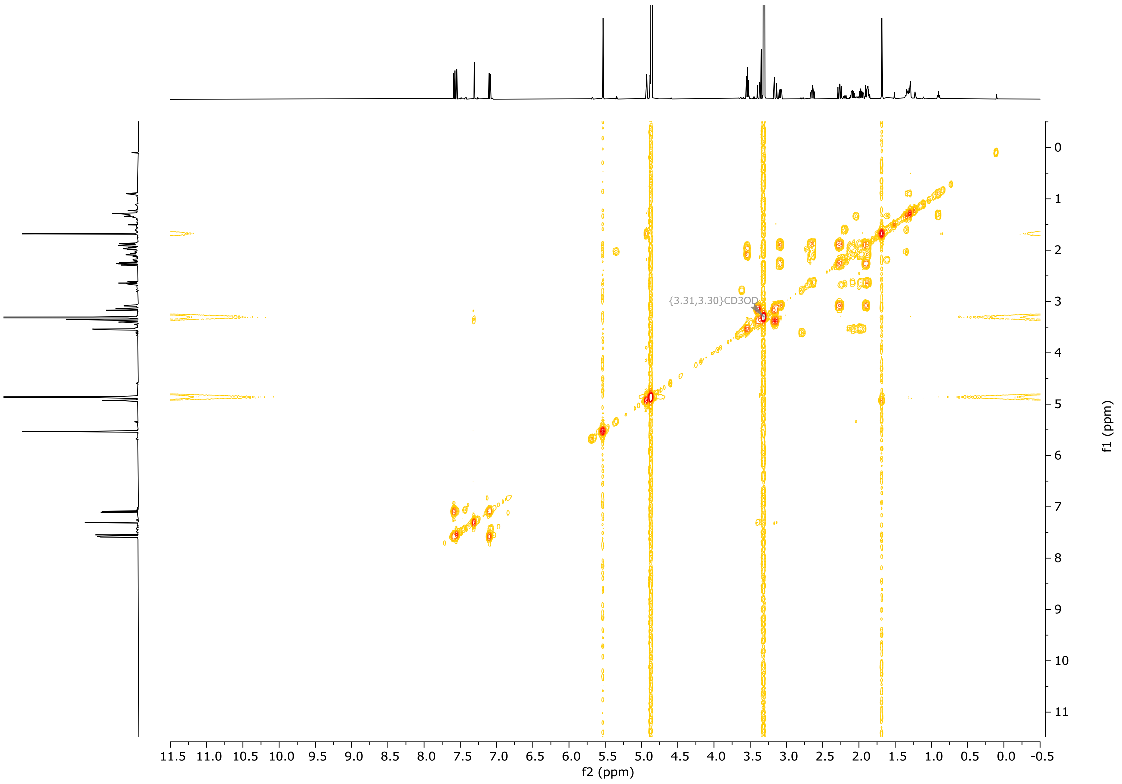

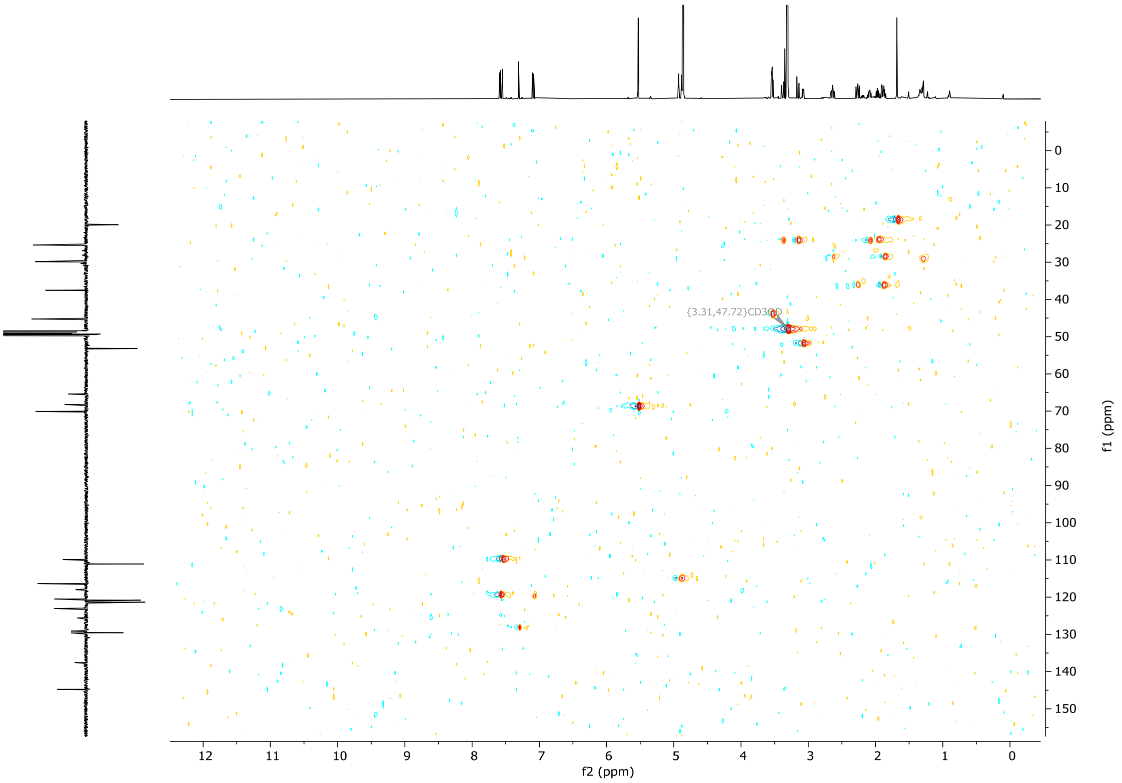

(5a*S*,12a*S*,13a*S*)-9-Chloro-12,12-dimethyl-2,3,11,12,12a,13-hexahydro-1H,5H,6H-5a,13a-(epiminomethano)indolizino[7,6-b]carbazole-5,14-dione (17) in CD_3CN (600 MHz)

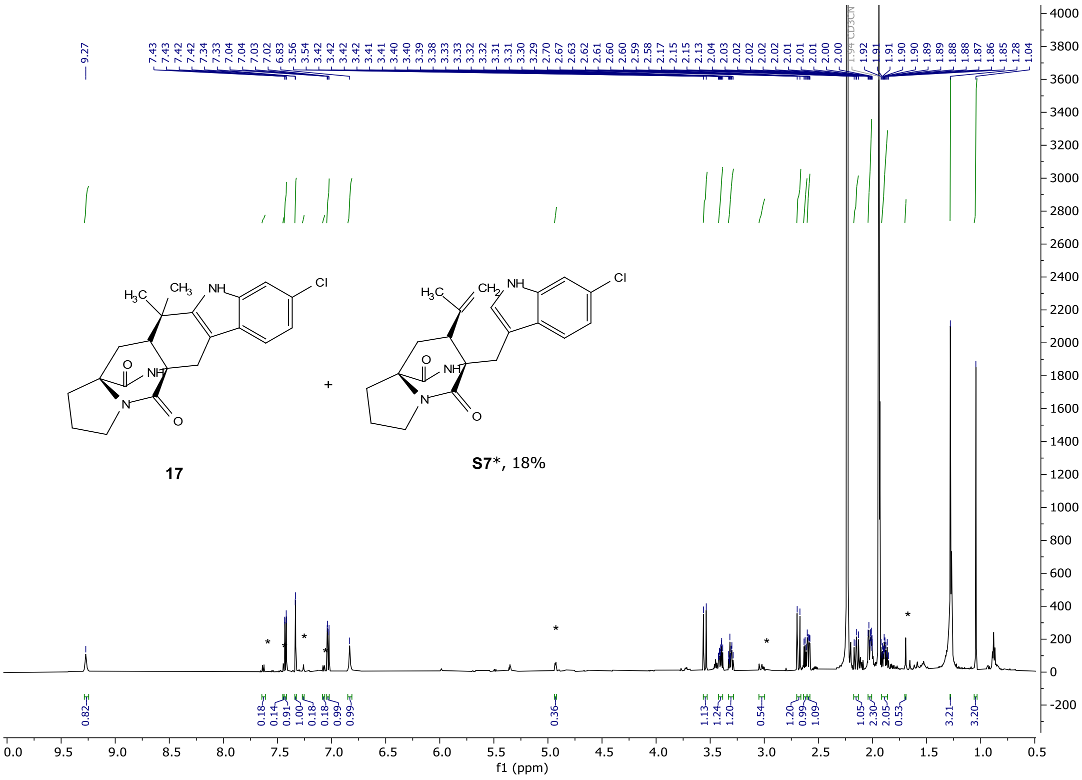

in CD_3CN (151 MHz)

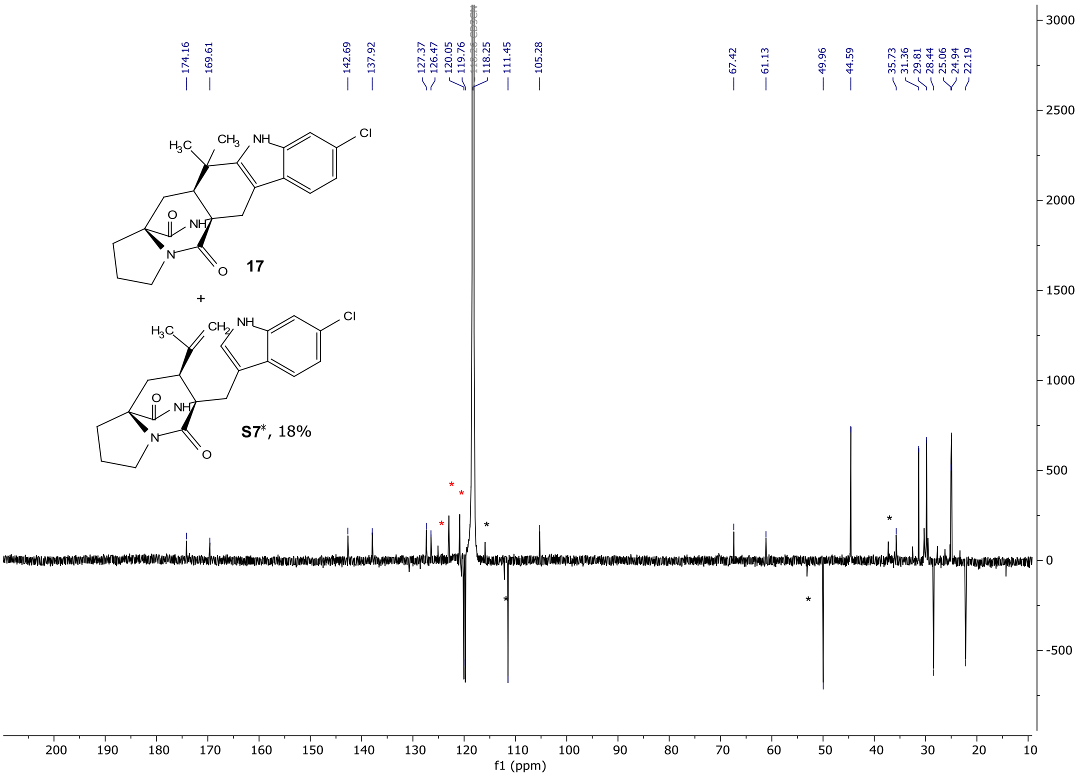

* The marked signals correspond to a CF_3 group. The origin is unknown; it is not part of the molecule and may be a byproduct of the MOM-deprotection reaction using TESOTf

(5a*S*,12a*S*,13a*S*)-9-Chloro-12,12-dimethyl-2,3,11,12,12a,13-hexahydro-1H,5H,6H-5a,13a-(epiminomethano)indolizino[7,6-b]carbazol-14-one (malbrancheamide B) (1) in CD3OD (600 MHz)

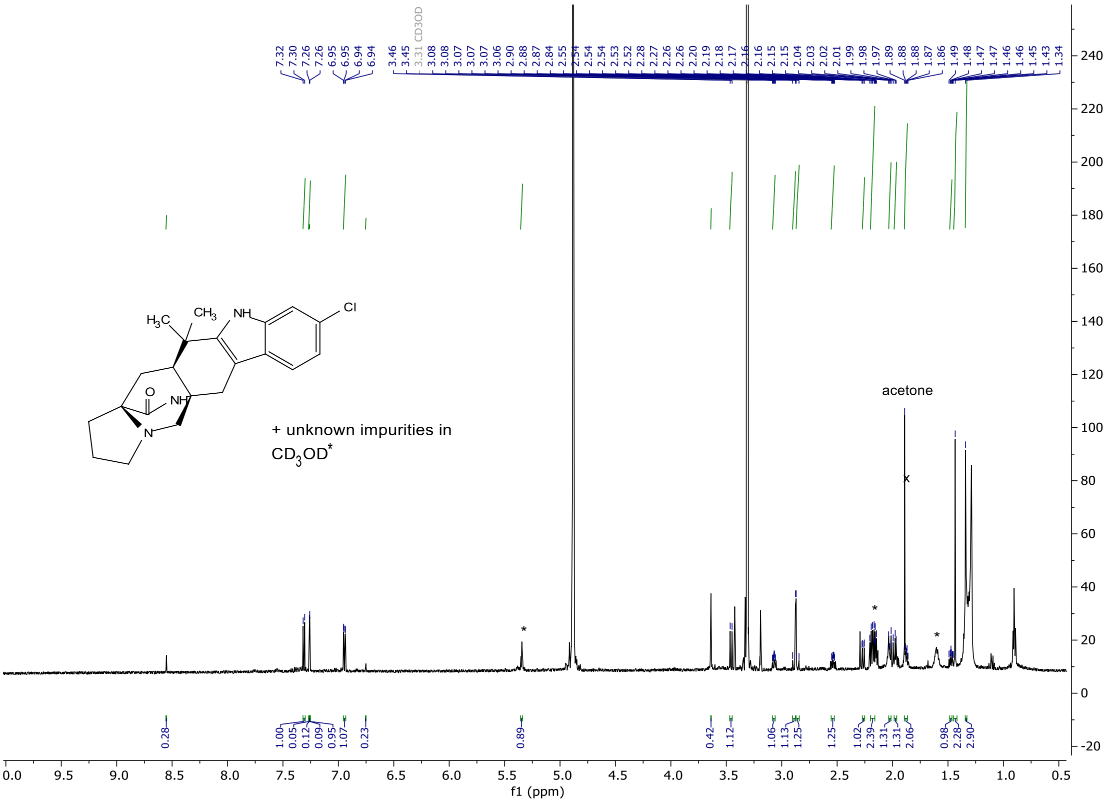

in CD3OD (126 MHz)

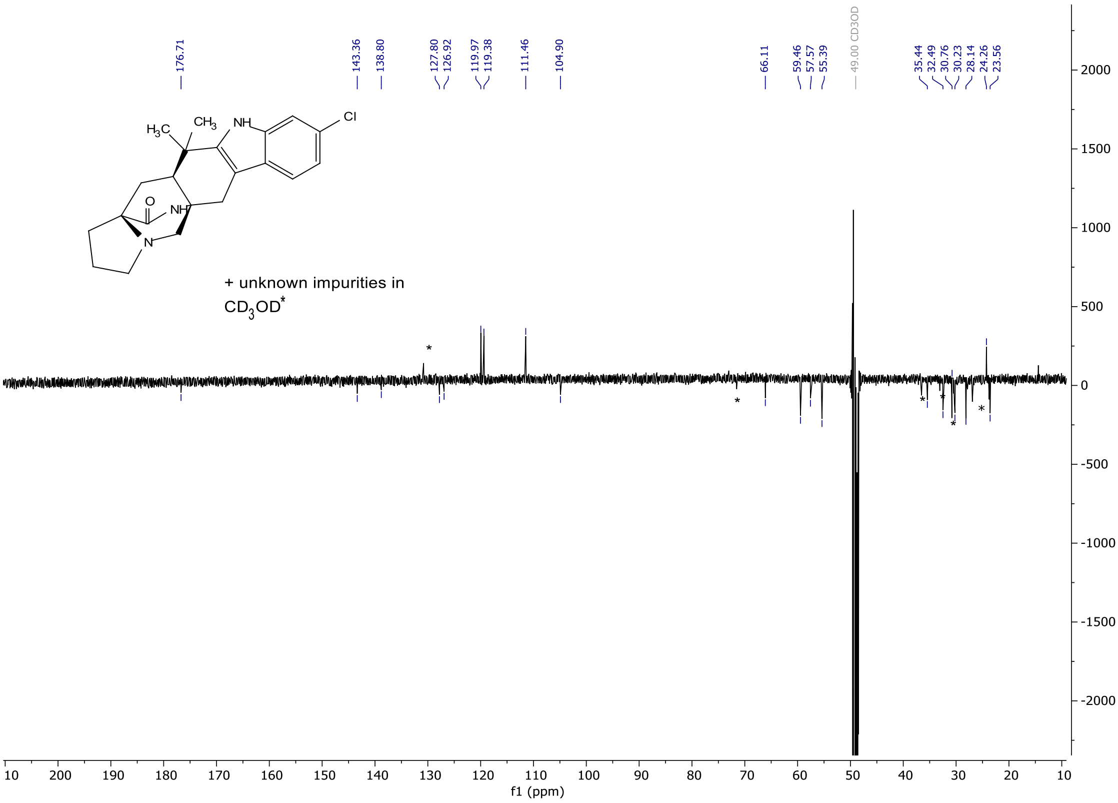

(5a*S*,12a*S*,13a*S*)-9-Chloro-12,12-dimethyl-2,3,11,12,12a,13-hexahydro-1H,5H,6H-5a,13a-(epiminomethano)indolizino[7,6-b]carbazol-14-one (malbrancheamide B) (1) in CD_3OD

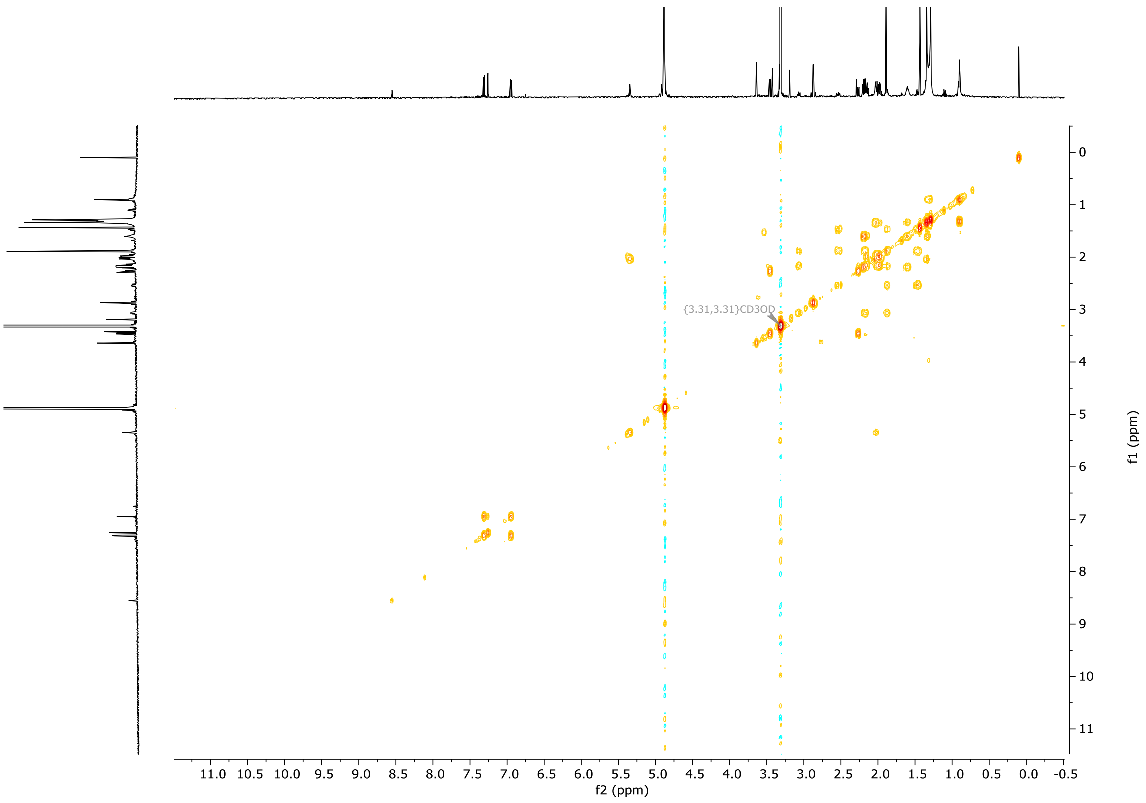

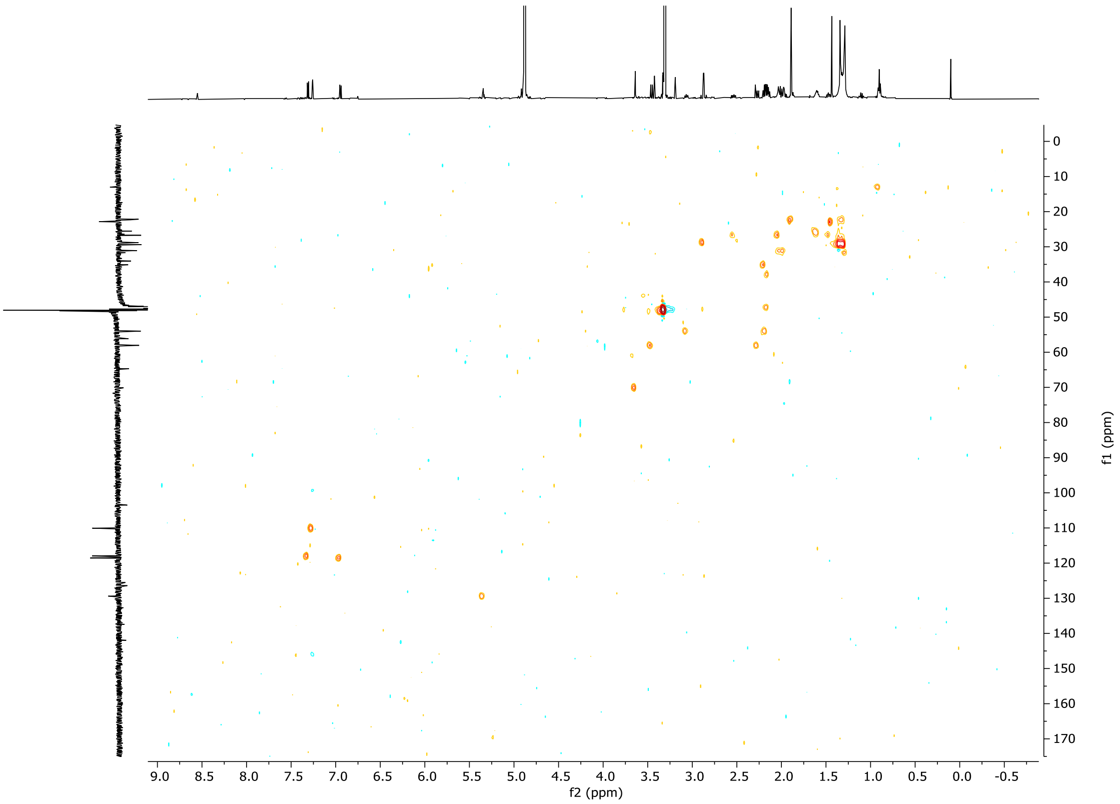

Blank spectrum of CD_3OD containing unknown impurities, probably plasticizer

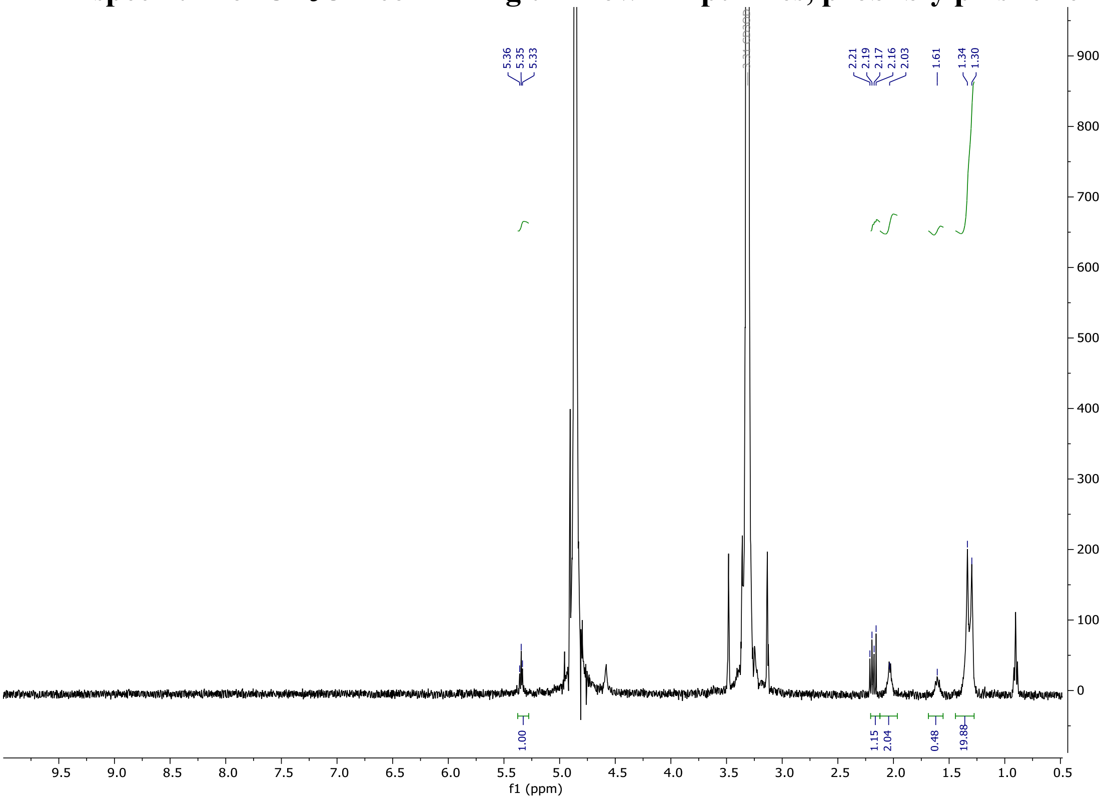

(5a*S*,12a*S*,13a*S*)-9-Chloro-12,12-dimethyl-2,3,11,12,12a,13-hexahydro-1H,5H,6H-5a,13a-(epiminomethano)indolizino[7,6-b]carbazol-14-one (malbrancheamide B) (1) in DMSO-d_6 (600 MHz)

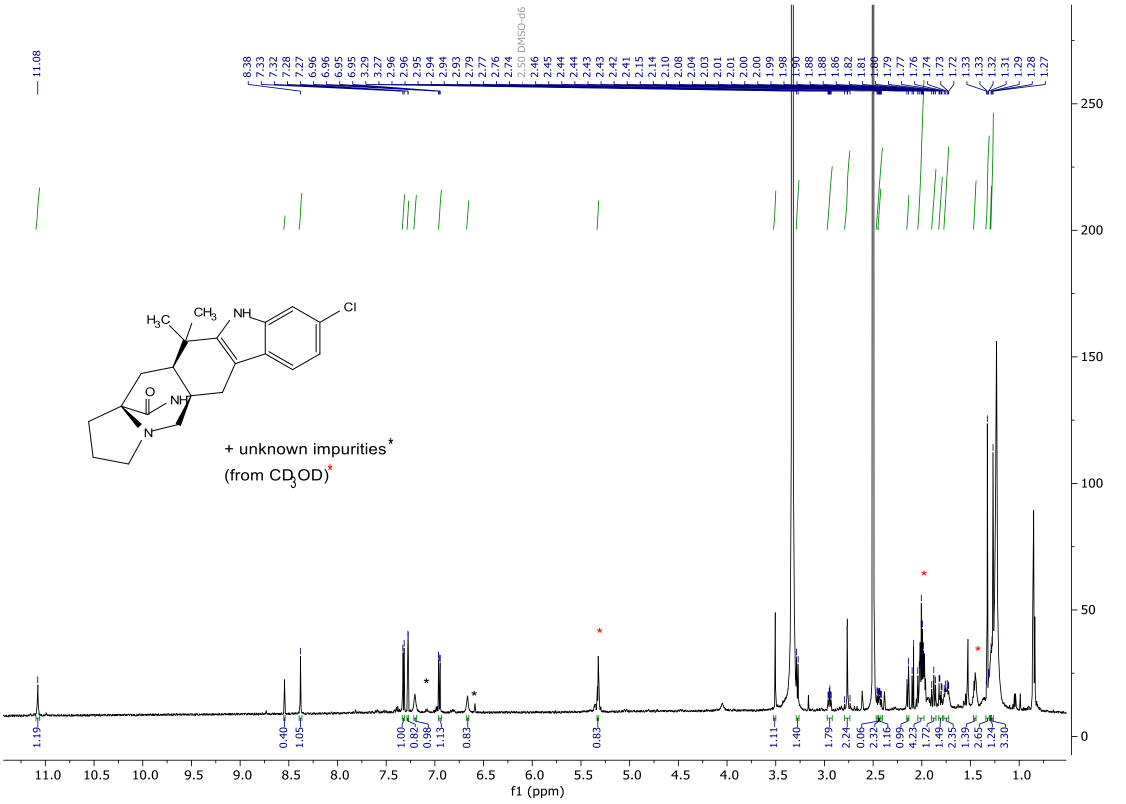

in DMSO-d_6 (151 MHz)

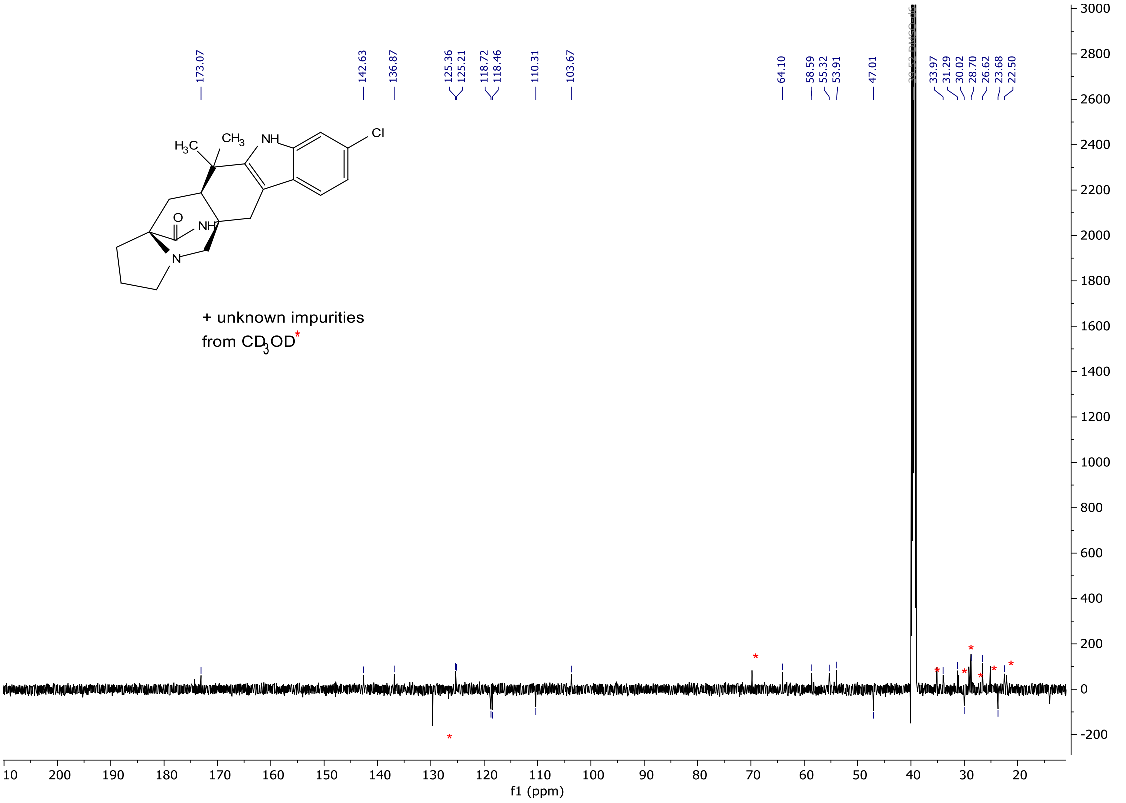

(5a*S*,12a*S*,13a*S*)-9-Chloro-12,12-dimethyl-2,3,11,12,12a,13-hexahydro-1H,5H,6H-5a,13a-(epiminomethano)indolizino[7,6-b]carbazol-14-one (malbrancheamide B) (1) in DMSO-d_6

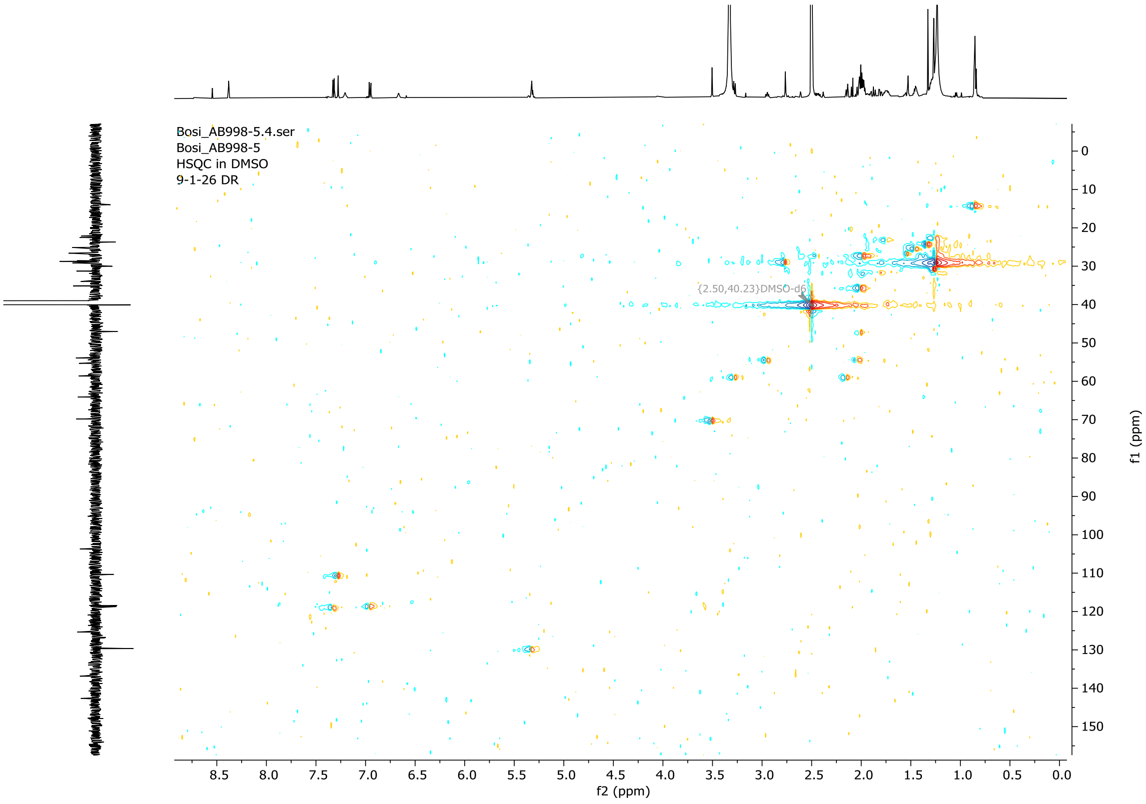

UPLC chromatogram of 1

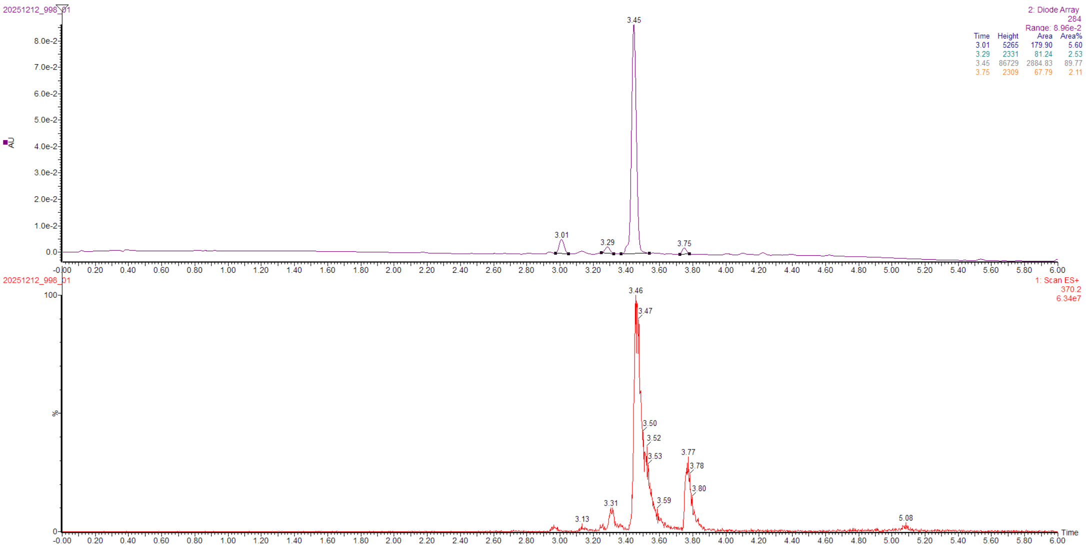

(5a*S*,6a*S*,11a*R*,13a*S*)-9-Chloro-13a-(3-methylbut-2-en-1-yl)-6a-((2,2,6,6-tetramethylpiperidin-1-yl)oxy)-1,2,3,6,6a,11,11a,13a-octahydro-13H-pyrrolo[1'',2'':4',5']pyrazino[1',2':1,5]pyrrolo[2,3-b]indole-5,13(5aH)-dione (20) in CD_3CN (500 MHz)

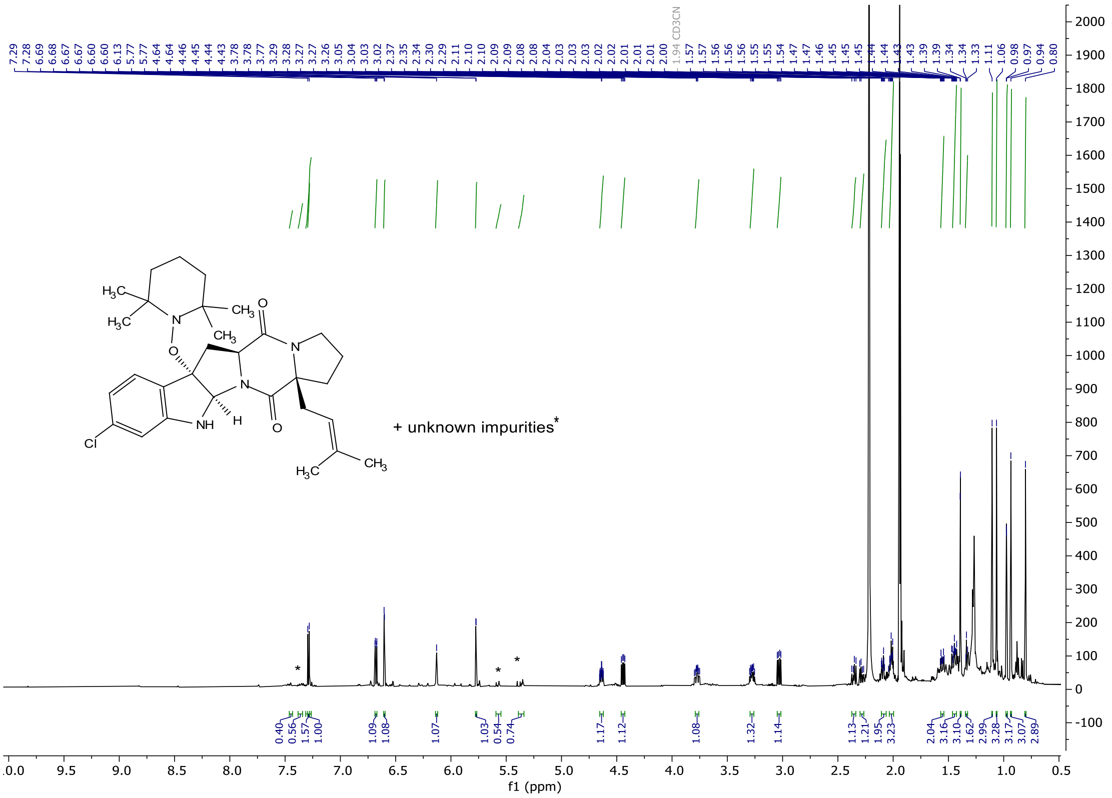

in CD_3CN (126 MHz)

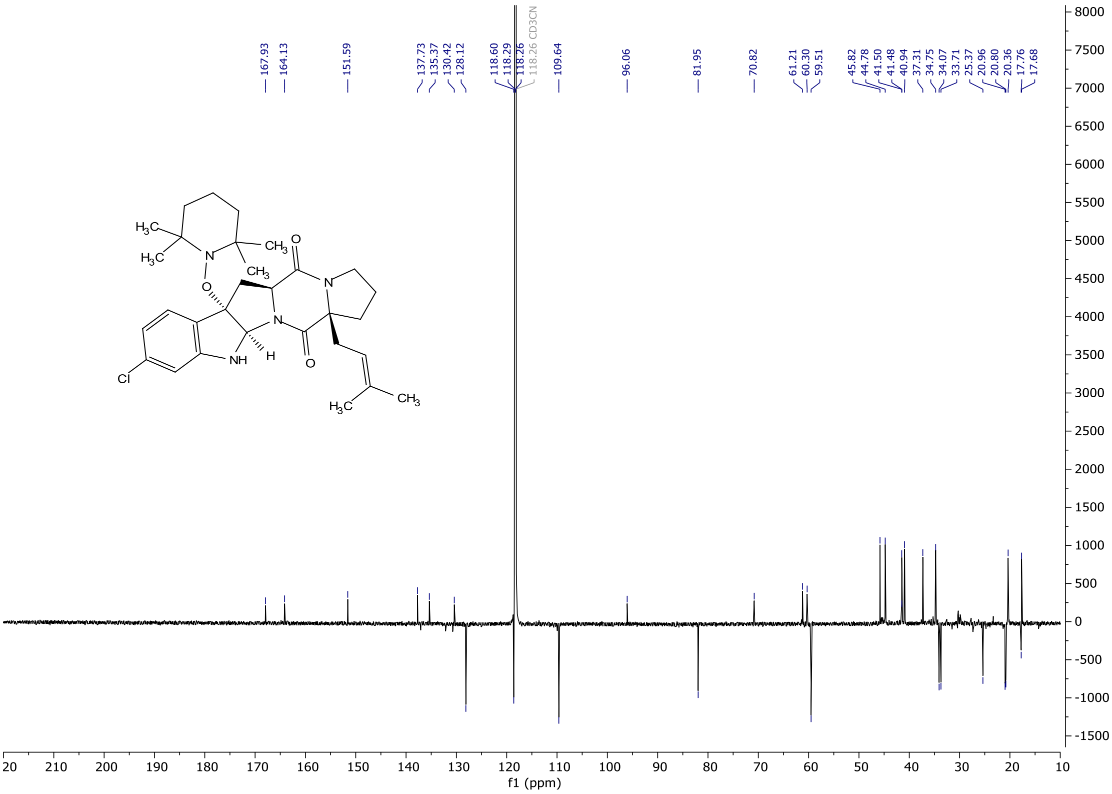

(5a*S*,6a*S*,11a*R*,13a*S*)-9-Chloro-13a-(3-methylbut-2-en-1-yl)-6a-((2,2,6,6-tetramethylpiperidin-1-yl)oxy)-1,2,3,6,6a,11,11a,13a-octahydro-13H-pyrrolo[1'',2'':4',5']pyrazino[1',2':1,5]pyrrolo[2,3-b]indole-5,13(5aH)-dione (20) in CD_3CN

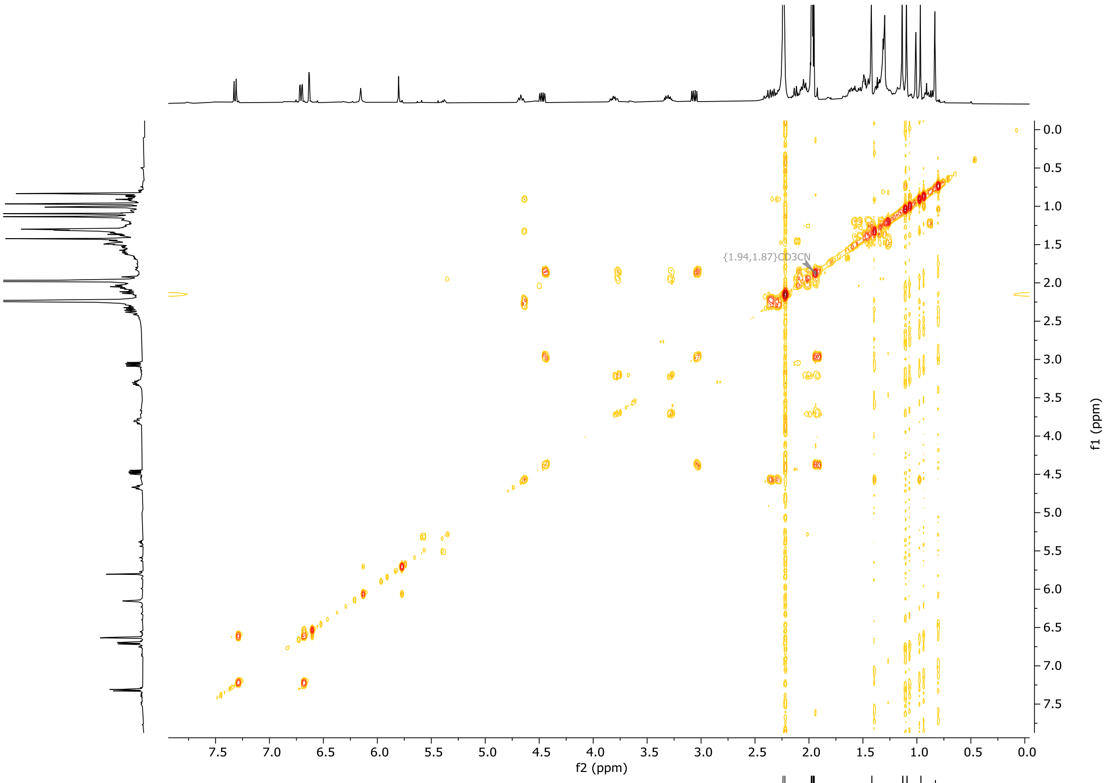

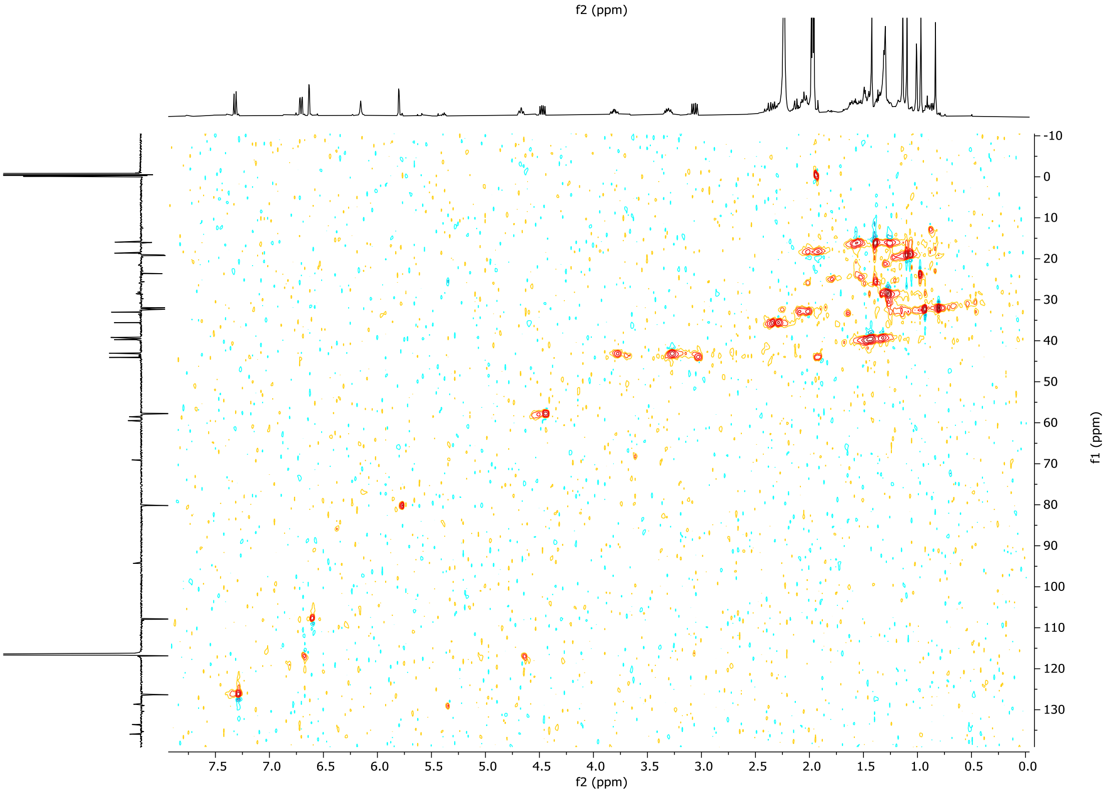

(5a*S*,14a*S*,16*S*)-9-Chloro-16-(prop-1-en-2-yl)-2,3,11,12-tetrahydro-1H,5H,6H,14H-5a,14a-ethanopyrrolo[1'',2'':4',5']pyrazino[1',2':1,6]pyrido[3,4-b]indole-5,14-dione (23) in CD3CN (500 MHz)

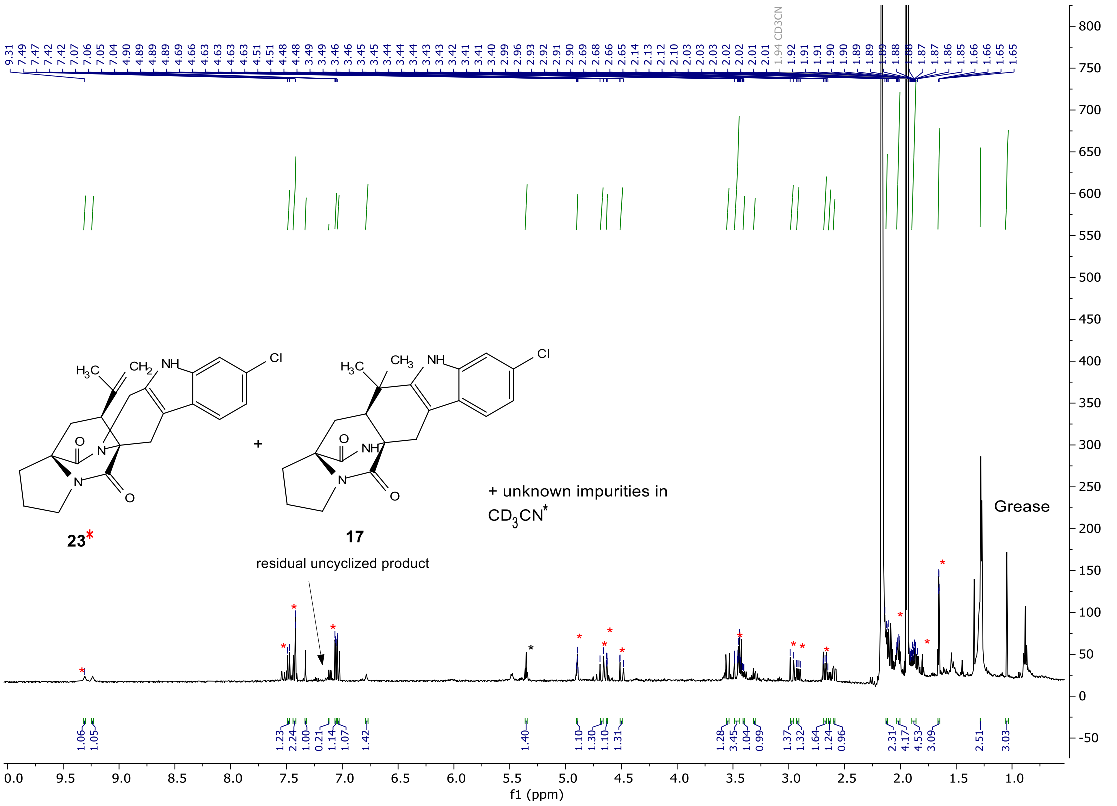

in CD3CN (126 MHz)

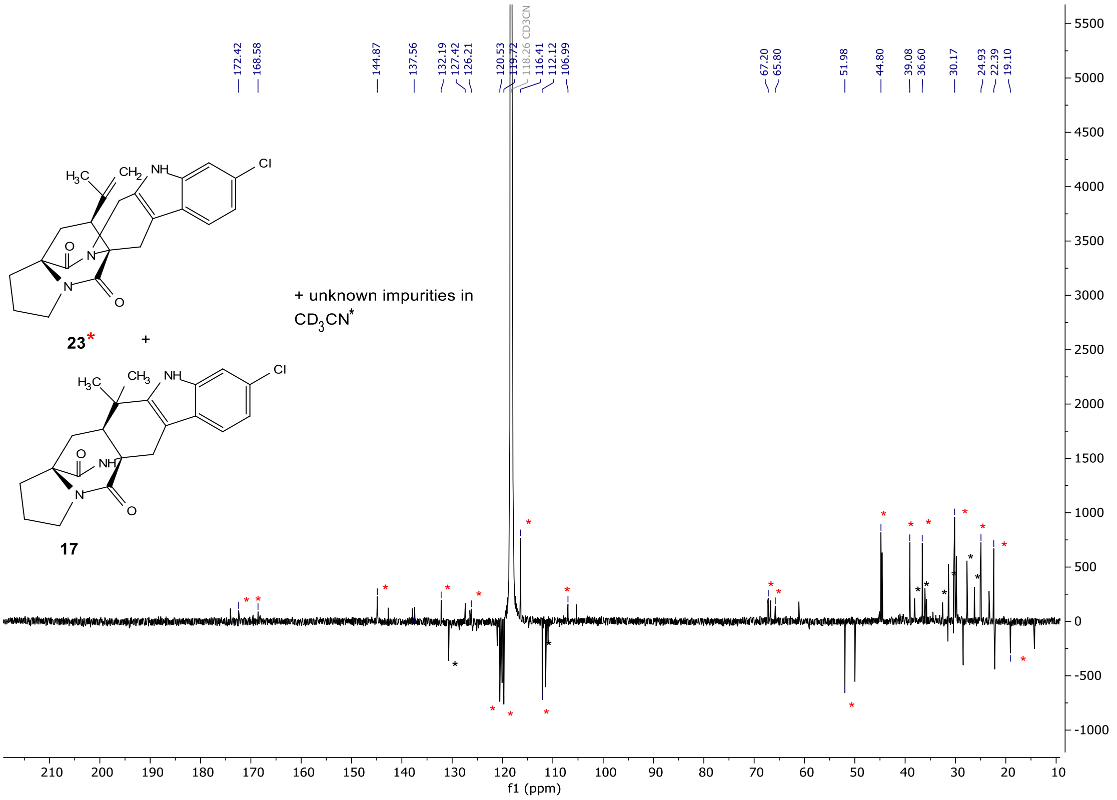

17/23 mixture in CD_3CN

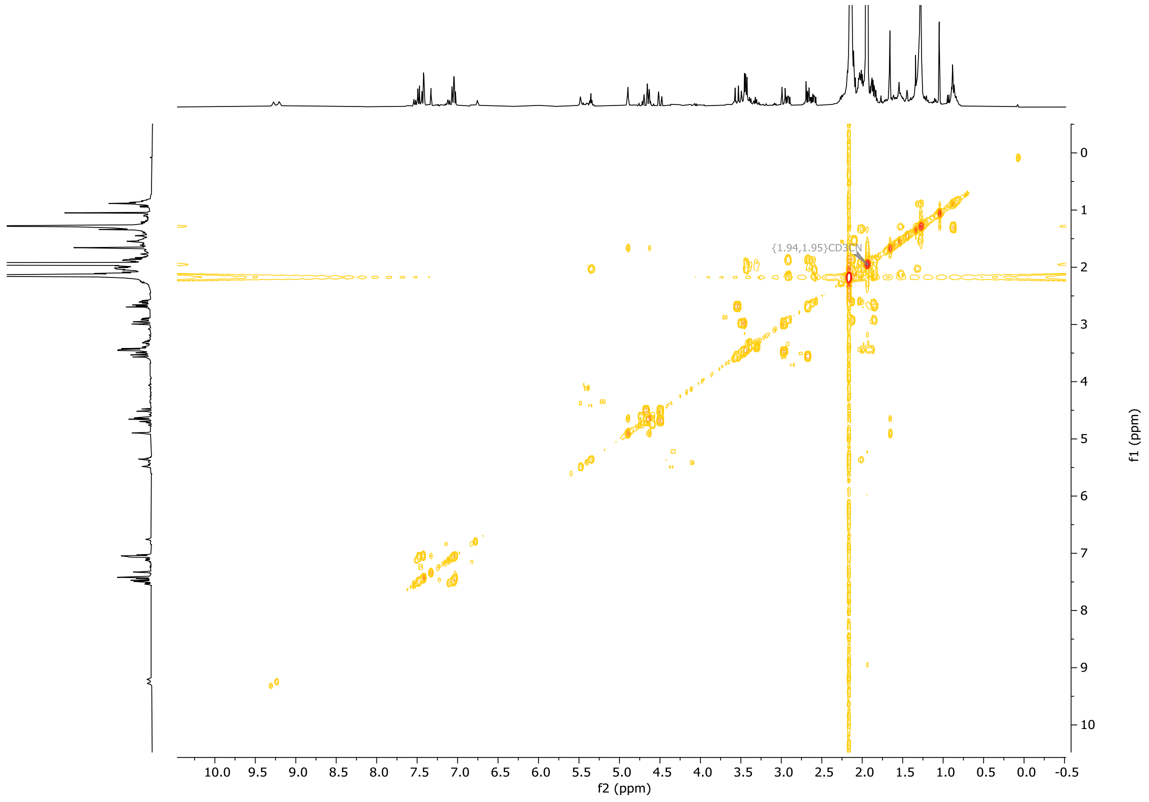

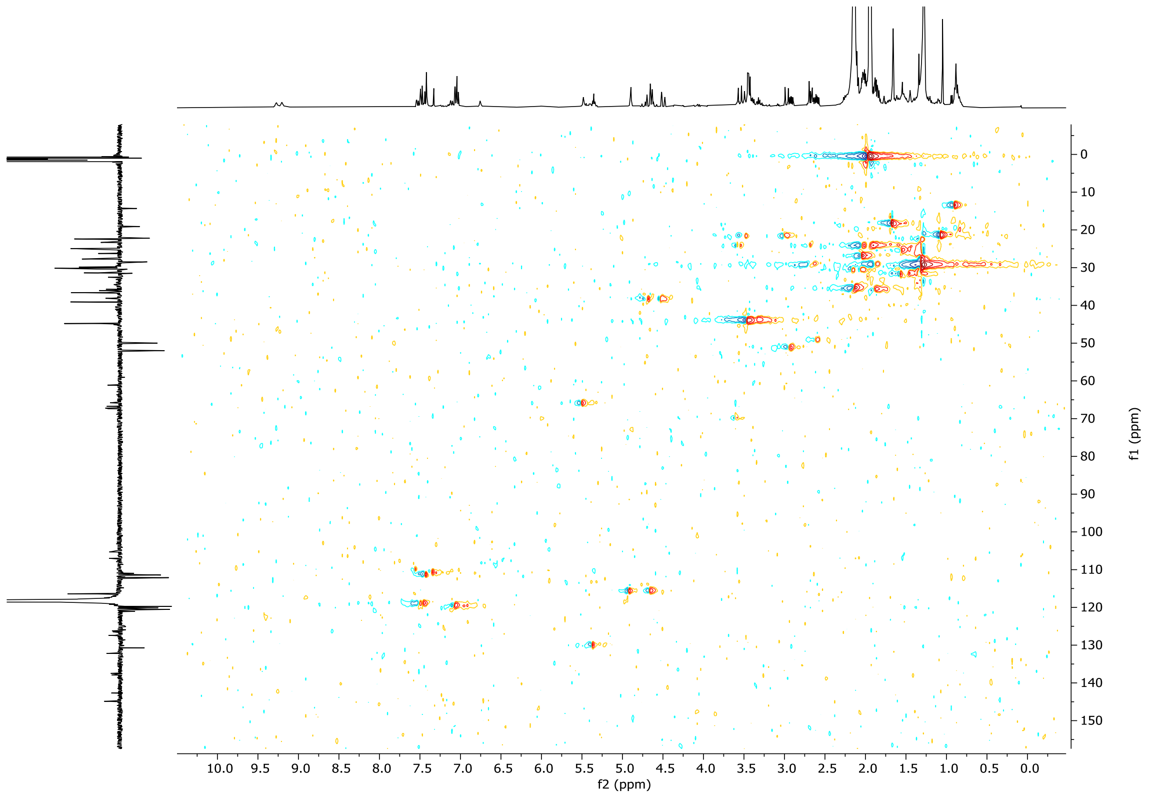

Blank spectrum of CD_3CN containing unknown impurities, probably plasticizer

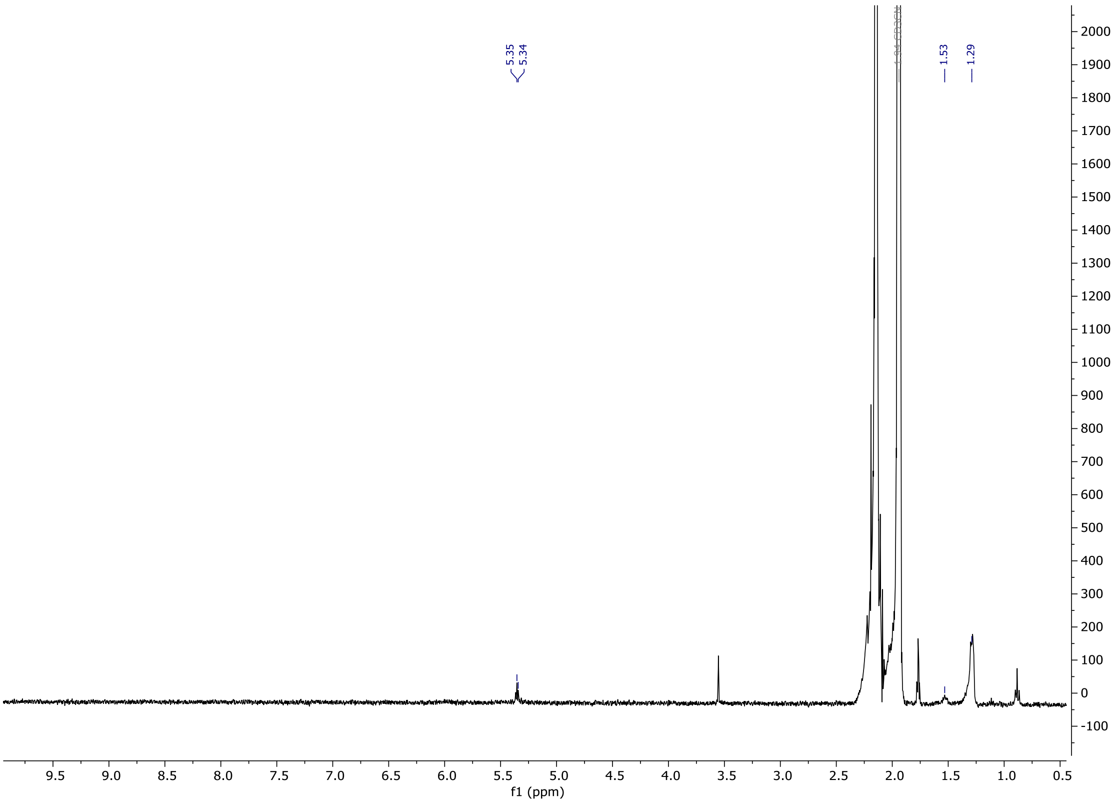

Supplement: Supplementary file 1 [file ol6c00246_si_001.pdf]
